# Hyperbranched, Functional Polyethoxysiloxanes: Tunable Molecular Building Blocks

**DOI:** 10.1021/acsapm.4c00758

**Published:** 2024-06-14

**Authors:** Marek Nemec, Stefanie B. Hauser, Daniel Rentsch, Gabriel M. Pagotti João, Lilli C. Kuerten, Nour Adilien, Lukas Huber, Ana Stojanovic, Wim J. Malfait, Matthias M. Koebel

**Affiliations:** † Laboratory of Building Energy Materials & Components, Swiss Federal Laboratories for Materials Science and Technology, Empa, Überlandstrasse 129, CH-8600 Dübendorf, Switzerland; ‡ Siloxene AG, Zürichstrasse 38, 8306 Brüttisellen, Switzerland; § Laboratory for Functional Polymers, Swiss Federal Laboratories for Materials Science and Technology, Empa, Überlandstrasse 129, CH-8600 Dübendorf, Switzerland

**Keywords:** coatings, emulsions, foams, FTIR, GPC, NMR spectroscopy, silanes TEOS

## Abstract

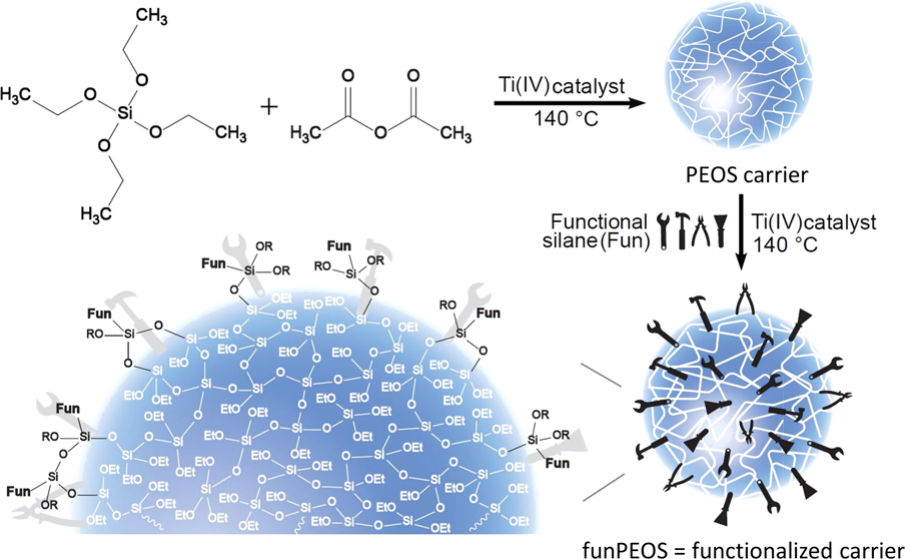

Functional silanes are multifaceted cross-linkers, compatibilizers, coupling agents, and surface modifiers. Herein, we present organofunctional polysiloxane building blocks that offer great versatility in terms of molecular weight, degree of condensation, and the choice and loading of organic substituent groups. The organofunctional polyethoxysilanes (funPEOS) are prepared in a one-pot, two-step process: synthesis of the PEOS carrier/substrate, followed by grafting a functional silane “shell”, both based on condensation with acetic anhydride. The reaction was optimized at the lab scale and scaled up to a 7 L reactor. The acetylation, condensation, and hyperbranched structure of the carrier were confirmed by ^29^Si NMR, while ^29^Si–^29^Si 2D INADEQUATE NMR provides strong evidence for the grafting of functional silanes onto the carrier (Q–T coupling). IR, ^1^H, and ^13^C NMR spectroscopy demonstrate that the functional groups remain intact. The molar mass can be tailored by stoichiometric control of the acetic anhydride to silane monomer ratio (*M*_*n*_ 3500–20,000 g/mol). The compounds are stable organic liquids with a long shelf life. Selected applications are presented: scratch-resistant coatings with water contact angles of ∼90°, stable water emulsions, and surfactant-free, mesoporous silica foams.

## 1 Introduction

Functional silanes (T-type silanes) are widely used for thermoplastics, resin and rubber processing, filler modification, sol–gel chemistry and coatings, and additives for adhesives and sealants.^1−11^ Organofunctional trialkoxysilanes are most commonly used in monomer form yet compete with prehydrolyzed “oligomeric” T-type silanes or polyhedral oligomeric silsesquioxanes (POSS) that provide increased performance at a significantly higher price point.

POSS are inorganic–organic additives with a cage-like architecture. The inner siloxane inorganic, polyhedric framework is covered by R-substituent groups.^12^ Examples of common substituent groups include aminopropyl, glycidyl, alkenyl, acrylate, and azide functionalities. POSS are typically 1–3 nm in size and find use as toughening agents in nanocomposites, electroluminescent materials, catalytic supports, biomedicals, epoxy resins, and optoelectrics.^12−18^ POSS are typically based on polyhedral cages but also include hemisilsesquioxane in partial cages, ladders, and random structures.^13,19^ While the selective and specific use of the variable groups and structures allows for a broad spectrum of uses, the high price of POSS is still a limiting factor.

Oligomeric and prehydrolyzed functional alkoxysilanes offer a less expensive alternative. They are prepared from T-type silane monomers R′-Si(OR)_3_ through a hydrolytic or one of several nonhydrolytic routes.^8,9,20,21^ Delattre and Babonneau^20^ studied hydrolysis–condensation for alkoxysilanes with different alkyl chains (R′ = CH_3_, C_2_H_5_, C_8_H_17_) and reported that condensation is faster, and the final degree of condensation is higher for shorter alkyl chains.^20^ This chain-length-dependent difference in kinetics complicates the preparation of co-condensates from different silanes, and the molecular structure of cohydrolysates is difficult to control. Aside from the electronic and steric hindrance effects of the R′ group, the process is affected by pH, precursor concentration, and solvent system.^22−24^ Another obstacle of this hydrolytic route is the shelf life as residual silanol groups can cause further condensation reactions and subsequent changes in material properties or undesired precipitates.^25^

A recent entry in the world of silane-based molecular building blocks is hyperbranched polyalkoxysiloxanes (hyPASs), which offer a high functionality control for typical molecular weights ranging from 500 to 50,000 g/mol, corresponding to sizes from several Å to a few nm.^26,27^ The compact hyperbranched structure is highly advantageous in exhibiting low solution viscosities and a much higher solubility than their linear analogues.^26,28,29^

Several routes produce hyPASs in a neat, solvent-free system. (1) The silanol route, based on the reaction of an alkoxysilane with a strong alkali base, is rather impractical at the industrial scale due to large quantities of caustic alkali hydroxides and the resulting payload for waste product disposal.^21,26^ (2) Alkyl halide elimination route uses condensation of, e.g., chlorosilanes with alkoxysilanes, but its industrial uptake is limited by the corrosive nature of the chlorosilane and the harmful alkyl halide side products.^30^ (3) The ether route condenses a single alkoxysilane with itself by ether elimination and presents a safety hazard due to the formation of dialkyl ethers.^31,32^ (4) The acetoxy route condenses rather costly acetoxyfunctional alkoxysilane with itself by elimination of the corresponding acetic acid ester.^26^ (5) The anhydride route is similar to the acetoxy route but uses acetic anhydride as an active condensation reagent to form acetoxyfunctional-silane intermediates in situ, which then continue to undergo siloxane condensation. This method represents the most advanced method for hyPAS preparation in terms of scalability, safety, and ease of implementation and thus was selected as the condensation chemistry in the present study. The anhydride route originally invented by Muzafarov, Möller, and co-workers has been investigated in detail for the condensation of tetraethyl orthosilicate (TEOS) into hyperbranched polyethoxysilanes (PEOS). This technology was explored further with monosodiumoxyorganoalkoxysilanes,^33^ hyperbranched polymethylsilsesquioxanes,^34,35^ and mono- (M) and tetra- (Q) functional siloxanes,^36^ but not for the molecular building blocks consisting of Q-substrates decorated with T-type functional silanes presented in this study.

Herein, we explore functional polyethoxysilane (funPEOS) molecular building blocks produced by grafting a functional T-type alkoxysilane shell onto a hyperbranched PEOS carrier (also referred to as PEOS substrate) using the acetic anhydride route (5) to obtain a dendritic funPEOS with active and accessible organic functional groups distributed uniformly on the exterior shell periphery. funPEOS are molecular liquids that offer significant processing advantages over other silane blends or POSS due to their low solution viscosity, nonflammable characteristics, and generally easy handling.

## 2 Mechanism, Average Degree of Condensation, and Functionalization

Hyperbranched polyethoxysilanes (PEOS) prepared via one-pot, two-step reaction (acetylation and condensation) have been investigated in detail by Muzafarov, Möller, and co-workers (Scheme S2).^26,27^ This acetic anhydride route is quantitative in terms of its reagents (alkoxide and acetic anhydride), and therefore the amount of new bonds formed and, hence, the degree of condensation represented by the average number of bridging oxygen atoms (BO) per Si atom and average molecular weight (*M*_w_), scale directly with the stoichiometry of the reagents. Silicon is in tetrahedral coordination (sp^3^ hybridization), and each Si atom is coordinated by 4 oxygen atoms, which may or may not be shared with next-nearest neighbor Si atoms depending on the degree of condensation. Following the nomenclature from the glass science field, we denote the average number of bridging oxygen atoms per Si atom as BO/Si, which is a measure of how many alkoxy groups are converted into siloxane bridges, spanning from BO/Si = 0 for monomeric TEOS to BO/Si = 4 for fully condensed SiO_2_. Note that although each Si atom in silica has 4 BO neighbors, these BO are shared with its Si next-nearest neighbors, leading to a SiO_2_ stoichiometry for a BO/Si ratio of 4. The degree of condensation depends on the stoichiometry of the reagents: for example, an Ac_2_O/Si = 0.8 ratio corresponds to an acetic anhydride to tetra-alkoxysilane (e.g., TEOS) molar ratio of 0.8:1, which leads to BO/Si = 1.6 or a 40% degree of condensation. As a second example, an Ac_2_O/Si = 2 ratio would lead to a BO/Si of 4, i.e., the complete conversion of all Si atoms to SiO_2_. A complete conversion, however, is practically not possible, as gelation typically occurs at Ac_2_O/Si ratios above ∼1.2.^27^

In terms of the reaction mechanism, acetic anhydride reacts with a free alkoxy moiety to yield an acetoxy intermediate under the elimination of an acetic acid ester side product. In a second step, the acetoxy intermediate undergoes condensation with a second alkoxy group Si-OR from another Si atom to form a siloxane bridge, Si–O–Si, under the elimination of a second acetic acid ester. The acetic acid esters have a low boiling point compared with the reaction conditions (typically 140 °C), e.g., 57 and 77 °C for methyl and ethyl acetate, respectively, and thus rapidly evaporate from the reaction mixture. In fact, the reaction progress can be observed by monitoring the flow of the condensate. Both acetylation and condensation reactions are catalyzed well by group 4 transition metals, especially organotitanium compounds, which are to-go-to catalysts for transesterification of silicon tetraalkoxides.^27,37^ Dynasylan 40 (D40) (Evonik) and other commercial TEOS oligomers with a silica content near 40 wt % (compared with 28 wt % in TEOS) present a noticeable advantage since fewer Si–O–Si linkages need to be formed to obtain a PEOS substrate structure with a given BO/Si. This reduces the reaction time, the amount of acetic anhydride consumption, and the amount of ethyl acetate side product.

**Scheme 1:**
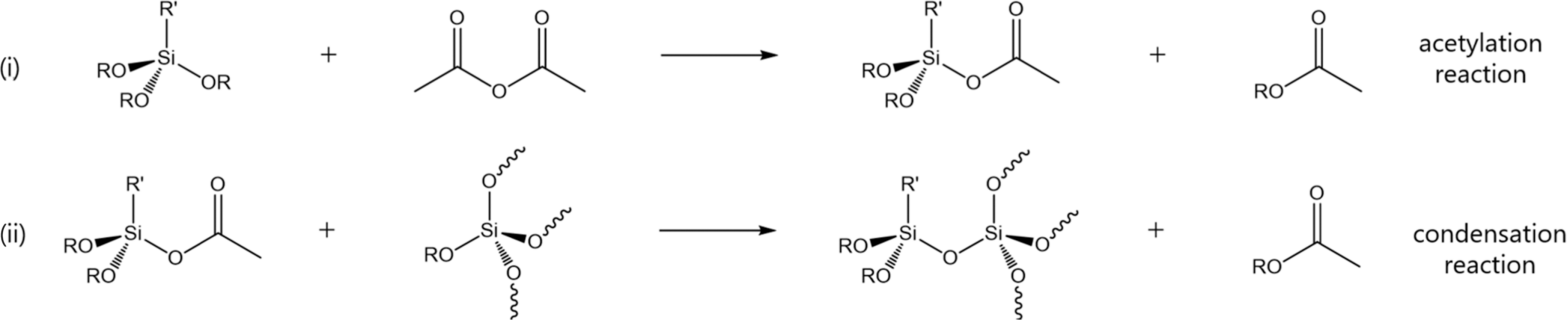
One-pot Two-step Grafting of a Functional Trialkoxysilane on a PEOS Carrier

The “shell” grafting of organofunctional silanes onto the PEOS carrier can be accomplished using the same acetylation-condensation mechanism but has not been described before. This reaction has a lower theoretical maximum BO/Si because of the lower number of alkoxy groups (2 for di- or 3 for trialkoxysilanes). In this study, we focus on grafting functional silanes onto preassembled PEOS carriers according to Scheme 1. The resulting T^1^, T^2^, T^3^, D^1^, D^2^, or M^1^ species (Scheme S1) can be quantified by ^29^Si NMR, where T*^n^* is a Si atom coordinated by *n* bridging oxygen atoms, 3-*n* nonbridging oxygen atoms, and 1 carbon atom of its R′ group, D*^n^* is a Si atom coordinated by *n* bridging oxygen atoms, 2-*n* nonbridging oxygen atoms, and 2 carbon atoms, and M^1^ is a Si atom coordinated by 1 bridging oxygen and 3 carbon atoms.

Complete stoichiometric control over the reaction allows full control of the degree of condensation and therefore full control of BO/Si. BO/Si_carrier,theoretical_ (eq 1) is directly proportional to the stoichiometric Ac_2_O/Si factor due to the quantitative conversion^26,27^ and experimentally, the effective BO/Si_carrier_ can be obtained from the ^29^Si NMR Q*^n^* speciation data (eq 2).
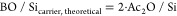
1
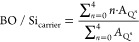
2where *A*_Q*^n^*_ is the ^29^Si NMR peak area of the Q*^n^* species. Q*^n^* represents a Si atom coordinated by *n* bridging oxygen atoms and 4-*n* nonbridging oxygen atoms.

The theoretical degree of functionalization (DF) is defined as the molar ratio of organofunctional silane (T, D, or M) to the Q-type siloxane starting material: *n*_shell_/*n*_carrier_. The monomeric organofunctional silane is added together with a selected amount of acetic anhydride to enable grafting on the carrier. One should note that during shell growth, the addition of acetic anhydride may lead to further carrier polymerization (Q–Q condensation) causing an increase in BO/Si_carrier_, homopolymerization of T species (T–T condensation) causing an increase in BO/Si_shell_, and grafting of organofunctional alkoxysilanes onto the previously synthesized PEOS carrier (Q–T condensation) affecting both BO/Si_carrier_ and BO/Si_shell_. Because BO/Si_carrier_ increases during the grafting step, a liquid PEOS carrier with BO/Si < 2.4 must be selected to avoid solidification, particularly for a high DF (>0.2) and high Ac_2_O/Si ratio (>1.3). Quantitative ^29^Si NMR data can be used to determine BO/Si of the synthesized final product according to eqs 2 and 3 and DF according to eq 4.
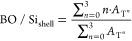
3
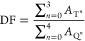
4where *A*_T*^n^*_ is the ^29^Si NMR peak area. An equation similar to eqs 3 and 4 can be applied for shells consisting of D or M functional silanes using *A*_D*^n^*_ and *A*_M*^n^*_ instead of *A*_T*^n^*_, and with summation up to *n* = 2 and *n* = 1, respectively.

**Figure 1:**
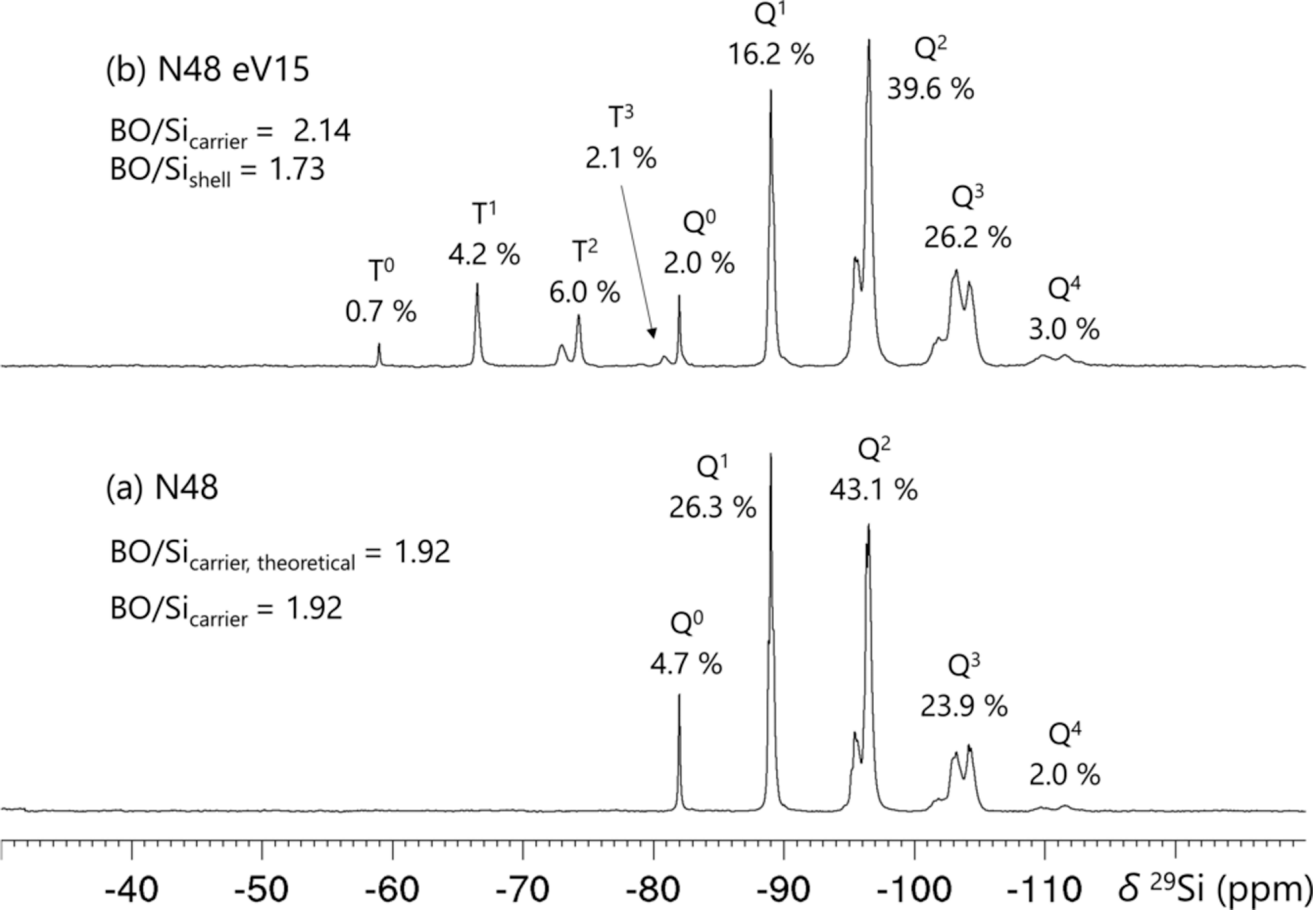
^29^Si NMR spectra with signal assignments, relative intensities, and BO/Si values obtained from (a) PEOS carrier and (b) funPEOS carrier decorated with shell containing vinyl functionality.

Figure 1 depicts typical ^29^Si NMR spectra of PEOS and funPEOS. Initially, a TEOS-based hyperbranched PEOS carrier with BO/Si_carrier_ = 1.92 was first synthesized followed by grafting a vinyl-functionalized shell (from VTES). BO/Si_carrier_ increased to 2.14 due to Q-Q and Q–T condensation. The nomenclature (Table S3) is explained by this example: N48 eV15 represents a molecular polysiloxane (N) with a targeted BO/Si_carrier,theoretical_ of 1.92, i.e., 48% of the theoretical maximum (therefore N48), functionalized with vinyl groups introduced by adding VTES (i.e., a silane with vinyl group and ethoxy groups, therefore eV) in an *n*_shell_/*n*_carrier_ ratio of 0.15/1.

## 3 Results and Discussion

### 3.1 PEOS Synthesis

**Figure 2:**
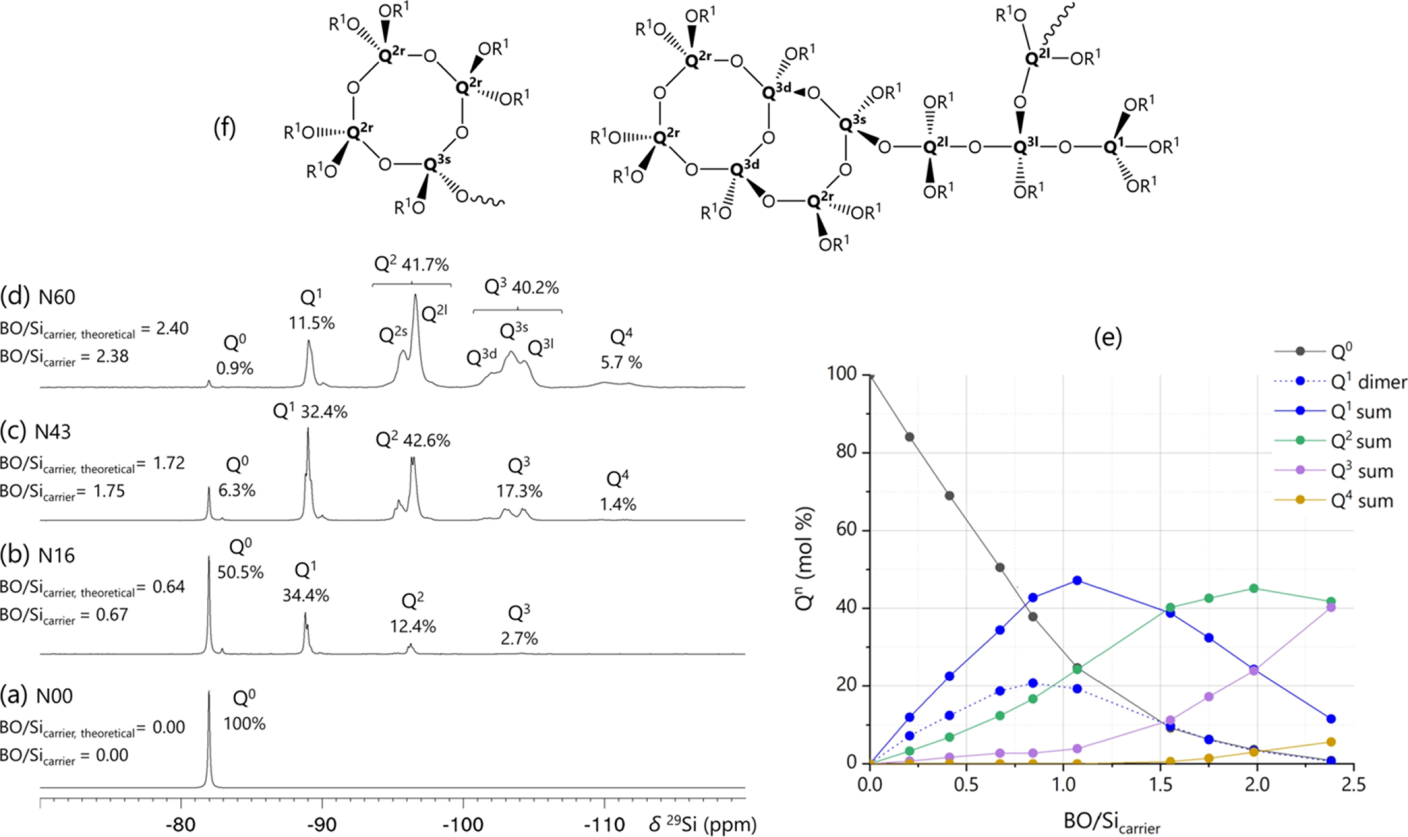
(a–d) ^29^Si NMR spectra showing evolution of Q*^n^* speciation of PEOS carriers as a function of acetic anhydride/TEOS ratio, after completion of the reaction at each addition of acetic anhydride. (e) Relative amounts of Q*^n^* species as a function of BO/Si_carrier_. (f) Chemical structure example for different Q species.

The mechanism of the PEOS substrate synthesis was evaluated by the stepwise addition of acetic anhydride and polymerization of TEOS; aliquots for NMR analysis were collected after the reaction was completed for each step (Figure 2). As expected, first smaller building blocks consisting of Q^1^ dimers, Q^1^ species that are part of various structures, and Q^2^ species are formed followed by the formation of Q species that are part of larger structures with increasing BO/Si_carrier_. In the ^29^Si NMR spectrum of the final PEOS substrate (Figure 2d, N60, Ac_2_O/Si = 1.2, BO/Si_carrier,theoretical_ = 2.4), five different groups of resonances from −82 to −112 ppm assignable to Q^0^ to Q^4^ are observed. Individual Q*^n^* signatures broaden with increasing connectivity and can be further distinguished depending on their specific bonding topology and chemical environment (Table S12). For example, the Q^1^–Q^1^ dimer (i.e., hexaethoxydisiloxane) has a narrow resonance that can still be identified in the much broader signal cluster of Q^1^ species. The separation of the Q^2^ and Q^3^ bands into two (Q^2^) or three (Q^3^) broad signals is more pronounced with a separation of about 2 ppm due to the inclusion in strained rings of four connected silica tetrahedra, i.e., 4 Si and 4 O atoms.^38−40^ Larger rings are far less strained, and their NMR signals cannot be distinguished from linear chain signatures. Three-membered rings are present in minor quantities in silicate glasses and their Q*^n^* signals are further high-frequency shifted than those of four-membered rings (by ca. 6 ppm) due to the much larger ring strain,^41^ but no signals associated with three-membered rings have been detected in PEOS substrate and related funPEOS spectra. Q^2^ in linear and larger ring structures (Q^2l^) displays a signal at −97 ppm, whereas Q^2^ signals belonging to four-membered rings (Q^2s^) appear at −95 ppm. Because of its higher connectivity, Q^3^ can be part of up to 2 four-membered rings, resulting in three peaks of Q^3l^ at −105 ppm (linear/large-ring species that are not part of a four-membered tetrasiloxane ring), Q^3s^ at −103 ppm (part of a single four-membered ring), and Q^3d^ at −101 ppm (part of two four-membered rings). Similar sets of peaks are present for Q^4^, but they are not as clearly resolved.^27,37−40^ The presence of rings makes it impossible to calculate the average *M*_w_, and hence the traditionally defined degree of polymerization from the BO/Si ratio using the Carothers equations.^42^

**Figure 3:**
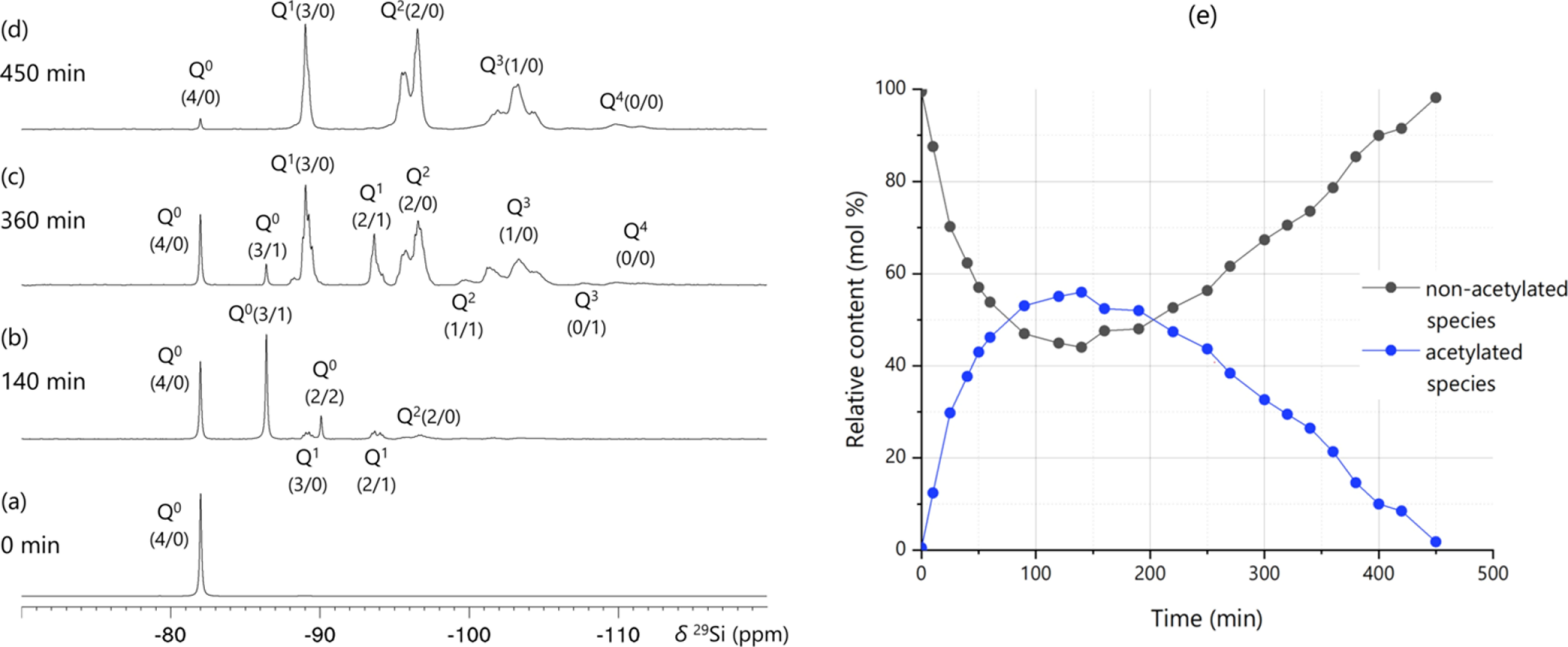
Time-dependent evolution of acetylation and condensation in the production of the PEOS carrier. ^29^Si NMR spectra of (a) pure TEOS, and aliquots of the reaction product with acetic anhydride (Ac_2_O/Si = 1.1), recorded after (b) 140, (c) 360, and (d) 450 min of reaction time at 140 °C. (e) Evolution of the resolved acetylated versus nonacetylated species, acquired by subtraction of the relative content of nonacetylated species from 100%.

It was previously reported that acetoxytriethoxysilane can be isolated.^26,27^ Herein, we elaborated on this topic and carried out a time study to reveal how the acetoxy species form and subsequently further react during the PEOS reaction (^29^Si NMR data in Figures 3 and S22). Mechanistically, the reaction between TEOS and acetic anhydride starts with the formation of acetoxytriethoxysilane, which can react with further acetic anhydride to form diacetoxydiethoxysilane or with TEOS to form a Q^1^–Q^1^ dimer. The acetylation and condensation reactions continue until all acetic anhydride has been consumed, and all acetoxy species have reacted out. Both reactions occur simultaneously, but at different kinetic rates. The condensation of acetoyxtrietoxysilane with alkoxy groups is slower than the acetylation reaction between TEOS and acetic anhydride. As a result, primarily acetylated Q species are formed, which are then reacting by condensation at a slower rate, leading to the asymmetry in the temporal evolution of acetylated versus nonacetylated species (Figure 3e). Note that the acetylated species are not that short-lived under the given reaction conditions and that the reaction can be stopped at any time to isolate them in the final product, if so desired.

The ^29^Si NMR spectra in Figure 3 show the evolution of the silica speciation during PEOS carrier synthesis carried out at 140 °C as a function of reaction time, i.e., spectra from aliquots collected at different times. The signals are labeled as *Q^n^*(*l*/*m*) following the notation of Jaumann et al.,^26^ where *n* corresponds to the number of bridging oxygens, *l* the number of alkoxy groups, and *m* describes the number of acetoxy groups attached to the observed Si atom. The signals for acetylated intermediates shift by ca. 4.4 ppm to lower frequencies, i.e., to the right, with every additional acetoxy group. For example, the acetylation of TEOS leads to a decrease in the intensity of the Q^0^(4/0) signal near −82 ppm and the appearance and growth of the Q^0^(3/1) signal near −86 ppm (Figure 3b). Further acetylation leads to the emergence of a Q^0^(2/2) signal near −90 ppm. The situation for Q1 is similar, with Q^1^(3/0) and Q^1^(2/1) signals near −89 and −94 ppm, respectively (Figure 3c). For more polymerized and/or more highly acetylated species, there is often a (partial) overlap with other Q signals:^26^ a Q*^n^* signal with 2 acetoxy groups is often hard to distinguish from the Q^*n*+1^ signal without acetoxy groups, e.g., Q^1^(1/2) and Q^2^(2/0) (Figure 3c). This adds significant uncertainty to the quantification, but the absolute uncertainty is limited due to the low abundance of double-acetylated species (Table S13).

### 3.2 funPEOS Synthesis

#### 3.2.1 Confirmation of Q–T Connections

**Figure 4:**
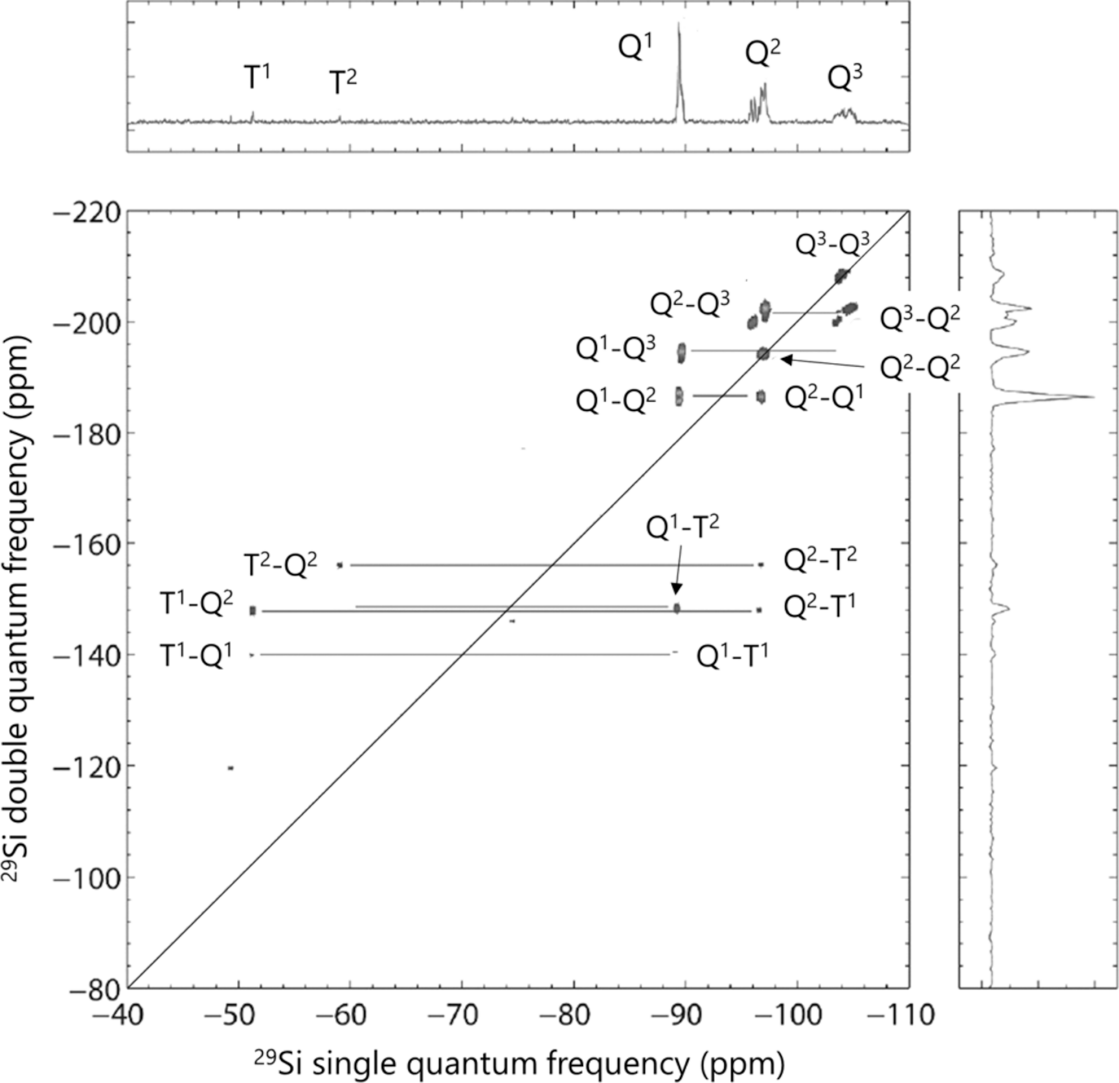
^29^Si–^29^Si INADEQUATE NMR spectrum and its projections of a funPEOS with a TEOS-based carrier and an MTES-based shell (DF = 0.38, BO/Si_carrier_ = 2.33, BO/Si_shell_ = 2.08), the synthetic protocol is described in the Supporting Information in Section S1.2.2.

The architecture of the funPEOS product is designed prior to synthesis by selecting the desired BO/Si_carrier,theoretical_, and the type and amount of functional silane (*n*_shell_/*n*_carrier_) to tailor the properties of the product. Conveniently, the ^29^Si NMR signals of the species derived from the functional trialkoxysilanes (T) are separated well from those of the carrier (Q), except in the case of vinyl- (Figure 1b) or phenyl-functionalization. To investigate the connectivity of individual species in a funPEOS (i.e., Q–Q, Q–T, and T–T bonding), a 2D INADEQUATE ^29^Si–^29^Si NMR spectrum was recorded from the PEOS carrier with a grafted methyltriethoxysilane (MTES) shell (Figure 4). Peaks located on the tilted diagonal axis display neighboring ^29^Si atoms (connected through a siloxane bond) of the same species (e.g., Q^2^–Q^2^ and Q^3^–Q^3^). Adjacent Q–T species show up as a pair of correlations in each cross section with the midpoint on the tilted diagonal axis (e.g., T^2^–Q^2^ and Q^2^–T^2^). Figure 4 shows well-resolved signals of covalent Si–O–Si bridges between Q–Q and Q–T species, which indicates that the functional triethoxysilanes react with the presynthesized PEOS carrier through Q–T heterocondensation. The most pronounced pairs of signals are those related to T^1^–Q^2^, resulting from the grafting of functional silane monomer T^0^ on a terminal Q^1^ species of the substrate, and T^2^–Q^2^ connections, derived, for example, from the additional grafting of functional silane on an already grafted T^1^. A low-intensity pair of signals is present for T^1^–Q^1^ connections, i.e., the dimer formed between MTES and TEOS. Note that in some cases, e.g., Q^1^–Q^3^, only one signal is present rather than a pair, due to the low sensitivity of the INADEQUATE experiment. The signals related to T–T pairs are below the detection limit; hence, the occurrence of T–T homocondensation into separate trialkoxysilane-based oligomers cannot be observed in this measurement, but their presence cannot be excluded given the low sensitivity of the INADEQUATE experiment.

#### 3.2.2 Effect of Functional Group Size on Grafting Efficiency

We observed that obtaining funPEOS product with well-grafted functional silane, with BO/Si_shell_ > 1.0 and low residual functional silane monomer (T^0^) using the same reaction protocol described in the Supporting Information in Section S6.2, was more challenging for large functional groups, e.g., for octyl versus propyl versus methyl (Figure S23). Thus, grafting silanes with bulkier functional groups requires more intense reaction conditions to reach the same grafting efficiency. The reaction can be optimized by tuning various parameters, including a higher acetic anhydride addition, a higher concentration of catalyst, or an increased reaction temperature and time. The nature of the alkoxy groups also plays a role in the final conversion: selecting methoxy-terminated functional silanes leads to better conversion and a higher BO/Si_shell_ (Figure S25) and is particularly recommended for functional silanes with sterically more demanding functional groups such as octyl or isobutyl (further discussion presented in the Supporting Information in Section S6.3).

#### 3.2.3 Methoxy–Ethoxy Exchange

**Figure 5:**
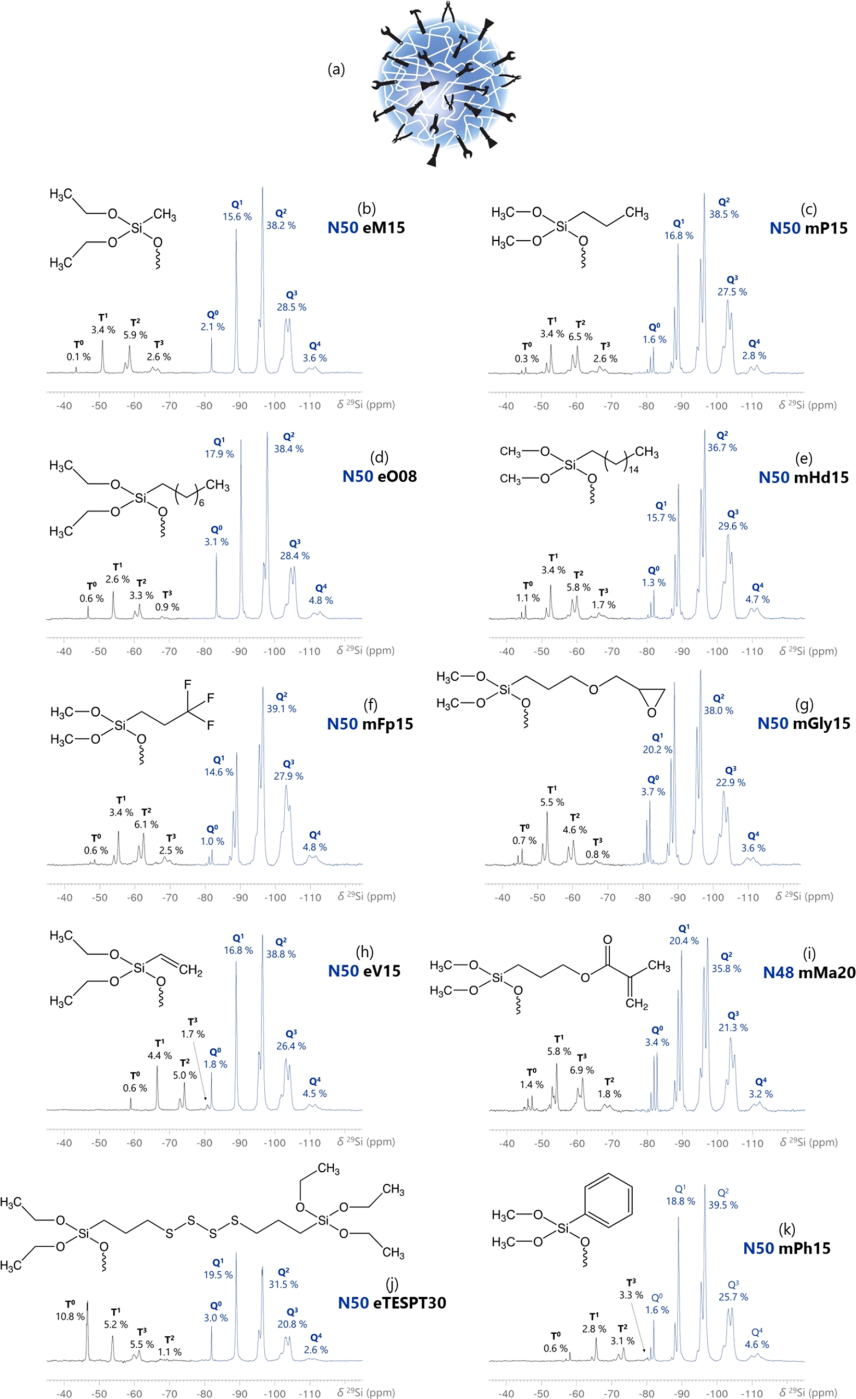
^29^Si NMR spectra obtained from synthesized funPEOS molecules acquired by grafting organofunctional trialkoxysilanes R′-Si(OR)_3_ onto PEOS carrier (a). The molecular structures correspond to a grafted T^1^ species with the following functional groups: (b) methyl (M), (c) propyl (P), (d) octyl (O), (e) hexadecyl (Hd), (f) fluoropropyl (Fp), (g) glycidoxypropyl (Gly), (h) vinyl (V), (i) propylmethacrylate (Ma), (j) propyltetrasulfide (TESPT), and (k) phenyl (Ph).

The use of functional methoxy-silanes brings advantages, such as faster conversion described above, and often lower price compared with their ethoxy analogues, and are therefore often preferred in the preparation of funPEOS. Mixing functional methoxy-silanes and PEOS silanes bearing ethoxy groups in the presence of TTIP causes transesterification of silicon alkoxy moieties.^38,43−45^ The populations of ethoxy and methoxy groups can be monitored by clearly distinguishable ^29^Si NMR resonances of both the Q and T chemical species, particularly for those with lower connectivity, i.e., T^0^ and Q^0^ (Figures 5b,d–f,h,j, and S24). For example, the T^0^ moiety in a funPEOS produced from a PEOS substrate and hexadecyltrimethoxysilane exhibits all four expected ^29^Si resonances separated by ca. 1.1 ppm assigned to species with zero ethoxy (−42.1 ppm), one ethoxy (−43.2 ppm), two ethoxy (−44.4 ppm), and three ethoxy groups (−45.5 ppm) (Figure S24). Under most synthesis conditions, the methoxy/ethoxy ratio of the T and Q species corresponds to the molar ratio of the alkoxy groups present in the starting material (Figure 5b,d–f,h,j), at least for the Q^0^ and T^0^ species for which this can be verified, but this is not always the case (Figure S24a).

#### 3.2.4 Scale-up and Mass Balance

To demonstrate scalability, the synthesis was scaled up to a 4 kg batch size in a 7 L reactor vessel (Figures S5 and S6). The mass balance of both laboratory and scale-up experiments has been verified (Supporting Information, Section S6.4) and indicates full stoichiometric control for funPEOS prepared with functional silanes with high boiling points. However, for more volatile functional silanes, e.g., MTES with its boiling point of 142 °C, some of the functional silanes may boil over together with the (m)ethyl acetate side product. These issues with substoichiometric conversion due to evaporation can be easily resolved by adjusting the synthesis temperature according to the individual reactants or by adjusting the reaction setup, e.g., the addition of a reflux condenser with regulated temperature (above the boiling point of ethyl acetate side product but below the boiling point of organofunctional silane) between the reaction vessel and distillation bridge.

#### 3.2.5 Library of funPEOS

The greatest strength of funPEOS technology is the ability to graft a wide variety of different functional T-type, D-type, and/or M-type silanes and modify the composition to match a targeted application. A selection of prepared funPEOS with the corresponding functional groups and ^29^Si NMR spectra are presented in Figure 5. All of the funPEOS compounds that have been produced in this study with their BO/Si values and the variation of *n*_shell_/*n*_carrier_ ratios are summarized in Table 1. Additional ^1^H, ^13^C NMR, and Fourier transform infrared (FTIR) spectra of the various funPEOS compounds, a standard PEOS substrate, as well as the respective functional silane monomers are shown in the Supporting Information (Figures S26–S53). The NMR and FTIR spectroscopic data confirm the retention of various functional groups. One exception is funPEOS with glycidyl-functional silane: although no obvious signals of ring-opened species were found in the spectrum (expected in the range around 3.8–4.7 ppm), the ^1^H NMR intensity ratios (protons 7′ and 7″ versus protons 2, Figure S34) indicate ring-opening side reactions occurred up to 30%. The reaction between epoxide and anhydride involves the anhydride reacting with a hydroxyl group,^46^ therefore converting it into an intermediate monoester and carboxylic acid, which can subsequently attack the epoxide carbon.^47,48^ Without a catalyst like alcohols or tertiary amines, this reaction requires high temperatures,^49^ so it may be possible to avoid the ring opening by proper choice of temperature.

**Table 1:** Synthesized funPEOSs and their Specifications: Functionality Introduced into funPEOS, Chemical Name of Functional Silane Monomers, Abbreviations Used in funPEOS Nomenclature, Theoretical and Experimentally Determined Average Number of Bridging Oxygens per Si Atom in Carrier (BO/Si_c,t_, BO/Si_c,NMR_), Average Number of Bridging Oxygens per Si Atom Inshell (BO/Si_s,t_, BO/Si_s,NMR_,), Molar Ratio of Shell Silane to Carrier (*n*_s_*/n*_c_), and Number of Synthesized Batches (*x*)

functional silane			specific example				tested ranges			
functionality (R′)	reagent	abbr. used in funPEOS	BO/Si_c,t_	BO/Si_c,NMR_	BO/Si_s,NMR_	*n*_s_/*n*_c_	*x*	BO/Si_c,t_	BO/Si_s,t_	*n*_s_/*n*_c_
methyl	methyltriethoxysilane	eM	2.0	2.18	1.86	0.15	74	1.9–2.3	1.6–2.1	0.10–0.60
propyl	propyltriethoxysilane	eP	2.0	2.21	1.55	0.15	15	2.1–2.3	0.4–1.6	0.08–0.30
propyl	propyltrimethoxysilane	mP	2.0	2.18	1.54	0.15	28	2.1–2.5	1.0–2.1	0.10–0.30
octyl	octyltriethoxysilane	eO	2.0	2.17	1.70	0.15	130	2.0–2.5	0.2–2.0	0.08–0.30
hexadecyl	hexadecyltrimethoxysilane	mHd	2.0	2.24	1.71	0.15	21	1.9–2.3	0.9–1.8	0.08–0.15
trifluoropropyl	3,3,3-trifluoropropyl-trimethoxysilane	mFp	2.0	2.21	1.78	0.15	9	2.0–2.3	1.3–1.8	0.01–0.15
glycidoxypropyl	(3-glycidoxypropyl) trimethoxysilane	mGly	2.0	2.00	1.36	0.15	82	1.6–2.2	0.2–1.7	0.05–0.30
vinyl	vinyltriethoxysilane	eV	2.0	2.17	1.66	0.15	50	2.0–2.3	1.0–1.9	0.08–0.30
propylmethacrylate	3-(trimethoxysilyl) propylmethacrylate	mMa	1.9	2.25	1.56	0.30	74	1.8–2.3	0.7–1.7	0.15–0.30
propyltetrasulfide	bis[3-(triethoxysilyl) propyl] tetrasulfide	eTESPT	2.0	2.11	1.14	0.15	123	2.0–2.2	0.1–1.3	0.20–0.60
phenyl	phenyltrimethoxysilane	mPh	2	2.14	1.93	0.15	3	2.14	1.93	0.15
dimethylsilyl	dimethyldiethoxysilane	eD	2.0	2.18	1.11	0.15	1	2.18	1.11	0.15
trimethylsilyl	hexamethyldisiloxane	TMS	2.2	2.37	1.00	0.15	4	2.0–2.5	0.9–1.0	0.15–0.50

Functional groups such as different-length alkyl chains, trimethylsilyl, and fluoropropyl are known for their hydrophobic character; by changing the ratio of *n*_shell_/*n*_carrier_, the hydrophobicity of the molecular building block can be adjusted. Groups such as vinyl, methacrylate, and glycidoxy provide reactive sites for further chemical modification or cross-linking. Different functionalizations can be mixed together or grafted sequentially to create substrate-double-shell funPEOS. In the case of funPEOS with more than one functionality, the order of addition can be varied. Typically, it is beneficial to add smaller functional groups first to increase the grafting efficiency, e.g., first an inert short-chain hydrophobic group and then a cross-linking group; first a reactive group and then a long-chain hydrophobic group; or first a long-chain alkyl group, before a bulky trimethylsilane is added. Like that, the molecular building block can be bottom-up tailored to fit the requirements for various applications. Moreover, chemical postmodification of shell functionality can be carried out to expand the already broad variety of functional groups.

#### 3.2.6 Molecular Weight

**Figure 6:**
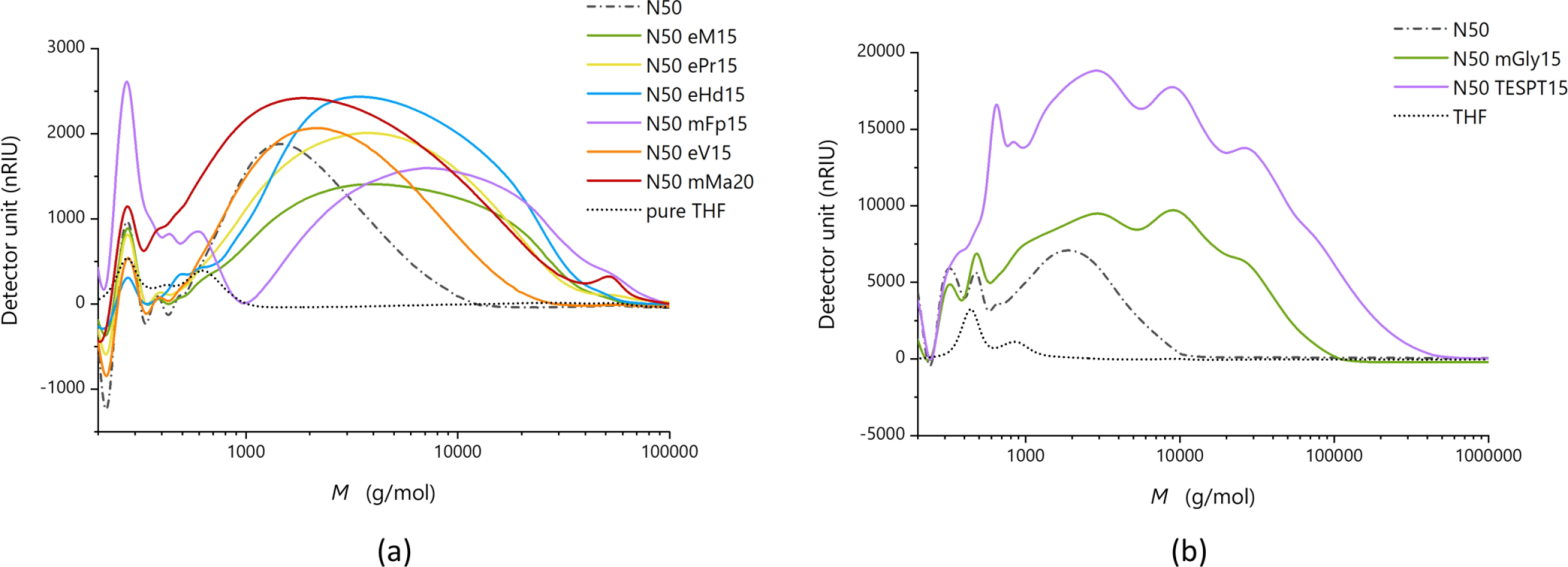
GPC chromatograms for columns with (a) a narrower *M*_w_ range (500–60,000 g/mol) and (b) a wider *M*_w_ range (200–2,000,000 g/mol). Peaks in the low *M*_w_ region come from the elution solvent THF (dotted line). For the same sample (N50), the different columns provide qualitatively similar chromatograms, although there is a small shift and hence somewhat different *M*_w_, *M_n_*, and PDI values (Table 2).

Various funPEOS compounds were analyzed by GPC (gel permeation chromatography; Figures S54 and S55). The distribution of molecular weights (Figure 6) supports the substrate grafting argument since all of the observed funPEOS samples have higher molecular weights than the reference PEOS substrate from which they are derived. The PEOS carrier (N50, Ac_2_O/Si = 1, BO/Si_carrier,theoretical_ = 2.0) shows the narrowest *M*_w_ distribution with a polydispersity index (PDI) of 1.53 and the lowest molecular weight (Table 2), which is similar to those reported in the work of Zhu and co-workers.^27^ Hydrophobic funPEOS containing methyl (M), propyl (P), hexadecyl (Hd), fluoropropyl (Fp), or vinyl (V) functional groups display a single broad peak in their distribution (Figure 6a) with a PDI around 2. Samples containing functional groups more prone to self-polymerization, e.g., TESPT, glycidoxy (Gly), or methacrylate (Ma), show a broader *M*_w_ distribution (PDI values around 3 or greater) at much higher molecular weights up to 100s of kDa (Figure 6b). This may indicate that some of these compounds undergo self-polymerization over time through functional group cross-linking. The relatively strong signal within the lower M range (<1000 g/mol) may indicate the presence of some small oligomers of the methacrylate-silane, but this observation has not been investigated in more detail. Compared with the group of hydrophobic compounds, which are very stable in solution, the funPEOS containing Ma and TESPT, for example, tend to gel when improperly stored. Note that the GPC data were obtained from funPEOSs that were prepared using different Ac_2_O/Si ratio (molar ratio between acetic anhydride and organofunctional silane) values and that the final *M*_w_ is highly dependent on this ratio.

**Table 2:** Number Average Molecular Weight *M̅*_*n*_, Weight Average Molecular Weight *M̅*_w_, and Polydispersity Index (PDI) of funPEOS Compounds Measured by GPC

compound	*M̅*_*n*_ (g/mol)	*M̅*_w_ (g/mol)	PDI
N50	1497	2214	1.48
N50^a^	952^a^	1967^a^	2.13^a^
N50 eM15	2671	7008	2.62
N50 eP15	2316	5656	2.44
N50 eHd15	2601	6714	2.58
N50 mFp15	4996	11,393	2.28
N50 mGly15^a^	1735^a^	9452^a^	5.45^a^
N50 eV15	1856	3508	1.89
N48 mMa20	1494	4916	3.29
N50 eTESPT30^a^	2068^a^	20,005^a^	9.67^a^

a Values obtained using GPC column with range 200–2,000,000 g/mol. Other values measured with a 500–60,000 g/mol column.

### 3.3 Mixed Functionalization

**Figure 7:**
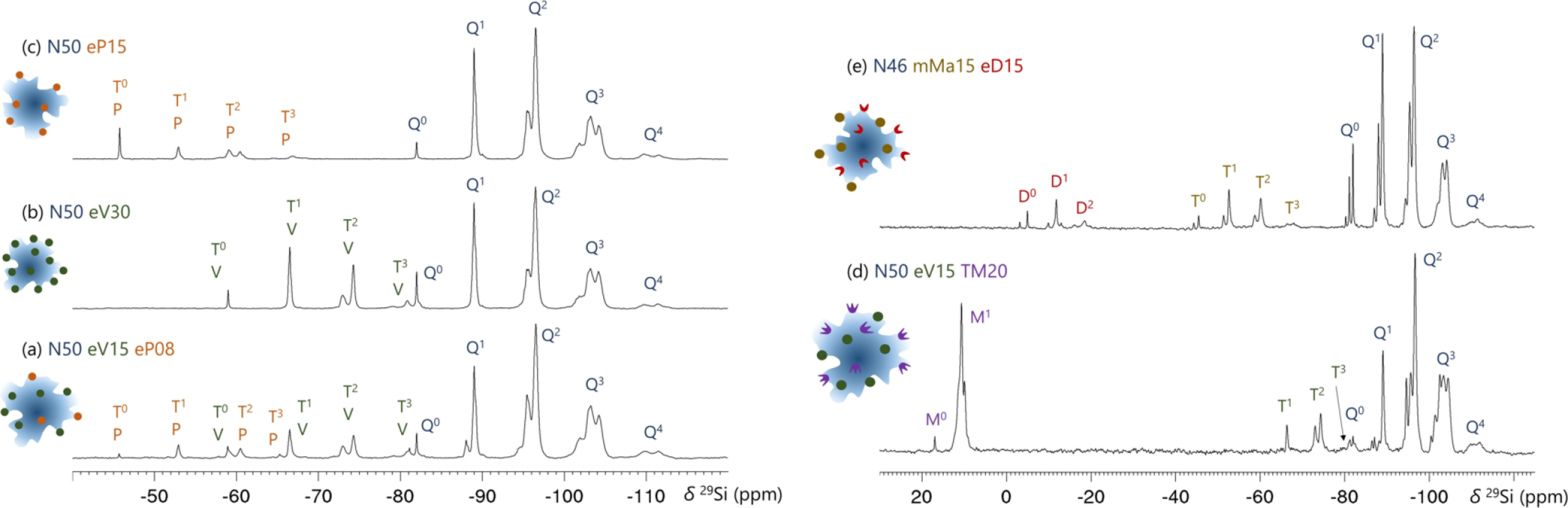
^29^Si NMR spectra of (a) double-functionalized funPEOS containing vinyl and propyl functional groups compared with spectra of monofunctionalized PEOS with only (b) vinyl and (c) propyl groups; double-functionalized funPEOS with (d) vinyl and trimethylsilyl and (e) dimethylsilyl and propylmethacrylate functional groups.

When considering the potential use of such polymeric liquids in coatings, polymer systems, paints, dispersions, or as interface active compounds, it is often best to combine different functionalities in a single building block. For example, it may be beneficial to combine tailored hydrophobicity with specific reactive groups. Incorporating hydrophobic functionalities through alkyl-based T, D, or M silanes or fluoropropyl T silane allows controlling the steric accessibility and hydrophobic features of a material, which tailors its application-relevant properties such as solubility and compatibility with solvents, polymers, and inorganic and hybrid phases. Modifying the PEOS carrier with specific functionality such as epoxy, vinyl, methacrylate, phenyl, or tetrasulfide groups opens up further possibilities for chemical postprocessing steps. The simultaneous reaction of Q-type carriers with two different types of T-silanes leads to double-functionalized PEOS products. Figure 7a–c shows ^29^Si NMR data of an example of a PEOS carrier modified with PTES to adjust the hydrophobic properties and with VTES to introduce radically polymerizable groups. Another example shows the introduction of hydrophobicity by dimethylsilyl or trimethylsilyl groups, while the reactive functionality comes from methacrylate or vinyl groups (Figure 7d,e). The broad diversity of this technology allows the exploration of different compositions that can be combined depending on the desired application.

### 3.4 Secondary Modification of funPEOS

**Figure 8:**
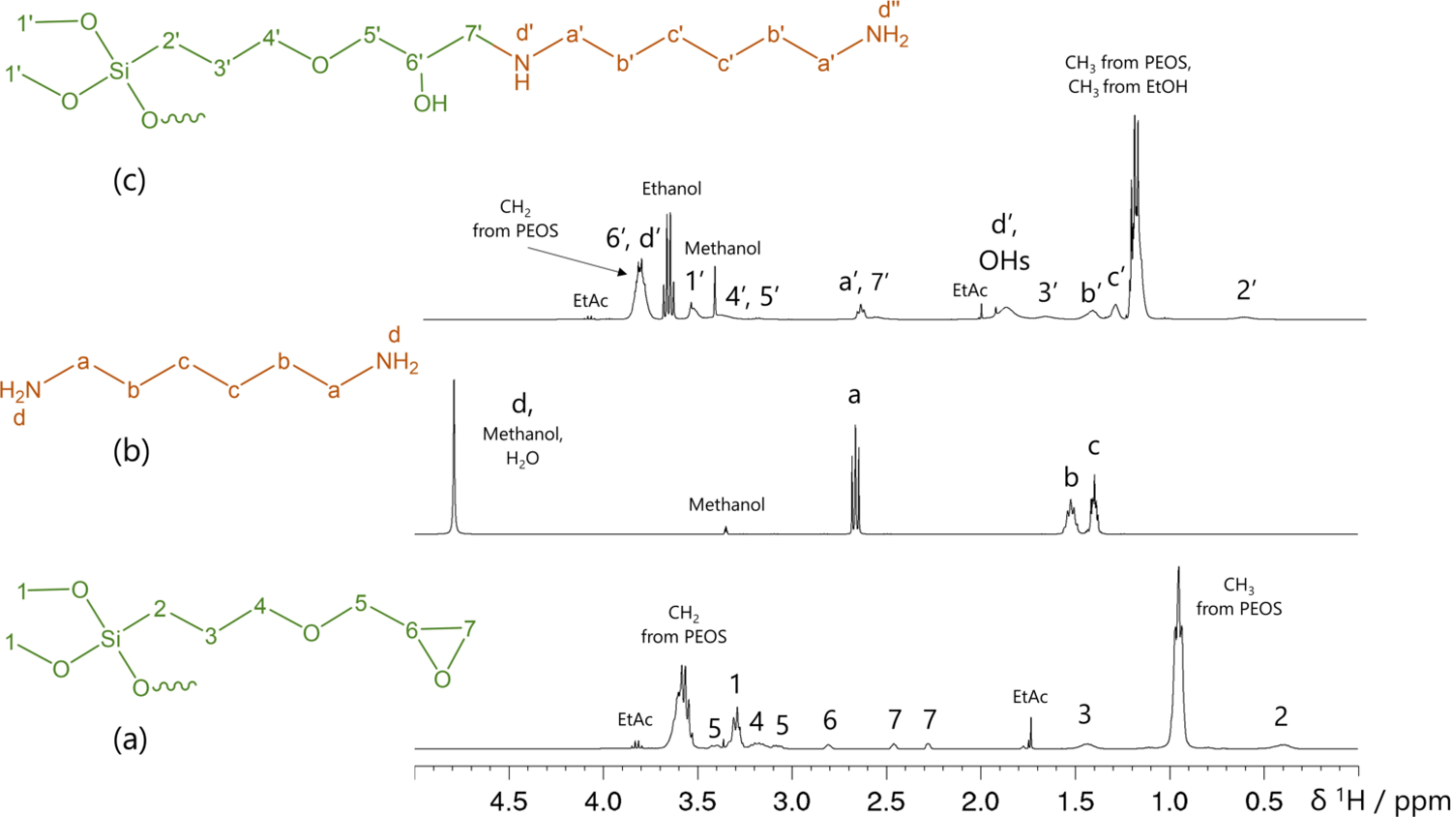
^1^H NMR spectra from (a) single shell N50 mGly15 in CDCl_3_, (b) HMDA starting material in methanol-D4, and (c) postmodified N50 mGly15 with HMDA in CDCl_3_ in 1:1 stoichiometry (nominal) of epoxide to diamine (*n*_shell_/*n*_HMDA_ = 1) mixed under ambient conditions.

The limitations of the presented technology include the accessibility of commercially available functional silanes as well as the necessity of selecting functional silanes that have no cross-reactivity with acetic anhydride, ethyl acetate, or other functionalities in the case of multifunctionalized carrier-shell structures. For example, acetic anhydride and amino groups readily react to form amides,^50,51^ and therefore aminosilanes are a nontrivial grafting system for the funPEOS synthesis. Fortunately, amino groups and other functionalities can be introduced through postmodification reactions on the T-type organofunctional substituent using addition or substitution chemistry, which enables the production of a wide range of compounds. In particular, silanes with epoxy, glycidoxy, or vinyl groups that are compatible with the funPEOS production process provide anchoring sites for further organic modifications. An important example is the addition of different amines to a glycidoxy-bearing funPEOS by means of an amine–epoxy ring-opening reaction (Figure S56), including mono and diamines with different chain lengths under defined conditions.^52−54^Figure 8 shows ^1^H NMR spectra recorded from postmodified N50 mGly15 with hexamethylenediamine (HMDA) prepared by mixing under ambient conditions in a 1:1 molar ratio of *n*_shell_/*n*_HMDA_. The data confirm the reaction between epoxide and diamine: in the N50 mGly15 precursor (Figure 8a), the resonances at 2.81 ppm (H-6) and the two signals at 2.46 and 2.28 ppm (H-7) show the typical epoxide ABX-type pattern. In the ^1^H NMR spectrum of the reaction product (Figure 8c), the resonances of H-7′ are now magnetically equivalent, and H-6′ is significantly shifted toward higher frequency. This reaction was also confirmed in a monomeric model system (Figures S57 and S58).

Postmodification is a simple strategy to introduce amino or other functional groups into funPEOS compounds. This way, the range of functional groups for this type of funPEOS formulation can be extended considerably beyond the spectrum of compounds compatible with anhydride-based condensation chemistry.

### 3.5 Applications

The near-infinite array of possibilities to produce different types of funPEOS molecular liquids opens up numerous potential applications, many of which are currently under investigation. In this study, we limit ourselves to three simple proof-of-concept applications. A variety of other applications in the coatings, adhesives, and polymers field are being pursued by Empa and its spin-off company Siloxene AG (confidential, not shown here).

**Figure 9:**
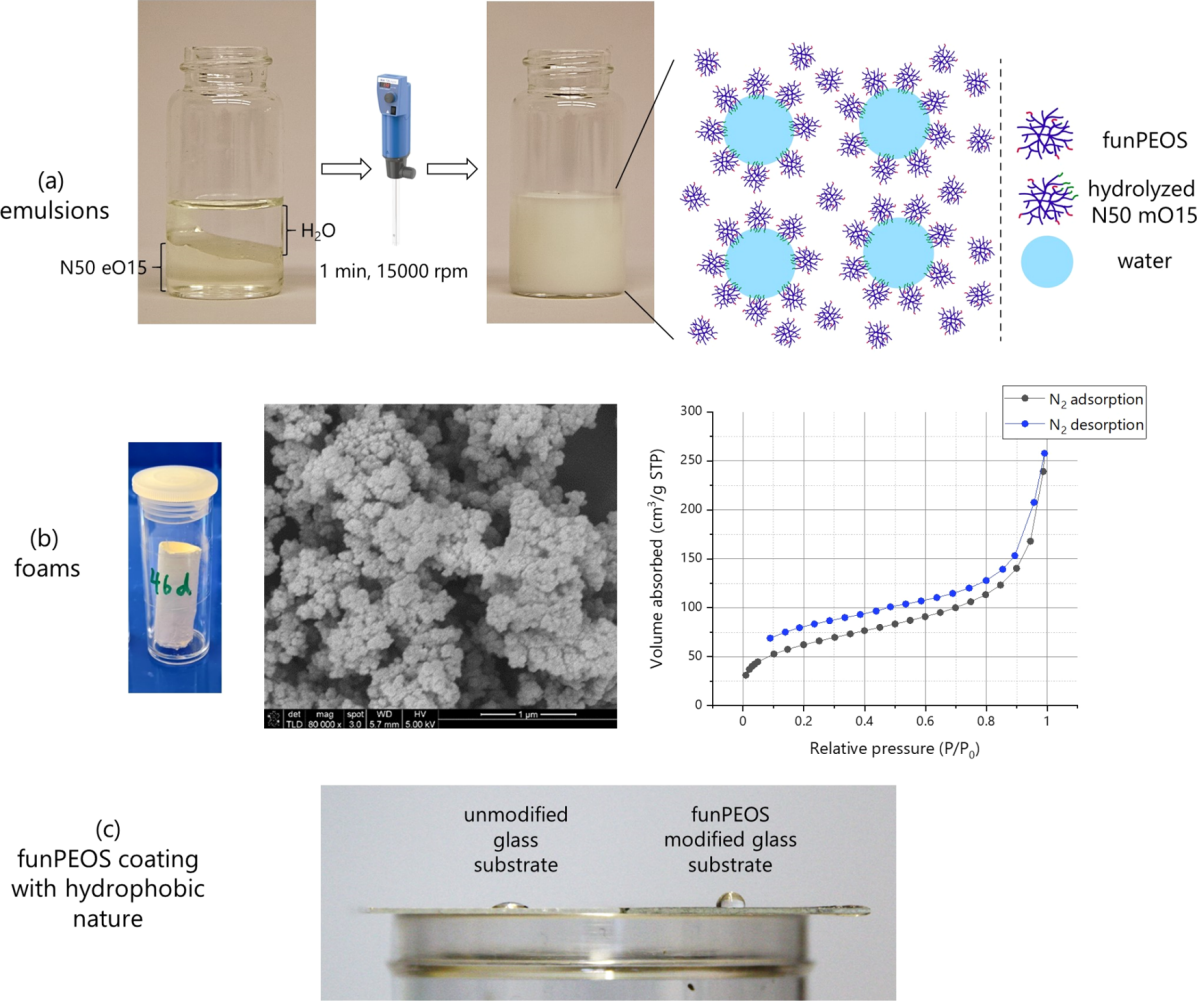
Proof-of-concept applications of funPEOS in (a) water–oil emulsions, (b) surfactant-free foams with SEM image and typical N_2_ BET adsorption–desorption isotherms (see SI, Section S9), and (c) increase in water contact angle after application of a funPEOS coating.

The hydrophobic nature of alkyl-functionalized hyperbranched PEOS proved to be very beneficial in emulsion preparation.^55^ Here, we report an approach to produce surfactant-free stable aqueous emulations using funPEOS functionalized with hydrophobic alkyl groups such as octyl or hexadecyl. Emulsions were successfully prepared via both water-in-oil and oil-in-water approaches by simply combining water and funPEOS (N50 eO15 or N50 mHd15) in 1:2 and 2:1 (example with N50 eO15 presented in Figure 9a) volume ratios, respectively. All of the resulting emulsions are homogeneous, and the emulsions produced with hexadecyl-functionalized PEOS showed higher viscosity than those with octyl-functionalized PEOS. Once formed, the emulsions are stable without mechanical agitation for at least 14 days without setting or precipitation.

Silica gels and their dried products (aerogels and xerogels) prepared by hydrolysis–condensation processes are well documented.^56−58^ Typically, these structures comprise an open-porous network of interconnected amorphous silica, whose pore size can be adjusted by the synthesis conditions. Classical silica aerogels exhibit extraordinary properties such as high specific surface area (250–800 m^2^ g^–1^), high pore volume (>90%), low envelope densities (0.003–0.3 g cm^3^), and low thermal conductivity (∼0.015 W m^–1^ K^–1^).^59^ Another example is flexible silica aerogel, better described as a silica foam. These materials lack mesoporosity and exhibit poorer surface area and thermal conductivity but have much better mechanical properties compared with standard silica aerogels.^60^ funPEOS can be incorporated into the structure of such materials to improve control over pore structure, with additional benefits such as surfactant-free synthesis and faster gelation time. Figure 9b shows a silica foam prepared from three monomers (DMDES, MTES, and VTES) and two funPEOS (N60 eM15 and N45 eV30) (see Section S9 in the Supporting Information). The foam exhibits a Brunauer–Emmett–Teller (BET) surface area of 221 m^2^/g (Figure 9b right part) and the scanning electron microscope (SEM) image (Figure 9b middle part) indicates that the microstructure is similar to a mesoporous aerogel. Another route to fabricate light porous materials where the use of funPEOS can be beneficial is high internal phase emulsion (HIPE) templating.^61^ Different foams with well-defined pore structures can be obtained via this method without the use of supercritical drying. Here, funPEOS can serve as a silica precursor as well as an emulsion stabilizer.^62^

Another possible application is the production of scratch-resistant coatings with hydrophobic behavior, which are usually produced by sol–gel processes. To demonstrate this, funPEOS bearing various hydrophobic groups (i.e., methyl, propyl, octyl, hexadecyl, or fluoropropyl) were mixed with a mixture of isopropanol and 1 molar HCl in water to promote hydrolysis followed by the addition of an 8 molar ammonia solution to trigger condensation. Self-curing films were prepared from the corresponding alcohols using an automated dip-coater setup. Contact angles of >90° (Figure 9c) were achieved for all compositions studied. As reported before, solvent-based sol–gel coatings have shown outstanding corrosion protection performance on metals.^1,63−76^ However, these coatings have several drawbacks: films are brittle and prone to cracking, and high temperatures are usually required for curing.^1^ Coatings based on funPEOS-based sols can circumvent some of these disadvantages and are a promising alternative to traditionally prepared sol–gel coatings.

## 4 Conclusions

This study elaborates on a new type of hybrid polysiloxane precursor chemistry. For the first time, a selective grafting protocol is shown, allowing a hyperbranched polyalkoxysilane substrate to be decorated with a “shell” comprised of organofunctional alkoxysilanes using nonhydrolytic acetic anhydride condensation chemistry. The described methodology therefore opens up a technological route to liquid functional molecular building blocks with a morphology of a functional silane-decorated siloxane carrier and nearly unprecedented freedom for material design at the molecular level. The presented approach for funPEOS involves a two-step process in which first a PEOS carrier is prepared by nonhydrolytic condensation chemistry, followed by the grafting of organofunctional alkoxysilanes at the carrier’s perimeter. The temporally separated shell deposition can be repeated, allowing for the assembly of complex multilayer shell architectures. Conveniently, the chemical identity of funPEOS molecules consisting of Q-type polyalkoxysilane species and T, D, or M organofunctional moieties can be classified by ^29^Si NMR to verify the composition of the synthesized products. The diversity of the funPEOS technology is a great asset for modern materials by design concepts. Exploring all of the different functionalities and potential applications is a formidable task, but the versatility of the approach offers countless possibilities for applications in everyday life.

## 5 Experimental Section

### 5.1 PEOS/funPEOS Synthesis Protocol

To prepare a PEOS substrate, 100 g (481 mmol) of TEOS (Q-type silane) was mixed with 0.33 mL (1.1 mmol) of TTIP catalyst. The reaction mixture was heated to 140 °C in a round-bottom flask in a water-free, inert atmosphere (N_2_) under stirring (Figures S3 and S4). Acetic anhydride was gradually added up to a 1:1 molar ratio (49.1 g, 481 mmol) using a peristaltic pump with the speed of addition of 0.8 mL/min. The reaction side product, i.e., ethyl acetate, was distilled off until the flow of the distillate stopped, which indicated the end of the reaction after 300 min. A pale yellow, oily PEOS liquid was obtained in the amount of 66.4 g together with 79.8 g of condensate. The temporally separated addition of organofunctional silanes enables the selective buildup of a functional T-type siloxane shell onto the already produced Q-type substrate. In this step of the one-pot synthesis, 14.9 g (72.1 mmol) of PTES (T-type silane) and 0.19 mL (0.61 mmol) of TTIP were added at once to the round-bottom flask with 66.4 g of the obtained carrier under continuous stirring at 140 °C. In addition to this, a second stoichiometric amount of acetic anhydride (AA) in a PTES/AA molar ratio of 1:1.25 (9.2 g, 90.1 mmol) was introduced into the reaction mixture by syringe pump with the speed of addition of 0.4 mL/min. While keeping the reaction temperature constant at 140 °C throughout the whole synthesis, further ethyl acetate was formed and distilled off. The reaction was followed by monitoring the formation of the condensate. Once the distillation stopped, the rest of the low-molecular reaction products and residual starting materials in the reaction mixture were removed by vacuum distillation through gradually lowering the pressure inside the reaction vessel and holding the final pressure of 110 mbar at 140 °C for 15 min. The shell buildup step took 120 min, which together with the first step yields 420 min. 97.6 g of condensate and 69.5 g of a yellow, stable, liquid funPEOS product soluble in most organic solvents were obtained (Figure S8, N50 eP15).

A general synthesis protocol that can be used to prepare funPEOS products with different parameters is presented in Sections S1.2 and S3.4 of the Supporting Information.

### 5.2 Characterization

PEOS and funPEOS were characterized by NMR, FTIR, and GPC.

NMR spectra were acquired on a Bruker Avance III HD NMR spectrometer equipped with a 9.4 T wide-bore magnet, corresponding to Larmor frequencies of 400.2, 100.6, and 79.5 MHz for ^1^H,^13^C, and ^29^Si, respectively. Each ^29^Si NMR sample was prepared by mixing a 0.1 molar stock solution of chromium(III) acetylacetonate (Cr(acac)_3_, Sigma-Aldrich) as a relaxation agent in chloroform-d (CDCl_3_, 99.8% + Ag, Deutero) and given PEOS/funPEOS sample in a 15:85 volume ratio resulting in a 15 mM Cr^3+^ concentration. ^29^Si single-pulse NMR spectra were collected using a 5 mm CryoProbe Prodigy probe without a lock (no stable lock on ^2^H owing to the low CDCl_3_ concentration and the presence of the relaxation agent) under WALTZ16 proton decoupling during data acquisition. Typically, 1024 scans were collected with a pulse length of 3.5 μs (π/6 pulse) and a recycle time of 3.8 s. Under these conditions, no change in absolute or relative peak intensities could be observed for longer recycle delays (Figure S9 and Table S6). For representative samples, T_1_ relaxation times of ∼50 s (no relaxation reagent), 3–4 s (5 mM Cr^3+^), and 1.5 s (15 mM Cr^3+^) have been determined, ensuring a quantitative recording of ^29^Si NMR data of samples prepared as described above. The ^29^Si NMR chemical shifts are referenced to the resonance of TEOS (Q^0^) at −82.0 ppm^38,77^ as an internal standard, corresponding to the signal of the standard reference substance tetramethylsilane at 0.0 ppm. Residual TEOS was present in most of the samples.

Samples for ^1^H and ^13^C NMR measurements were prepared by mixing PEOS or funPEOS samples with CDCl_3_ in a volume ratio of 1:12. ^1^H and ^13^C single-pulse NMR spectra were recorded with a lock on CDCl_3_. Typically, 16 (^1^H) and 128 scans (^13^C) were collected. The ^1^H and ^13^C NMR chemical shifts were referenced to the remaining resonances of chloroform-D at 7.28 and 77.0 ppm, respectively.

Connectivity between different Si species was investigated by a gradient-selected version of the ^29^Si–^29^Si NMR INADEQUATE pulse sequence (inadgpqfsp in Bruker library) applying an adiabatic refocusing pulse^78^ with selection of a 10 Hz J-coupling constants along the Si–O–Si bonds. The 2D NMR spectrum was collected on a sample with natural ^29^Si abundance (4.7%) applying 5 mM of Cr(acac)_3_ relaxation agent to shorten recycle times (6.5 s), as described above, applying 512 scans, and 96 increments in the indirect dimension were recorded. Although most of the ^29^Si–^29^Si J-couplings are in the range of 2–4 Hz,^79^ the chosen 10 Hz coupling turned out to be more effective because the waiting times in this pulse sequence are much shorter and the magnetization relaxes less during the polarization transfer. Owing to the inherent low sensitivity of the experiment, data acquisition took almost 4 days, and only one spectrum of a single sample was recorded.

Fourier transform infrared spectroscopy (FTIR, Bruker Tensor 27; OPUS Version 8.2 software) was carried out in the attenuated total reflectance (ATR) mode.

The molecular weights were analyzed using gel permeation chromatography (GPC) (Agilent Technologies 1260 Infinity) with a refractive index (RI) detector. Two different columns were used for the narrower (Agilent, PLgel 5 μm; 500–60,000 g/mol) and the wider (Agilent, PLgel 5 μm mixed C; 200–2,000,000 g/mol) molecular weight distributions. The eluting solvent was THF (Sps, H718 L, Thommen Furler). Sample concentration was ∼5 mg/L, injection volume was 100 μL, and flow rate was 1 mL/min. Polystyrene standards were used for calibration (Figures S54 and S55).

## Supporting Information Available

Extended description of experiments; details on synthesis and nomenclature; quantification procedure of NMR data; funPEOS specifications; funPEOS characterization (NMR, FTIR, and GPC); postmodification of funPEOS; preparation of silica foam (PDF)
