# Supplementary material for: Hyperbranched, Functional Polyethoxysiloxanes: Tunable Molecular Building Blocks
Source: ACS Appl Polym Mater. 2024 Jun 14;6(12):7088–101. doi: 10.1021/acsapm.4c00758 (PMC11217919; doi:10.1021/acsapm.4c00758)
Supplement: Supplementary file 1 — ap4c00758_si_001.pdf [file ap4c00758_si_001.pdf]

Supporting information

Hyperbranched, Functional Polyethoxysiloxanes: Tunable Molecular Building Blocks

Marek Nemec[1], Stefanie B. Hauser[1], Daniel Rentsch[3], Gabriel M. Pagotti Joao[1,2], Lilli C. Kuerten[1,2], Nour Adilien[1], Lukas Huber[1], Ana Stojanovic[1], Wim J. Malfait[1]*, Matthias M. Koebel[1, 2]*.

[1] Laboratory of Building Energy Materials & Components, Swiss Federal Laboratories for Materials Science and Technology, Empa, Überlandstrasse 129, CH-8600 Dübendorf, Switzerland

[2] Siloxene AG, Zürichstrasse 38, 8306 Brüttisellen, Switzerland

[3] Laboratory for Functional Polymers, Swiss Federal Laboratories for Materials Science and Technology, Empa, Überlandstrasse 129, CH-8600 Dübendorf, Switzerland

* corresponding authors: Matthias Koebel, m.koebel@siloxene.com, +41 44 545 04 20; Wim Malfait, wim.malfait@empa.ch, +41 58 765 4983

Contents

Supporting information 1

Hyperbranched, Functional Polyethoxysiloxanes: Tunable Molecular Building Blocks 1

1 Experimental 4

1.1 Materials 4

1.2 PEOS/funPEOS synthesis protocol 7

1.2.1 Up scaling protocol 7

1.2.2 Protocol used to prepare funPEOS for ^{29}Si-^{29}Si INADEQUATE NMR measurement 8

1.3 Characterization and data processing 8

2 Alkoxysilane species 10

3 PEOS/funPEOS details 11

3.1 Dynasylan 40 as a starting material for PEOS synthesis 11

3.2 PEOS average mass per Si atom 12

3.3 PEOS/funPEOS nomenclature 13

3.4 Reactant and product amounts 13

3.5 T, M-modified PEOS protocol 18

3.6 Small scale and up-scaled 7 L set-up 18

4 Conditions required for quantitative acquisition of ^{29}Si NMR data 22

5 ^{29}Si NMR data processing and resonance integration 23

5.1 Different NMR data sets recorded for the same batch 23

5.2 Different methods for background correction 24

5.3 NMR data sets processed and evaluated by different operators 28

5.4 Reproducibility of the synthesis 32

5.5 Relative standard uncertainties of the compositions of the funPEOS samples obtained through ^{29}Si NMR 33

6 PEOS/funPEOS specifications 35

6.1 PEOS synthesis: Q speciation and evolution of Q species 35

6.2 Effect of functional group size on grafting efficiency 39

6.3 Ethoxy-methoxy exchange of funPEOS 39

6.4 Mass balance 42

7 PEOS/funPEOS characterization 45

7.1 ^{1}H and ^{13}C NMR of synthesized funPEOS 46

7.2 FTIR characterization 55

7.3 Gel permeation chromatography 60

7.3.1 Double detection: refractive index detector vs. UV detector 60

7.3.2 GPC calibration details ... 60

8 Post-modification of funPEOS ... 63

9 Preparation of silica foam ... 66

References ... 67

1 Experimental

1.1 Materials

Tetraethoxysilane (TEOS) (99 % abcr.), Dynasylan® 40 (D40) (Evonik), acetic anhydride (AA) (99.50 %, Lonza), titanium (IV) isopropoxide (TTIP) (97 % Sigma-Aldrich) were used during the synthesis of PEOS/funPEOS. The following functional silanes were used for functionalization of PEOS: methyltriethoxysilane (MTES) (98 % Sigma-Aldrich), n-propyltriethoxysilane (PTES) (97 %, abcr.), n-propyltrimethoxysilane (PTMS) (97 %, Sigma-Aldrich), n-octyltriethoxysilane (OTES) (97 %, abcr.), hexadecyltrimethoxysilane (HdTMS) (85 %, Sigma-Aldrich), (3,3,3-trifluoropropyl)trimethoxysilane, (FpTMS) (97 %, abcr.), (3-glycidoxypropyl)trimethoxysilane (GLYMO) (98 %, Sigma-Aldrich), vinyltriethoxysilane (VTES) (98 %, abcr.), phenyltrimethoxysilane (PhTMS) (97 %, abcr.), (3-methacryloxypropyl)trimethoxysilane (PMaTMS, 98%; abcr.), bis[3-(triethoxysilyl)propyl] tetrasulfide (Deolink TESPT-100) (DOG), diethoxydimethylsilane (DMDES) (97 %, abcr.) and hexamethyldisiloxane (HMDSO) (98 % abcr.). All reactions were carried out under nitrogen inert atmosphere (PanGas). For post-modification of funPEOS hexamethylenediamine (HMDA), (98 %, Sigma-Aldrich) in a denat. ethanol (ethanol/isopropanol mixture 95/5%) (Alcosuisse) was used as the modification solution. Diluted hydrochloric acid (HCl) (36.5-38 % abcr.), ammonium hydroxide solution (28-30 %, Sigma-Aldrich) and isopropanol (IPA) (Alcosuisse) were used to prepare scratch-resistant coatings. All of the above-mentioned chemicals were used without further purification and are presented in Table S1 together with their chemical structures and CAS numbers.

Table S1: List of materials and abbreviations used in this study with purity, CAS numbers, suppliers and chemical structures.

IUPAC name	Alternative name	abbr.	Purity	CAS N	Supplier	Chemical structure
tetraethyl silicate	tetraethox-ysilane	TEOS	99 %	78-10-4	abcr.	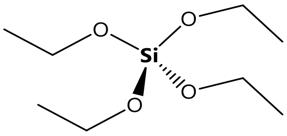
partially polymerized tetraethyl silicate	Dynasylan® 40	D40		684 12-37-3	Evonik	ethyl silicate with a silicon diox-ide content of approximately 40 - 42 % upon complete hydrolysis (the Si content of Dynasylan® 40 is calculated as SiO_2).
acetyl acetate	acetic anhy-dride	AA	99.50 %	108-24-7	Lonza	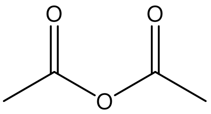
propan-2-olate; tita-nium(4+)	titanium(IV) iso propoxide	TTIP	97%	548-68-9	Sigma-Aldrich	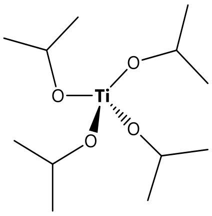
hydroxy(trimethyl)silane;ti-tanium	tetrakis(tri methylsioxy) titanium		95%	15990-66-6	Sigma-Aldrich	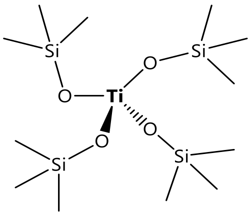
trimethoxy(methyl) silane	methyltri methoxy silane	MTES	98%	2031-67-6	Sigma-Aldrich	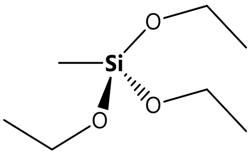
triethoxy(propyl)silane	n-propyltri ethoxysilane	PTES	97%	2550-02-09	abcr	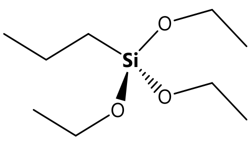
trimethoxy(propyl)silane	n-propyl trimethoxy silane	PTMS	97%	1067-25-0	Sigma-Aldrich	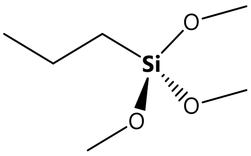

triethoxy(octyl)silane	octyltri ethoxysilane	OTES	97%	2943-75-1	Sigma-Aldrich	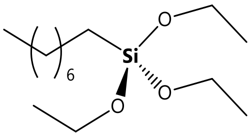
Hexadecyl (trimethoxy)silane	hexadecyltri methoxy silane	HdTMS	85%	16415-12-6	Sigma-Aldrich	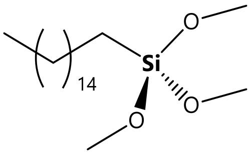
trimethoxy(3,3,3-trifluoro-propyl)silane	(3,3,3-trifluoro propyl)tri methoxy silane	FpTMS	97%	429-60-7	abcr	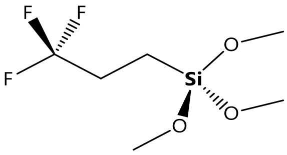
trimethoxy-[3-(oxiran-2-ylmethoxy)propyl]silane	(3-glycidoxy propyl)tri methoxy silane	GLYMO	98%	2530-83-8	Sigma-Aldrich	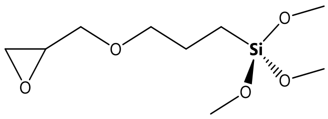
ethenyl(triethoxy)silane	vinyltri ethoxysilane	VTES	98%	78-08-0	abcr	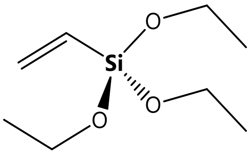
trimethoxy(phenyl)silane	phenyltri methoxy silane	PhTMS	97%	2996-92-1	abcr	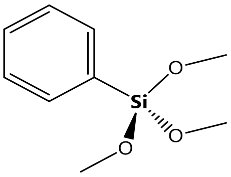
3-trimethoxysilylpropyl 2-methylprop-2-enoate	3-(trimethox-ysilyl)propyl methacrylate	PMaTMS	98%	2530-85-0	abcr	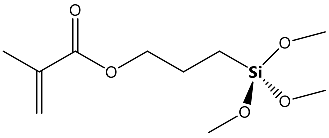
triethoxy-[3-(3-triethoxysi-lylpropyl tetrasulfanyl)propyl] silane	Bis[3-(trieth-oxy silyl)propyl] tetrasulfide	eTESPT		40372-72-3	DOG	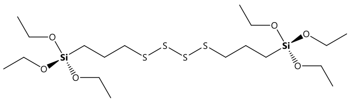
diethoxy(dimethyl)silane	diethoxy dimethyl silane	DMDES	97%	78-62-6	abcr	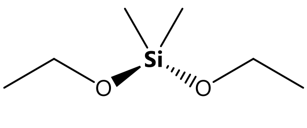

trimethyl(trimethyl silyloxy)silane	hexamethyl disiloxane	HMDSO	98%	107-46-6	abcr	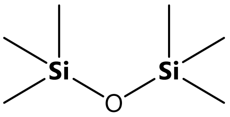
molecular nitrogen	nitrogen gas			7727-37-9	PanGas	N_2
hexane-1,6-diamine	hexameth-ylenediamine	HMDA	98%	124-09-4	Sigma-Al-drich	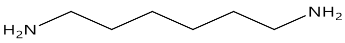
ethanol/ propan-2-ol mix-ture	ethanol/ isopropanol mixture	EtOH/ IPA	95/5%		Alcosuisse	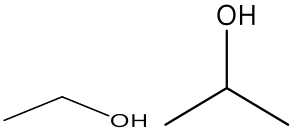
hydrogen chloride	hydrochloric acid		36-38%	7647-01-0	abcr	HCl
azanium;hydroxide	ammonium hydroxide so-lution		28-30%	1336-21-6	Sigma-Aldrich	NH_4OH
propan-2-ol	isopropanol	IPA		67-63-0	Alcosuisse	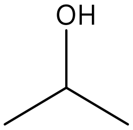

1.2 PEOS/funPEOS synthesis protocol

1.2.1 Up scaling protocol

In order to demonstrate the scalability of the technique, a standard funPEOS preparation was successfully scaled-up inside a 7 liter reactor (Figure S5 and Figure S6). 4000 g of D40 were inserted into the 7 liter reaction vessel heated up to 140 °C under N_2 and mixed with 9.5 ml of TTIP. 448.8 g of acetic anhydride were introduced into the system by peristaltic pump with the speed of addition 20 ml/min. The reaction was observed by monitoring the condensate flow. 85 min after the start of the synthesis, the condensate flow considerably slowed down indicating the end of PEOS carrier growth. 1565.9 g of VTES and 12.6 ml of TTIP were added at once into the reaction vessel. 1262.2 g of acetic anhydride were again introduced into the system by peristaltic pump with a speed of addition 20 ml/min. The purification step consisting of lowering the pressure inside the reaction vessel to 200 mbar for 15 min was carried out in order to remove the residual volatile species inside the funPEOS product. 3 hours after the initial mixing of the reactants, 4050 g of funPEOS product (Figure S8, N50 eV30) and 3150 g of condensate were collected. The resulting oily hydrophobic liquid product had identical chemical Si bonding topology, confirmed by ^{29}Si NMR (Figure S7) measurements, and the same properties (density, viscosity, application properties) as the reference product obtained at laboratory scale using the same synthesis protocol.

The type and amount of reactants used in the individual steps can be freely selected in accordance with the desired composition and molecular weight of the polymeric liquid material to be formed. The silane is typically mixed with acetic anhydride and a catalyst selected from the family of tetraalkoxy titanates $Ti(OR)_4$, e.g. TTIP. Catalyst concentrations are in the range from 0.02 % mol to 1.5 % mol based on the total molar amount of silicon atoms. Additional organofunctional silane/silanes can be added in sequence together with more acetic anhydride to build another

functional layer by repeating the reaction protocol described above. Note that too high acetic anhydride addition leads to gelation / solidification of the system. The acetic anhydride to total silane ratio defines the overall average number of bridging oxygens per Si atom. Similarly, the organofunctional T-type silane to Q-type "tetraalkoxysilane" ratio defines the degree of functionalization. More examples with reactant and product amounts of PEOS are presented in Table S4 and of funPEOS in Table S5.

1.2.2 Protocol used to prepare funPEOS for ^{29}Si-^{29}Si INADEQUATE NMR measurement

Figure 4 of the main text shows ^{29}Si-^{29}Si INADEQUATE NMR spectrum and its projections of a funPEOS with a TEOS based carrier and a MTES based shell. The targeted parameters were selected to be: $DF_{theoretical}$ = 0.60, $BO/Si_{carrier, theoretical}$ = 2.35 and Ac_2O/Si = 1.5. To prepare this product, the following protocol was used:

To prepare a PEOS substrate, 100 g (481 mmol) of TEOS were mixed with 0.33 ml (1.1 mmol) of TTIP catalyst. The reaction mixture was heated to 140 °C in a round-bottom flask in a water free, inert atmosphere (N2) under stirring. Acetic anhydride was gradually added up to a 1.0:0.9 molar ratio (44.2 g, 433 mmol) using peristaltic pump with the speed of addition 0.8 ml/min The reaction side-product i.e. ethyl acetate was distilled off until the flow of the distillate stopped, which indicated the end of the reaction after around 280 min. A pale yellow, oily PEOS liquid and colorless condensate were collected separately. In the second step of this one-pot synthesis 51.4 g (288 mmol) of MTES and 0.33 ml (1.1 mmol) of TTIP were added at once to the round-bottom flask with the obtained PEOS carrier under continuous stirring at 140°C. In addition to this, a second stoichiometric amount of acetic anhydride (AA) in a MTES:AA molar ratio of 1.00:1.50 (44.2 g, 433 mmol) was introduced into the reaction mixture by syringe pump with the speed of addition 0.6 ml/min. While keeping the reaction temperature constant at 140 °C throughout the whole synthesis, further ethyl acetate was formed and distilled off. The reaction was followed by monitoring the formation of the condensate. Once the distillation stopped, the rest of the low-molecular reaction products and residual starting materials in the reaction mixture were removed by vacuum distillation through gradually lowering the pressure inside the reaction vessel and holding the final pressure of 110 mbar at 140° C for 15 minutes. Shell build-up step took 190 min which together with the first step yields 470 min. Colorless condensate and yellow, stable, liquid funPEOS product were obtained. The final funPEOS exhibits the following parameters: DF = 0.38, $BO/Si_{carrier}$ = 2.30 and BO/Si_{shell} = 2.1. Note that the difference between $DF_{theoretical}$ and final DF originates from boiling point of MTES (142 °C) which is similar to the reaction temperature 140 °C, therefore some MTES was distilled off during the synthesis.

1.3 Characterization and data processing

NMR sample preparation and acquisition parameters are presented in the main text (Section 5.2 Characterization).

Processing of NMR data was carried out with the Bruker TopSpin software. Each ^{29}Si NMR spectrum was phase corrected using zero and first order corrections and line broadening was set to 10 Hz. The broad background signal originating from the NMR tube and the probe was corrected by manually setting 20-40 points at a distance of > 2 ppm from signal regions (Figure S12) and, subsequently, cubic spline correction of the baseline was applied. The quantification of ^{29}Si NMR relative peak areas was carried out by integration in the Bruker TopSpin software. For selected examples, data processed in this way were compared with quantitative data obtained for data sets where the background signal of an NMR tube and probe containing a pure ethanol/isopropanol mixture (95/5 %) (Figure S13) was subtracted from the spectra of the analyte (all the other processing and integration steps we kept the same as described above). Consistent results were obtained with both background correction methods and a detailed evaluation of uncertainties can be found in the supporting information Section 5 ^{29}Si NMR data processing and

resonance integration in Figure S10 – Figure S21 and Table S7 – Table S11). In summary, the uncertainties of the molar concentrations of Q and T vary depending on type of spectrum that is being processed and the species that are being evaluated, which is a consequence of widely differing relative signal intensities, peak widths and resolutions, as well as due to the selection of integration and background correction parameters. Because of their typically lower concentration, the uncertainty is generally higher for T species compared to Q species. The reproducibility of the funPEOS ^{29}Si NMR measurements and quantitative evaluation is generally high (estimated error of up to ± 10 % relative) for well-resolved signals with high abundance (i.e. T^1, T^2, Q^1, Q^2, and Q^3) and lower (up to ± 30 % relative) for low intensity or poorly resolved signals (i.e. T^0, T^3, Q^0 and Q^4). For the vinyl- and phenyl-modified funPEOS, the overlap between the T^3 and Q^0 signals introduces additional uncertainty. On the nomenclature of the used symbols: Q^n represents a Si atom coordinated by *n* bridging oxygen atoms and *4-n* non-bridging oxygen atoms; T^n is a Si atom coordinated by *n* bridging oxygen atoms, *3-n* non-bridging oxygen atoms, and 1 carbon atom of its organofunctional group; D^n is a Si atom coordinated by *n* bridging oxygen atoms, *2-n* non-bridging oxygen atoms, and 2 carbon atoms of its organofunctional groups; M^n is a Si atom coordinated by *n* bridging oxygen atoms, *1-n* non-bridging oxygen atoms, and 3 carbon atoms of its organofunctional groups (Scheme S1)[1–4].

Fourier Transform Infrared Spectroscopy (FTIR, Bruker Tensor 27; OPUS Version 8.2 software) was carried out in attenuated total reflectance (ATR) mode. The samples were measured by placing a droplet of pure funPEOS liquid directly onto the diamond analyzer crystal; no specific preparation was required. 32 scans were collected over a spectral range from 350 to 3100 cm^{-1} with a resolution of 4 cm^{-1}.

The molecular weights were analyzed using Gel Permeation Chromatography (GPC) (Agilent Technologies 1260 Infinity) with a refractive index (RI) detector. The data were recorded using Agilent GPC/SEC Software version 1.2.3182.29519 and the evaluation was carried out in Matlab 2019b: the baseline was corrected and molecular weight distribution was analyzed by calculating the mass average molar mass $\bar{M}_w$, number average molar mass $\bar{M}_n$ and the polydispersity index PDI.

The silica foam prepared from three monomers (DMDES, MTES and VTES) and two funPEOS (methyl- and vinyl-functionalized) was analyzed using Brunauer–Emmett–Teller (BET) method and scanning electron microscopy (SEM). The nitrogen adsorption–desorption isotherms were carried out on a Micromeritics 3 Flex (Micromeritics, USA) device and the surface area was calculated using the BET method. Prior to the measurements, the sample was placed in a glass tube and degassed at 110 °C for 10 h under vacuum ($3.5e^{-2}$ mmHg). SEM images were acquired on a FEI Nova NanoSEM 230 instrument (FEI, Hillsboro, Oregon, USA) at an accelerating voltage of 5 kV and a working distance of 5.7 mm. The foam sample had previously been affixed onto a sample holder with carbon tape and coated with 10–15 nm of platinum for imaging.

2 Alkoxysilane species

Scheme S1 presents all the possible silane species containing 0 functional groups (Q species), 1 functional group (T species), 2 functional groups (D species) and 3 functional groups (M species) in different form depending on the amount of Si-O-Si bridges.

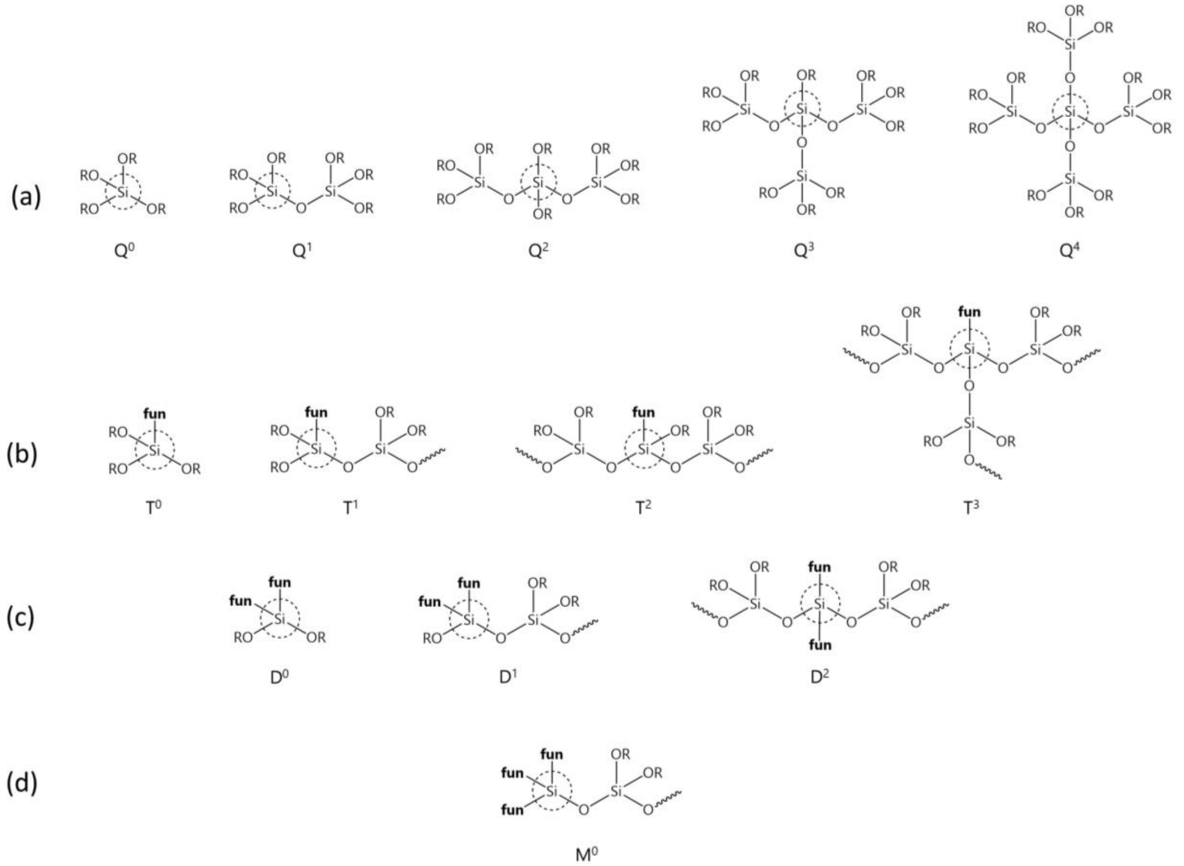

Scheme S1: Basic chemical structures of (a) Q^n species (building blocks of PEOS carrier) and (b) T^m, (c) D^l and (d) M^k species containing organofunctional group (fun)[1–4] presenting building blocks of funPEOS shell.

3 PEOS/funPEOS details

3.1 Dynasylan 40 as a starting material for PEOS synthesis

Commercial product Dynasylan 40 (Evonik) with silica content of around 40 wt% which is significantly higher than 28 wt% in TEOS can be used as an alternative to TEOS as a starting material for PEOS synthesis. In addition to this D40 presents a practical advantage since less Si-O-Si bonds need to be formed to obtain a PEOS substrate structure with a targeted BO/Si. The use of D40 contributes to the lower reaction time, the reduction of acetic anhydride consumption and the ethyl acetate side-product. ^{29}Si NMR of D40 (Figure S1) shows little to no detected silanol species and ^{1}H NMR of D40 (Figure S2) shows a minor signal of ethanol at 3.6 ppm, which overlaps with the satellite of the main CH_2 signal of an ethoxy group and thus D40 is proved to be a valid alternative to TEOS for PEOS synthesis.

Note that silanol species typically appear at higher chemical shifts than their non-silanol analogues, the chemical shifts depend on the number of silanol species (for example Q^0 with two silanol groups occur at higher chemical shift compared to Q^0 with one silanol group). Silanol chemical shifts are at around 73-81 ppm for Q^0, at around 85-87 ppm for Q^1 species, at around 92-94.5 ppm for Q^2 species and at around 99-101 ppm for Q^3 species[5,6].

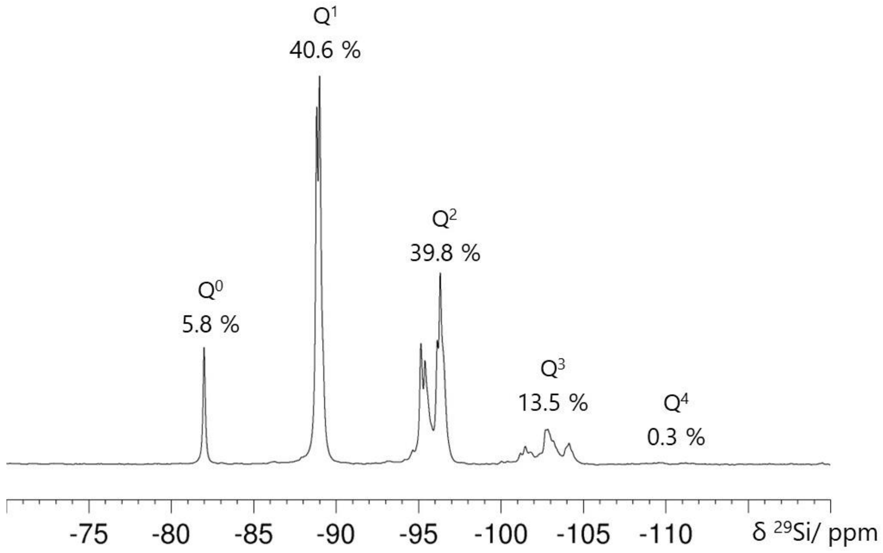

Figure S1 ^{29}Si NMR spectrum of Dynasylan 40.

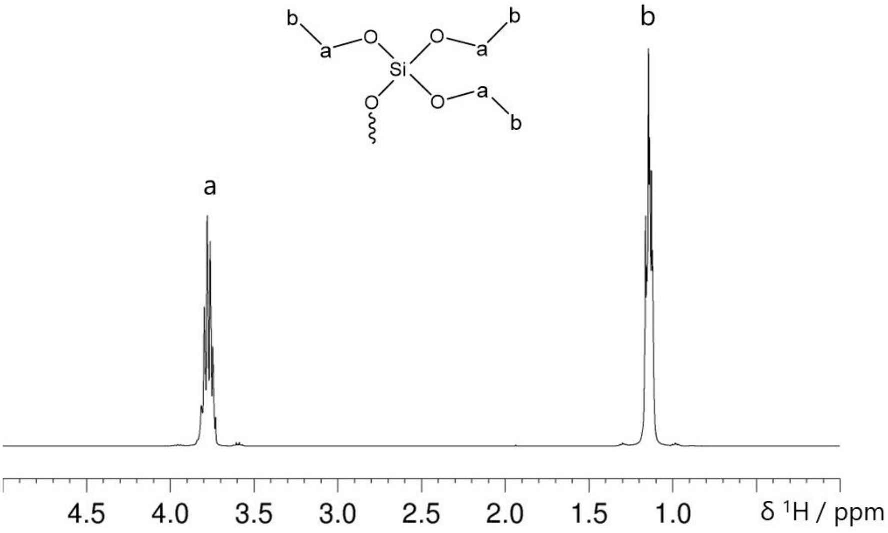

Figure S2: ^{1}H NMR spectrum of Dynasylan 40.

3.2 PEOS average mass per Si atom

Average molar mass per Si atom is calculated using the following formula:

$$M = n(Si) * A(Si) + \ n(BO\ to\ this\ Si) * M(BO\ to\ this\ Si) + n(ethoxy) * M(ethoxy) \qquad \text{(S 1)},$$

where *n* corresponds to the number of either Si atoms, number of bridging oxygens (BO) or number of ethoxy groups. *A(Si)* is mass number of silicon atom and *M* is molar mass of the corresponding group.

For example, D40 exhibits around 40 % of BO, therefore BO/Si value is around 1.6 corresponding to the molecular weight of 148.9 g/mol. PEOS N50 typically exhibits around 50 % of BO, therefore BO/Si value is around 2.0 corresponding to the molecular weight of 134.1 g/mol.

Table S2: number of bridging oxygens and ethoxy groups per one Si atoms and their average molar mass for the various theoretical cases of PEOS molecules.

BO %	average BO per Si / %	SI / n	BO to this Si/ n	Ethoxy/ n	*M*/ g.mol^{-1}	wt% SiO_2
0	0	1	0	4.0	208.1	28.9
5	0.2	1	0.1	3.8	200.7	29.9
10	0.4	1	0.2	3.6	193.3	31.1
15	0.6	1	0.3	3.4	185.9	32.3
20	0.8	1	0.4	3.2	178.5	33.7
25	1.0	1	0.5	3.0	171.1	35.1
30	1.2	1	0.6	2.8	163.7	36.7
35	1.4	1	0.7	2.6	156.3	38.5
40	1.6	1	0.8	2.4	148.9	40.4
45	1.8	1	0.9	2.2	141.5	42.5
50	2.0	1	1.0	2.0	134.1	44.8
55	2.2	1	1.1	1.8	126.7	47.4
60	2.4	1	1.2	1.6	119.3	50.4
65	2.6	1	1.3	1.4	111.9	53.7
70	2.8	1	1.4	1.2	104.5	57.5

75	3.0	1	1.5	1.0	97.1	61.9
80	3.2	1	1.6	0.8	89.7	67.0
85	3.4	1	1.7	0.6	82.3	73.0
90	3.6	1	1.8	0.4	74.9	80.2
95	3.8	1	1.9	0.2	67.5	89.0
100	4.0	1	2.0	0	60.1	100.0

3.3 PEOS/funPEOS nomenclature

In addition to the introductory example of PEOS and funPEOS nomenclature in Section 2 Mechanism, average number of bridging oxygens per Si atom and functionalization of the main text, we present additional examples in Table S3 to further clarify the introduced nomenclature used in this study. Table S3 shows three examples of PEOS substrate material without any shell functionality (i.e. N50, N55 and N60) with their corresponding targeted $BO/Si_{carrier, theoretical}$. Further three examples of funPEOS molecules (i.e. N50 eV15, N55 eM20 and N60 mGly25) are presented with their corresponding targeted $BO/Si_{carrier, theoretical}$, functional alkoxysilane used to introduce the shell functionality, shell functional group (R') with its corresponding abbreviation used in a funPEOS name and the degree of functionalization of funPEOS which is defined as the molar ratio of organofunctional silane (T-type silane) to the Q-type siloxane starting material (e.g. TEOS): $n_{shell}/n_{carrier}$. The last three examples presented in the Table S3 are carrier-double shell funPEOSs (i.e. N50 eV15 eP08, N46 mMa15 eD15 and N50 eV15 TM20) with all the previously mentioned specifics, but this time for both shell functionalities.

Table S3: Examples of PEOS and funPEOS nomenclature.

		1st functionality				2nd functionality			
PEOS/funPEOS name	$BO/Si_{carrier, theoretical}$	fun. group (R')	chemical used for shell	abbr.	$n_{shell}/n_{carrier}$	fun. group (R'')	chemical used for shell	abbr.	$n_{shell}/n_{carrier}$
N50	2.00	-	-	-	-	-	-	-	-
N55	2.20	-	-	-	-	-	-	-	-
N60	2.40	-	-	-	-	-	-	-	-
N50 eV15	2.00	vinyl	VTES	eV	0.15/1.00	-	-	-	-
N55 eM20	2.20	methyl	MTES	eM	0.20/1.00	-	-	-	-
N60 mGly25	2.40	glycidoxy propyl	GLYMO	mGly	0.25/1.00	-	-	-	-
N50 eV15 eP08	2.00	vinyl	VTES	eV	0.15/1.00	propyl	PTES	eP	0.08/1.00
N46 mMa15 eD15	1.84	meth acrylate	PMaTMS	mMa	0.15/1.00	methyl	DMDES	eD	0.15/1.00
N50 eV15 TM20	2.00	vinyl	VTES	eV	0.15/1.00	methyl	HMDSO	TM	0.20/1.00

3.4 Reactant and product amounts

Table S4 shows mass balance with reactant amounts used for selected examples of PEOS carrier syntheses with different $BO/Si_{carrier}$. In all cases D40 was mixed with TTIP catalyst and the reaction mixture was heated up to 140°C

under N_2. Acetic anhydride was added by peristaltic pump during time range of 25 – 45 min. Side product ethyl acetate was formed and distilled off until the flow of the distillate stopped, which indicated the end of the reaction (time of the reaction depended on the amount of acetic anhydride used, i.e. Ac_2O/Si ratio). Volatile reaction products and residual starting materials in the reaction mixture were removed by vacuum distillation through gradually lowering the pressure inside the reaction vessel and holding a final pressure of 110 mbar at 140 °C for 15 minutes.

Selected funPEOS syntheses examples with reactant amounts for PEOS substrate step as well as reactant amounts for organofunctional grafting together with amounts of isolated products are presented in Table S5. The reaction protocol used for the first step of the funPEOS syntheses (PEOS substrate step) followed the reaction steps described in the first paragraph of this section. For the shell grafting step, functional alkoxysilane together with additional TTIP was added at once to an obtained PEOS substrate in the same reaction vessel that was used for the substrate step using the same temperature (140 °C) while maintaining the N_2 atmosphere. Note that in case of GLYMO grafting, the reaction temperature needs to be around 110°C to avoid the reaction between epoxy group and anhydride present in the mixture, therefore also the time for grafting step was increased. In case of functional silanes with lower boiling points, e.g. MTES, the temperature needs to be selected wisely in order not to distil over the monomeric T^0 and therefore lose the functionality in the final funPEOS. Following with the reaction protocol, acetic anhydride was again added by peristaltic pump during time range of 25 – 45 min. Volatile side products were distilled off until the flow of the distillate stopped, which indicated the end of the reaction (time of the reaction depended on the amount of acetic anhydride used, i.e. Ac_2O/Si ratio). Volatile products and residual starting materials in the reaction mixture were removed by vacuum distillation through gradually lowering the pressure inside the reaction vessel and holding a final pressure of 110 mbar at 140 °C for 15 minutes.

Since the poly-condensation reaction of alkoxysilanes via acetic anhydride route (Scheme S2 for PEOS and Scheme 1 of the main text for funPEOS synthesis) is quantitative in terms of its reagents, it is possible to directly calculate the expected amount of by-product ethyl acetate (EtOAc) (or in case of methoxy-based silane it is methyl acetate) using the following formula:

$$m(EtOAc)_{theory} = 2 \cdot (n(\text{AA in carrier step}) + n(\text{AA in shell step}) \ \cdot M(\text{EtOAc}) \qquad \text{(S 2)},$$

where $m(EtOAc)_{theory}$ is theoretical weight of ethyl acetate, $n(\text{AA in carrier step})$ and $n(\text{AA in shell step})$ are molar amounts of acetic anhydride used in quantitative carrier step and shell step respectively and$\cdot$ $M(\text{EtOAc})$ is molar weight of ethyl acetate.

Note that in case of higher acquired mass of funPEOS product compared to the theoretical mass, the funPEOS product still contains by-product, which was not distilled off completely. This can be easily resolved by either more extreme conditions during purification step for longer time (i.e. rotary evaporator at low pressure), bubbling nitrogen through the liquid product at elevated temperature to get rid of the trapped low molecular compounds or by using molecular sieves. The opposite case of collecting less funPEOS than theoretical amount is caused by the evaporation of a functional silane monomer. This topic is discussed in more detail in Section 3.2.4 Scale-up and mass balance of the main text and Section 3.6 Small scale and up-scaled 7 L set-up of this supporting information. Mass losses in final products compared to the initial mass of reactants might be caused by leakage in a system causing volatile compounds to escape from the set-up, problems with collection of final funPEOS products from a reaction vessel (in case of viscous liquid products) or problems with applying lower pressure inside the reaction vessel and holding a final pressure for several minutes where the distillation might not be efficient enough and some of the volatile compounds continue in a gas phase further through the vacuum pump without being collected.

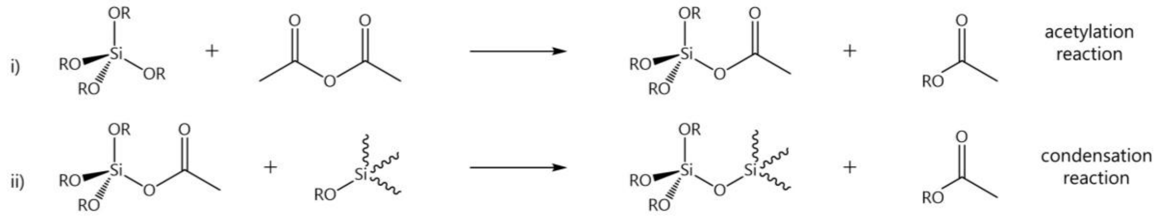

Scheme S2. Hyperbranched PEOS growth via anhydride route consisting of acetylation and condensation step.

Table S4: Reactant and product amounts for exemplary PEOS carrier syntheses.

		reactants	products											
PEOS	BO/$Si_{carrier, theoretical}$	*n* (D40) (mol)	*n* (TEOS) (mol)	*m* (D40) *(g)*	*m* (TEOS) (g)	Ac_2O/Si-ratio	*n* (AA) (mol)	*m* (AA) (g)	*V* (TTIP) ml	*m* (PEOS) theory (g)	*m* (PEOS) collected (g)	*m* (EtOAc.) theory (g)	*m* (cond.) collected (g)	Appearance
N60	2.4	-	1.1	-	232.0	1.2	1.3	136.7	0.64	132.8	142.0	235.9	222.1 g	yellow liquid
N55	2.2	35.0	-	5100.0	-	1.1	9.1	929.8	12.00	4425.0	4248.0	1604.9	1590.0	yellow liquid
N50	2.0	37.8	-	5500.0	-	1.0	6.0	617.1	13.00	5052.0	5125.0	1065.1	1001.0	yellow liquid, see Figure S8

Table S5: Reactant and product amounts for exemplary funPEOS carrier-shell syntheses.

carrier step							shell step								products				
funPEOS	*n*(D40) (mol)	*m*(D40) (g)	Ac_2O/Si_{ratio}	*n*(AA) (mol)	*m*(AA) (g)	*V*(TTIP) (ml)	fun silane	$n_{shell}/n_{carrier}$	*n*(fun) (mol)	*m*(fun) (g)	Ac_2O/Si_{ratio}	*n*(AA) (mol)	*m*(AA) (g)	*V*(TTIP) (ml)	*m*(fun-PEOS) theory (g)	*m*(fun-PEOS) collected (g)	*m*(EtOAc.) theory (g)	*m*(cond.) collected (g)	Appearance
N50 eM30	27.5	4000.0	1.0	4.4	448.8	9.5	MTES	0.3	8.2	1469.5	1.75	14.4	1472.6	12.6	4074.7	3711.0	3316.2	3700.0	Yellow oily liquid
N50 eO15	30.2	4400.0	1.0	4.8	493.7	10.4	OTES	0.15	4.5	1253.3	1.75	7.9	809.9	20.8	4707.0	4839.0	2250.0	2044.0	Yellow oily liquid, see Figure S8
N50 eV30	27.5	4000.0	1.0	4.4	448.8	9.5	VTES	0.3	8.2	1565.9	1.50	12.4	1262.2	12.6	4323.8	4050.0	2953.1	3150.0	Yellow oily liquid
N50 mGly15	0.69	100.0	1.0	0.11	11.2	0.39	GLYMO	0.15	0.10	24.3	1.25	0.13	13.1	0.24	106.7	110.2	42.1	34.6	Yellow oily liquid, see Figure S8

*Note that in case of theoretical TEOS example $BO/Si_{TEOS} = 0$, however D40 exhibits $BO/Si_{D40} = 0.80$ (data from Table S2), therefore as described in the main text, fewer Si-O-Si linkages need to be formed to obtain a final PEOS substrate. When using D40 as a starting material, Ac_2O/Si ratios always take into account the fact that BO/Si_{D40} is 0.80 and not 0 (case for TEOS).

3.5 T, M-modified PEOS protocol

T, M-modified PEOS was prepared by mixing 33.3 g vinyl modified PEOS carrier with BO/$Si_{carrier}$ = 2 (N50 eV15) which was synthesized using standard procedure described in Section 1.2 PEOS/funPEOS synthesis protocol of this supporting information, in a boiling flask with 5 g hexamethyldisiloxane (HMDSO, i.e. source of TMS groups), 16.6 g of ethanol and 0.5 g of 4 M HCl for 40 min at 120°C under reflux condenser. The residual ethanol and HMDSO were distilled off using rotary evaporator (45°C, 110 mbar, 30 min). This way N50 eV15 TM20 was prepared, ^{29}Si NMR recorded from this sample is presented in Figure 7 (d) in the main text.

3.6 Small scale and up-scaled 7 L set-up

The funPEOS synthesis was carried out according to the protocol described in this supporting information Section 1.2 PEOS/funPEOS synthesis protocol, either at laboratory small-scale set-up as shown in Figure S3 and Figure S4, or in larger scale as shown in Figure S5 and Figure S6. Figure S8 shows small-scale synthesized funPEOS examples.

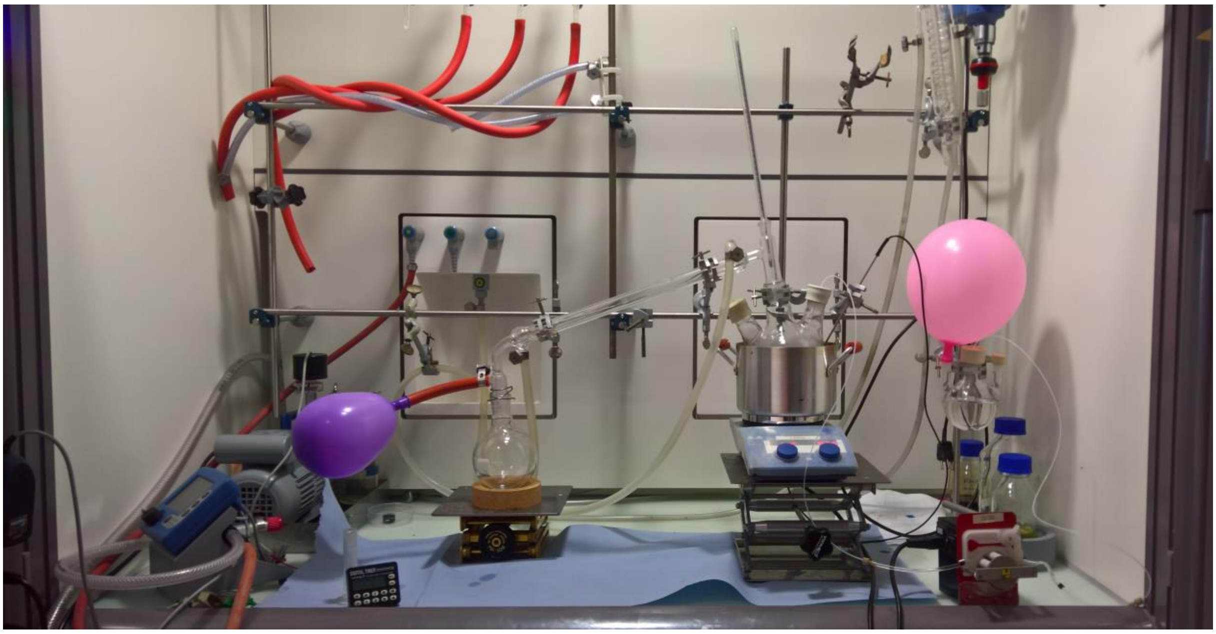

Figure S3: Laboratory small-scale reaction set-up for funPEOS synthesis.

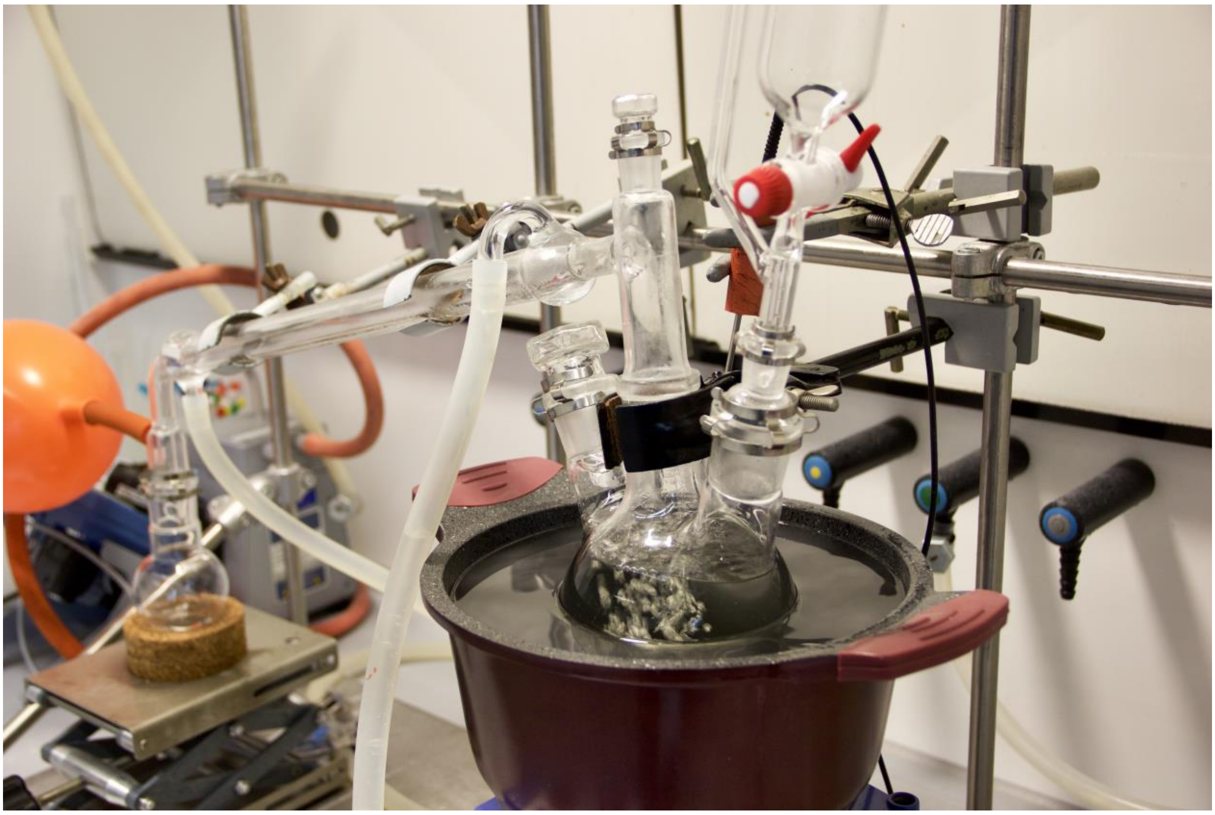

Figure S4: Set-up from Figure S3 from a different perspective.

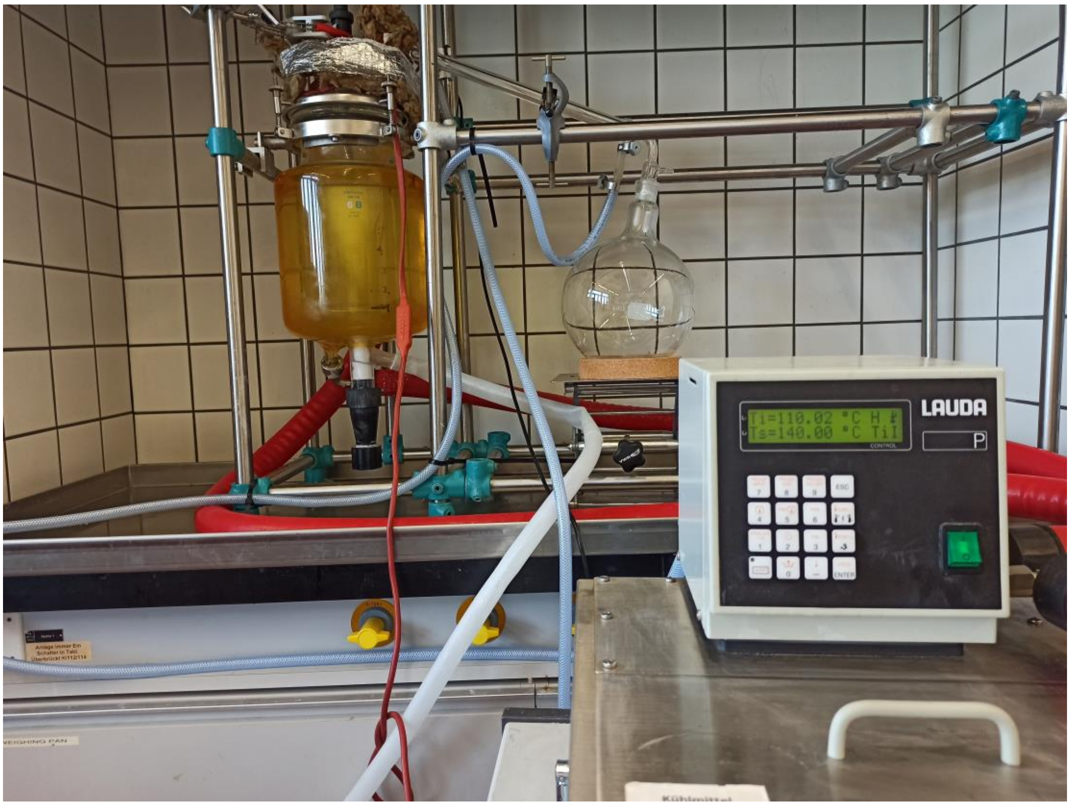

Figure S5: Reaction setup of funPEOS on a larger scale in a reactor with Lauda heating system.

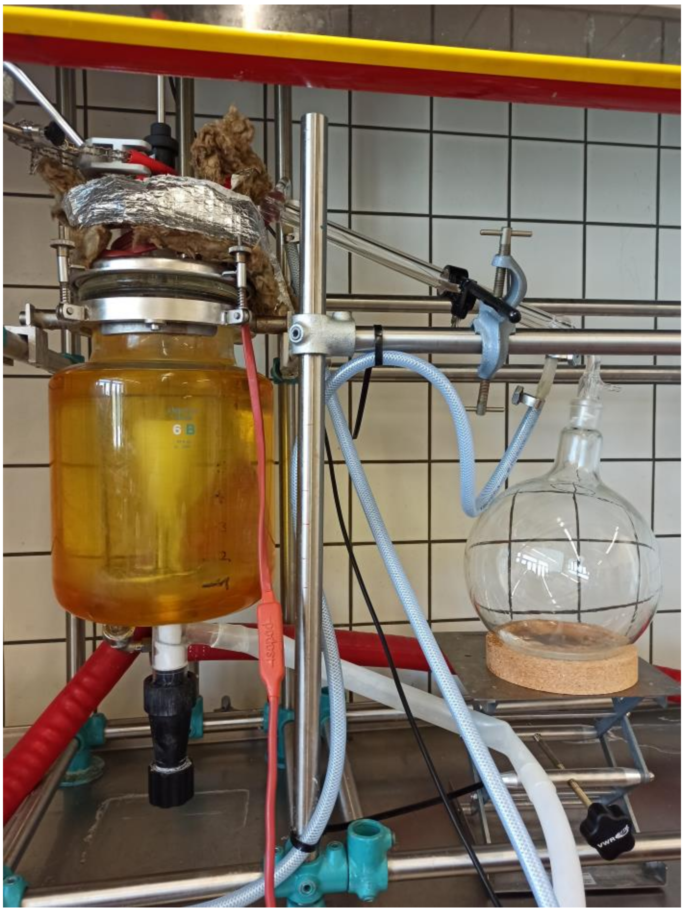

Figure S6: Setup from Figure S5 from a different perspective.

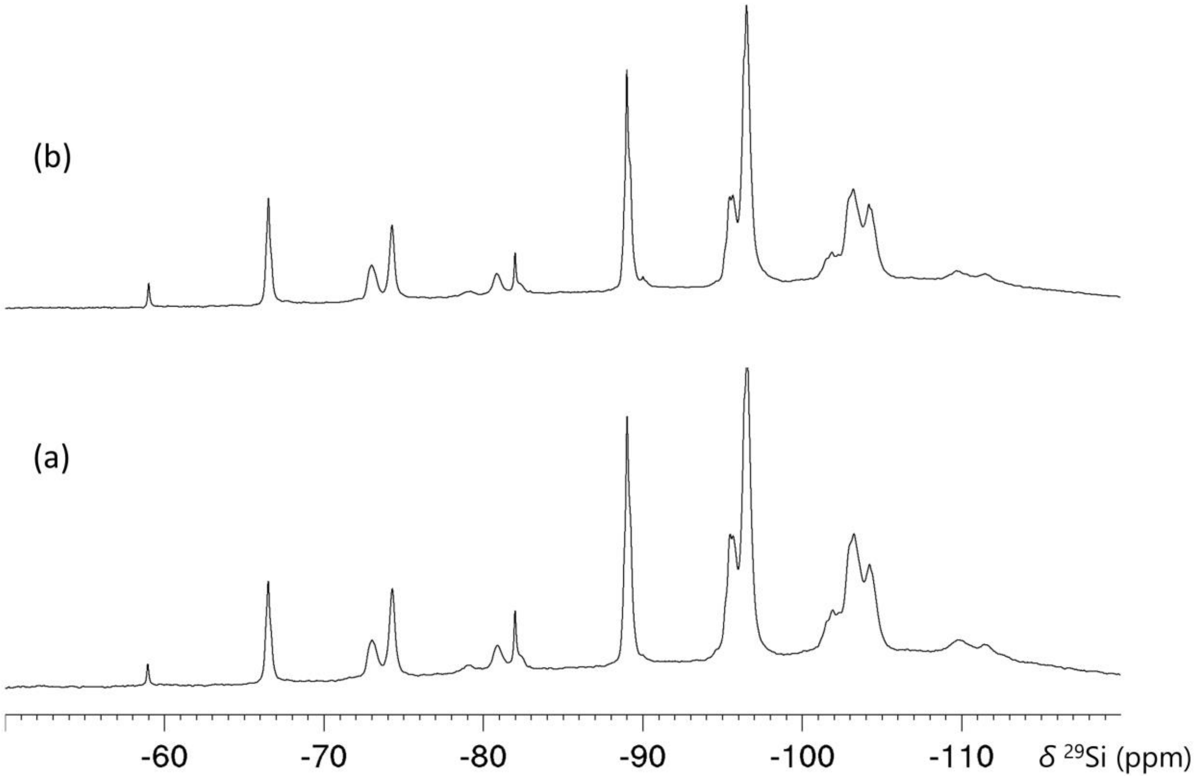

Figure S7: ^{29}Si NMR spectra of (a) N50 eV30 synthesized in the 7 L reactor (4000 g of initial D40) and (b) N50 eV30 synthesized in a small-scale batch (40 g of initial D40).

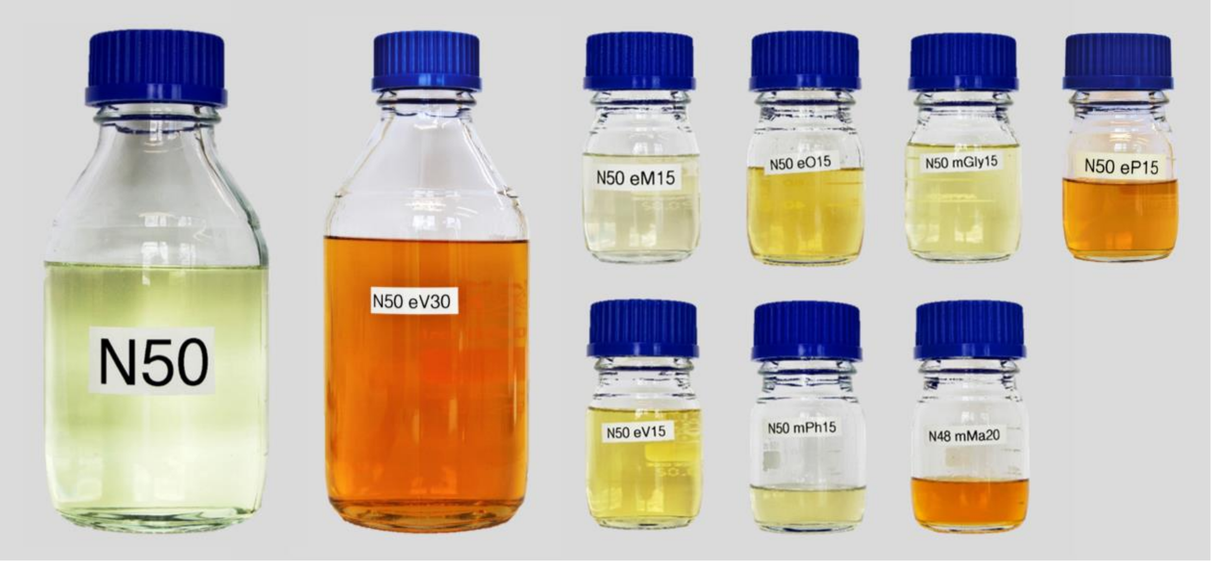

Figure S8: Photographs of synthesized funPEOS material.

Note that color of the final product is highly dependent on the amount of TTIP used during a funPEOS synthesis and the total time of the funPEOS synthesis.

4 Conditions required for quantitative acquisition of ^{29}Si NMR data

^{29}Si NMR sample consisting of N55 eM15 and 15 mM Cr^{3+} was prepared following the sample preparation protocol described in Section 5.2 Characterization of the main text and Section 1.3 Characterization and data processing of this supporting information (^{29}Si NMR sample was prepared by mixing a 0.1 molar stock solution of chromium(III) acetylacetonate as a relaxation agent in chloroform-d ($CDCl_3$, 99.8 % + Ag, Deutero) and given funPEOS sample in a 15:85 volume ratio resulting in a 15 mM Cr^{3+} concentration). Three different ^{29}Si NMR spectra of the same N55 eM15 sample (Figure S9) were recorded using the same protocol as described in the Section 5.2 Characterization of the main text and Section 1.3 Characterization and data processing of this supporting information, 1024 scans with a pulse angle of (π/6), the total recycle delay was changed. Three different total recycle delays: 3.8 s, 6.3 s and 10.3 s were used for the same sample to validate the reproducibility of the measurement and exclude any influence of the recycling times on the relative NMR signal intensities. The obtained spectra were processed using manual setting of 26 points at a distance of > 2 ppm from signal regions (Figure S12) and, subsequently, cubic spline correction of the baseline was applied to get rid of the broad background signal originating from the NMR tube and probe. As shown in Figure S9 and Table S6, no change in relative peak intensities was observed for longer recycle delays. Differences in relative amounts of individual Q and T species as well as subsequent calculated values of $BO/Si_{carrier,}$ and BO/Si_{shell} are caused by the background correction and integration uncertainty further explained in Section 5 ^{29}Si NMR data processing and resonance integration.

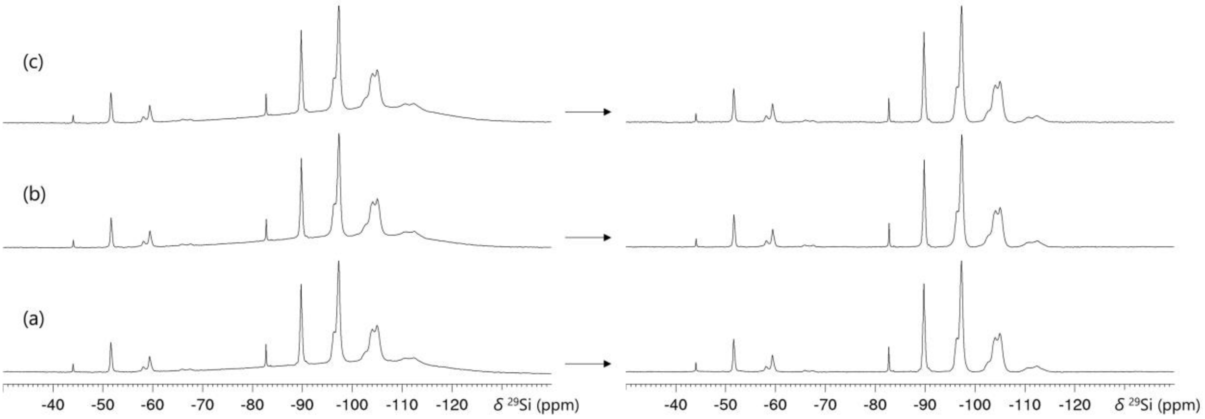

Figure S9: ^{29}Si NMR spectra recorded from the same N55 eM15 sample with different total recycle delay: (a) 3.8 s, (b) 6.3 s, (c) 10.3 s before (left) and after background removal (right).

Table S6: Relative peak intensities for different recycle delays during ^{29}Si NMR measurement.

Recycle delay (s)	T^0 (%)	T^1 (%)	T^2 (%)	T^3 (%)	Q^0 (%)	Q^1 (%)	Q^2 (%)	Q^3 (%)	Q^4 (%)	$BO/Si_{carrier}$	BO/Si_{shell}
3.8	0.9	5.3	5.0	1.2	1.9	14.4	35.6	29.9	5.8	2.2	1.5
6.3	0.8	5.0	5.4	1.2	1.8	14.1	35.5	30.0	6.2	2.3	1.6
10.3	0.7	5.3	5.4	1.1	1.7	14.2	35.4	29.9	6.3	2.3	1.5

5 ^{29}Si NMR data processing and resonance integration

After chemical shift assignments, the relative molar amounts of individual Q and T species can be determined by integration of the ^{29}Si NMR spectra and subsequently, values for BO/Si$_{carrier}$ and BO/Si$_{shell}$ can be obtained. To evaluate the standard uncertainty of the quantitative ^{29}Si NMR data measurement and spectra processing of a synthetic funPEOS product, we identified four main contributing factors:

- Errors originating from the NMR measurement
- Errors due to individual processing and quantification of NMR spectra by different users
- Errors from the use of different background correction methods
- Errors related to the reproducibility of the synthesis (degree of agreement between the synthesis products obtained by different people, in different places and with a new set-up)

5.1 Different NMR data sets recorded for the same batch

Three ^{29}Si NMR spectra were recorded for the same batch of the N50 mPr10 product (Figure S10). All spectra were processed in the same way using manual setting of 26 points at a distance of > 2 ppm from signal regions (Figure S12) and, subsequently, cubic spline correction of the baseline was applied to get rid of the broad background signal originating from an NMR tube and probe. When superimposed, almost identical ^{29}Si NMR spectra were obtained and subsequently, signals of individual species were integrated applying the same integral regions for all three spectra. Sample composition (relative amounts of T and Q species) as well as calculated BO/Si values with corresponding standard deviations (Stdev, σ) (Equation S3) are presented in Figure S11. In general, the relative abundances of Q species and the BO/Si$_{carrier}$ values show smaller standard deviations, than the data obtained for T species and the directly dependent BO/Si$_{shell}$ values.

$$\sigma = \sqrt{\frac{\Sigma(x_i - \mu)^2}{N}} \qquad \text{(S 3)}$$

$$\sigma_{rel} = \frac{\sigma}{\mu} * 100\,, \qquad \text{(S 4)}$$

where σ is population standard deviation, N is size of the population, x_i is each value from the population, μ is the population mean and σ_{rel} is relative population standard deviation in %.

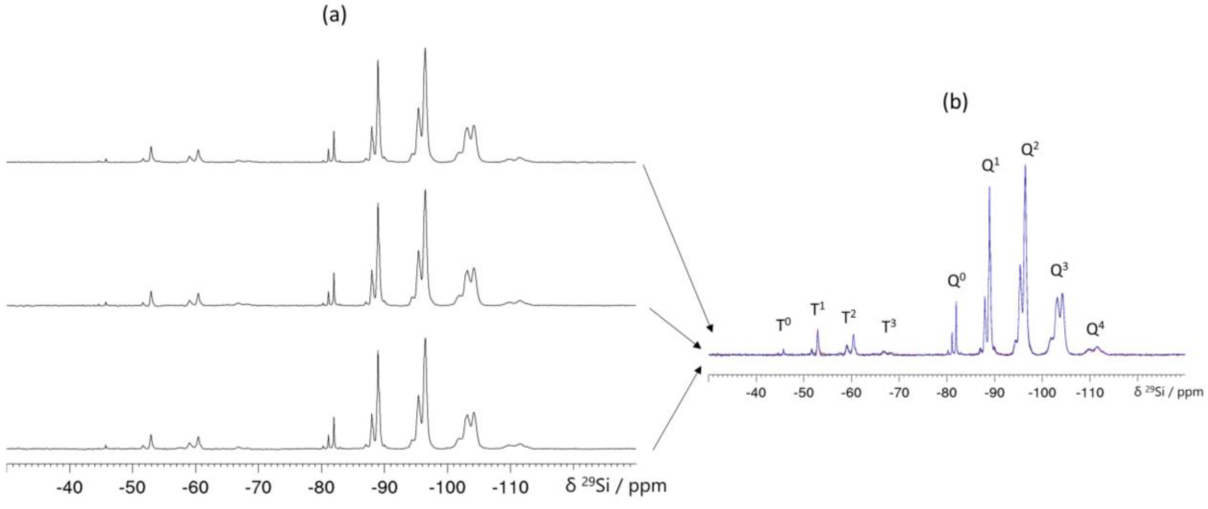

Figure S10: (a) ^{29}Si NMR spectra obtained from three NMR tubes made with the same batch of N50 mPr10 and processed by cubic spline correction of the baseline and (b) superimposed data sets of the spectra shown in (a).

Table S7: Population mean with standard deviation (Equation S3) relative population standard deviation (Equation S4) of the individual T and Q species and corresponding BO/Si obtained from repeated N50 mPr10 ^{29}Si NMR data evaluation (graph shown in Figure S11).

Species	Mean (%)	Stdev ± (%)	Relative Stdev ± (%)	BO/Sitype	Mean	Stdev ±	Relative Stdev ± (%)
T^0	0.4	0.03	8.3	$BO/Si_{carrier}$	2.07	0.011	0.54
T^1	2.9	0.26	9.2	BO/Si_{shell}	1.66	0.048	2.87
T^2	4.1	0.30	7.3				
T^3	0.9	0.28	32.1				
Q^0	3.3	0.16	4.8				
Q^1	20.6	0.40	1.9				
Q^2	38.0	0.18	0.5				
Q^3	25.7	0.28.	1.1				
Q^4	4.1	0.42	10.1				

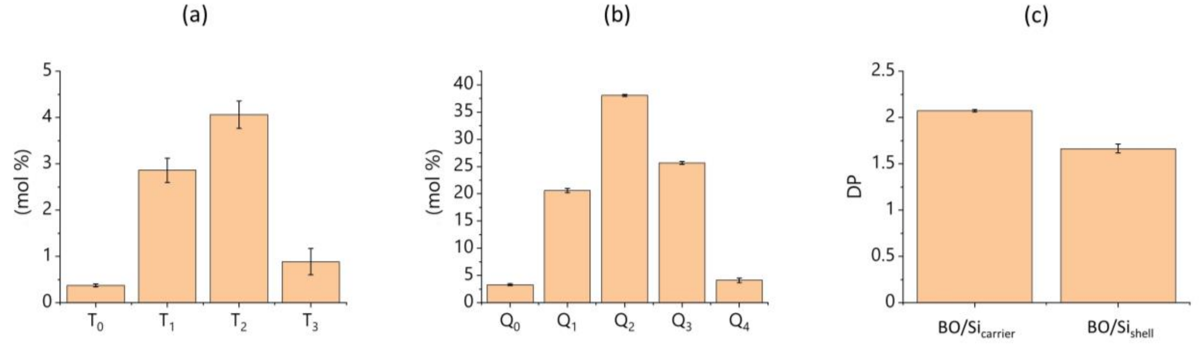

Figure S11: Relative amounts with standard deviations of (a) T, (b) Q species, (c) $BO/Si_{carrier}$ and BO/Si_{shell} obtained from ^{29}Si NMR spectra of three independently prepared samples of one batch of N50 mPr10.

5.2 Different methods for background correction

Two different background correction methods carried out under the TopSpin 3.2 software from Bruker have been used to perform baseline correction: i) manual setting of 20-40 points at distances > 2 ppm from signal regions (Figure S12) followed by cubic spline correction of the baseline and ii) subtraction of a ^{29}Si NMR spectrum recorded under identical NMR experimental conditions of an NMR tube filled with ethanol/isopropanol mixture (95/5 %) (Figure S13) from the data set recorded for funPEOS samples. Here we compare these two background correction techniques and their impact on standard deviation during funPEOS NMR spectra evaluation. The following differently substituted funPEOS molecules were selected to give three fundamentally different types of ^{29}Si NMR spectra, so that each spectrum represents a group of similar products:

1. N50 eM15: example for alkyl chains (Figure S14 a)
2. N50 mGly15: additional ^{29}Si NMR resonances due to methoxy-ethoxy exchange (Figure S14 b)
3. N50 eV15: partial overlap of signals (Figure S14 c)

Each of the recorded ^{29}Si NMR spectra was processed twelve times applying i) cubic spline correction and ii) subtraction of the background signal of the NMR tube / probe. From the data we then calculated sample compositions with standard deviations for each of the funPEOS materials (Figure S15 and Figure S16).

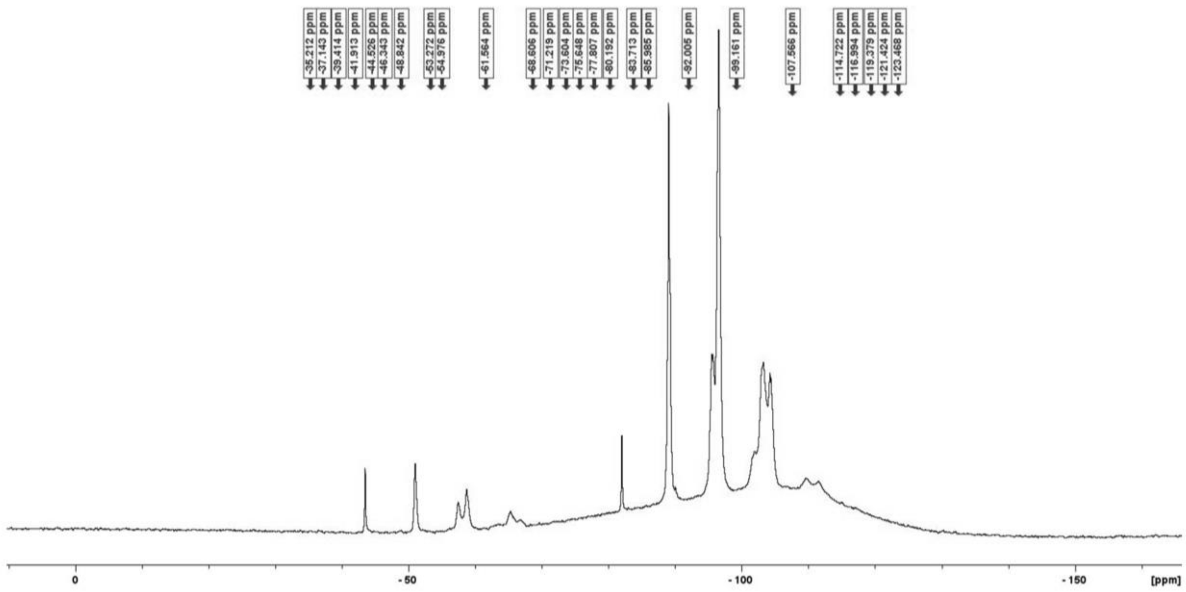

Figure S12: ^{29}Si NMR spectrum of N50 eM15 showing an example of manual setting of 26 baseline points at distances > 2 ppm from signal areas.

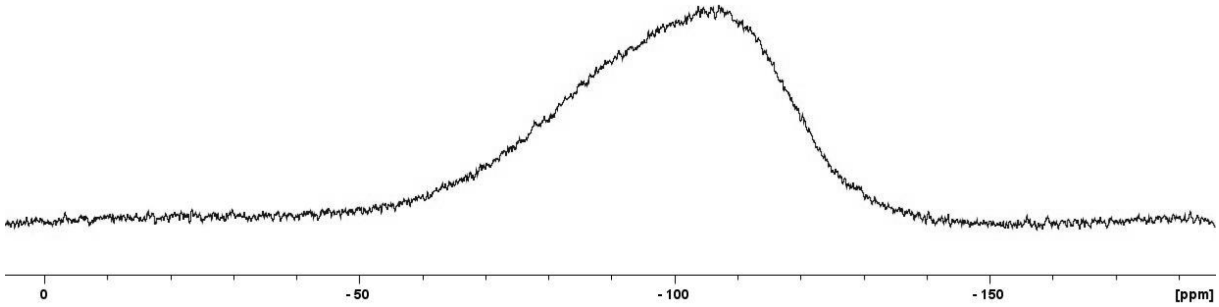

Figure S13: ^{29}Si NMR spectrum of an ethanol/isopropanol mixture (95/5 %) recorded with identical NMR parameters as applied for the data of "real" funPEOS samples used for the subtraction of the background signal of the NMR tube / probe from the funPEOS ^{29}Si NMR spectra.

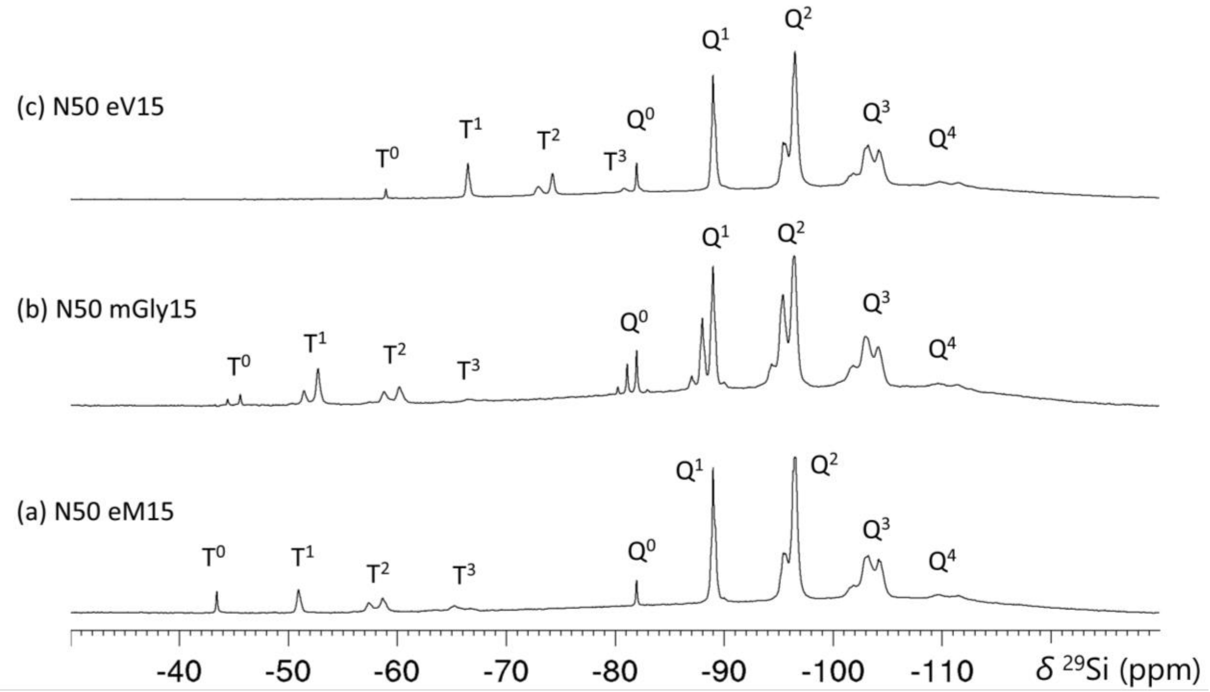

Figure S14: ^{29}Si NMR spectra (prior to baseline correction) with resonance assignments of three fundamentally different funPEOS types, each representing groups of similar products.

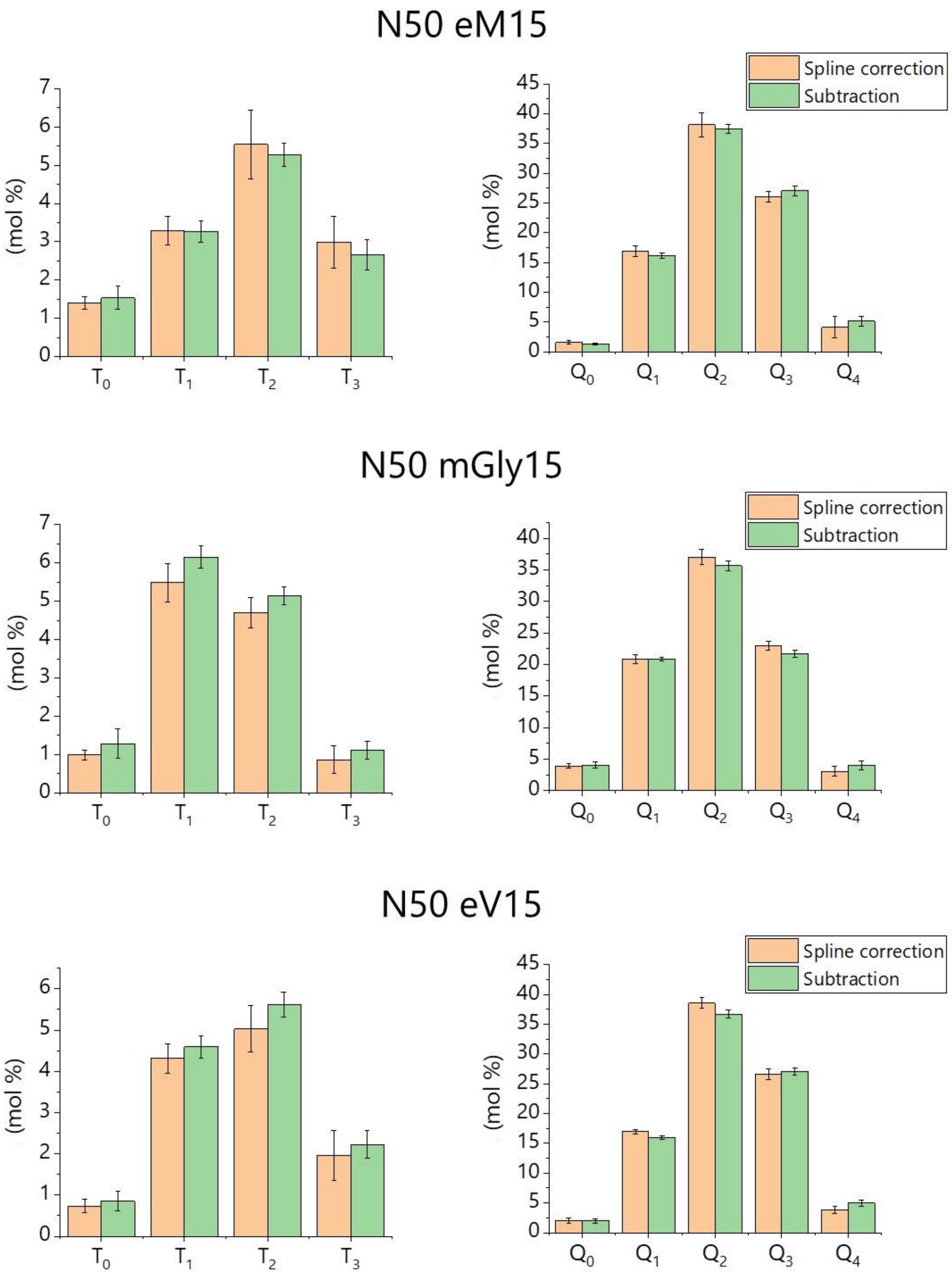

Figure S15: Average relative amounts with standards deviations of T and Q chemical species of three funPEOS samples evaluated after applying spline background correction (orange bars) or processed with subtraction of the background signal of the NMR tube / probe (green bars).

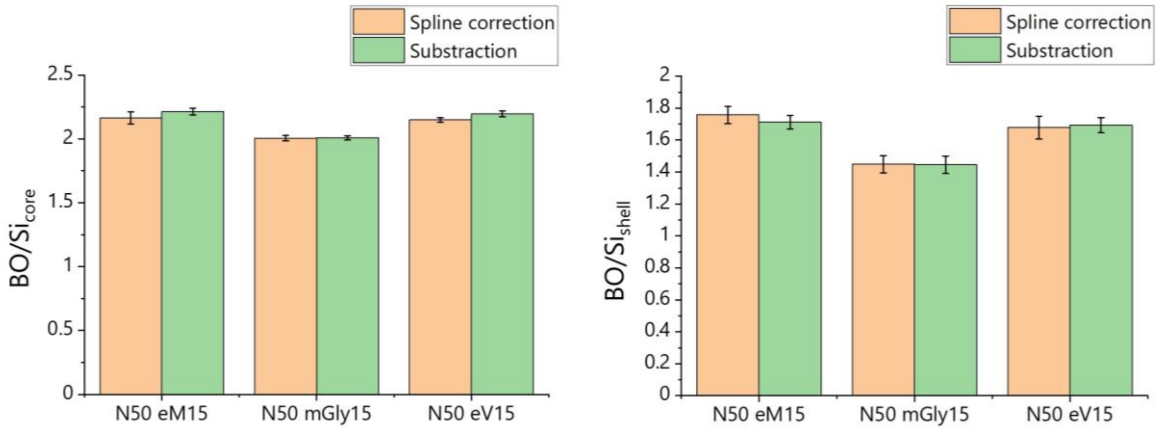

Figure S16: Average BO/Si$_{carrier}$, BO/Si$_{shell}$ values and standards deviations of three funPEOS samples evaluated after applying spline background correction (orange bars) or processed with subtraction of the background signal of the NMR tube / probe (green bars).

5.3 NMR data sets processed and evaluated by different operators

Due to partially subjective nature of the quantification procedure, three different operators independently evaluated unprocessed NMR spectra in order to determine the uncertainty of NMR data evaluations. Three different funPEOS products were synthesized and from each product ^{29}Si NMR data were acquired. The following differently substituted funPEOS molecules were selected to give three fundamentally different types of spectra, so that each spectrum represents a group of similar products: N50 eM15, N50 mGly15 and N50 eV15 (Figure S14).

Each operator evaluated each spectrum four times (with an interval of one week between each evaluation), using both described methods for background correction each time; i.e. i) manual setting of ca. 30 baseline points at a distance of > 2 ppm from signal regions (Figure S12) with cubic spline correction and ii) background correction by subtracting the background signal of the NMR tube / probe (Figure S13). For each case the individual operators acquired the relative molar % of individual Q and T species and calculated the BO/Si$_{carrier}$ and BO/Si$_{shell}$ values. Finally, population means of individual Q and T molar % as well as BO/Si$_{carrier}$ and BO/Si$_{shell}$ values were obtained with standard deviations. These data are presented in the form of tables (Table S8, Table S9 and Table S10) and diagrams in the following figures (Figure S17, Figure S18 and Figure S19).

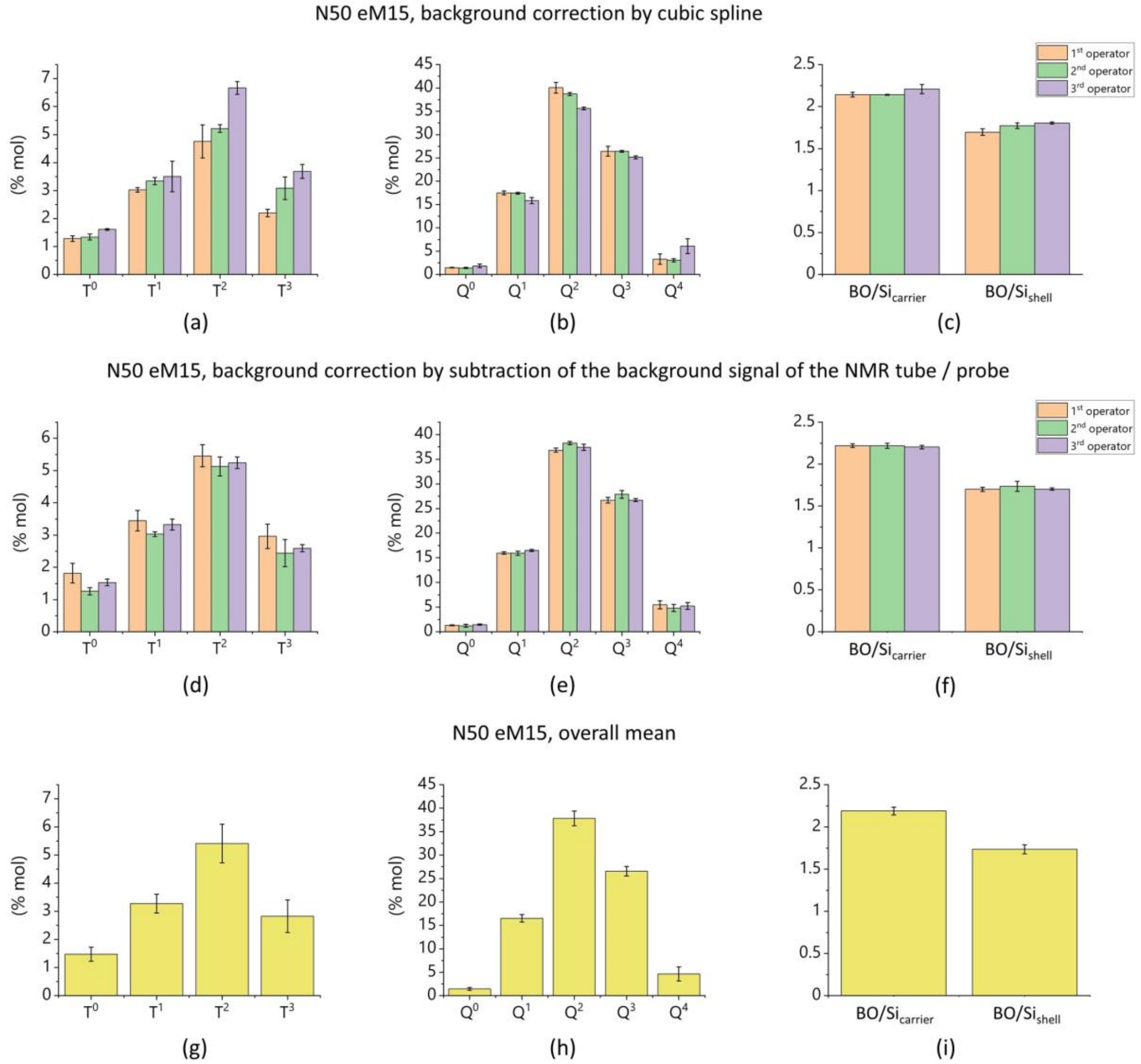

Figure S17: Average relative amounts with standard deviations of (a, d) T, (b, e) Q species, and (c, f) average number of bridging oxygens per Si atom of carrier and shell in sample N50 eM15. The data processing was performed independently by three different operators, each performing four trials of spline background correction (a-c) or subtraction of the background signal of the NMR tube / probe (d-f). Overall average relative amounts with standard deviations of data are shown in (g) T and (h) Q chemical species, and in (i) $BO/Si_{carrier}$ and BO/Si_{shell} values.

Table S8: Overall mean with standard deviation of the individual chemical species and BO/Si values obtained from the repeated data processing and quantitative analysis of the ^{29}Si NMR data of sample N50 eM15 (data used for Figure S17 g-i).

Species	Mean (%)	Stdev ± (%)	Relative Stdev ± (%)	BO/Si type	Mean	Stdev ±	Relative Stdev ± (%)
T^0	1.5	0.25	17.0	$BO/Si_{carrier}$	2.19	0.046	2.09
T^1	3.3	0.33	10.2	BO/Si_{shell}	1.73	0.053	3.08
T^2	5.4	0.68	12.6				
T^3	2.8	0.58	20.4				
Q^0	1.5	0.29	19.8				
Q^1	16.5	0.79	4.8				
Q^2	37.8	1.54	4.1				
Q^3	26.5	1.01	3.8				

Q^4	4.7	1.48	31.7

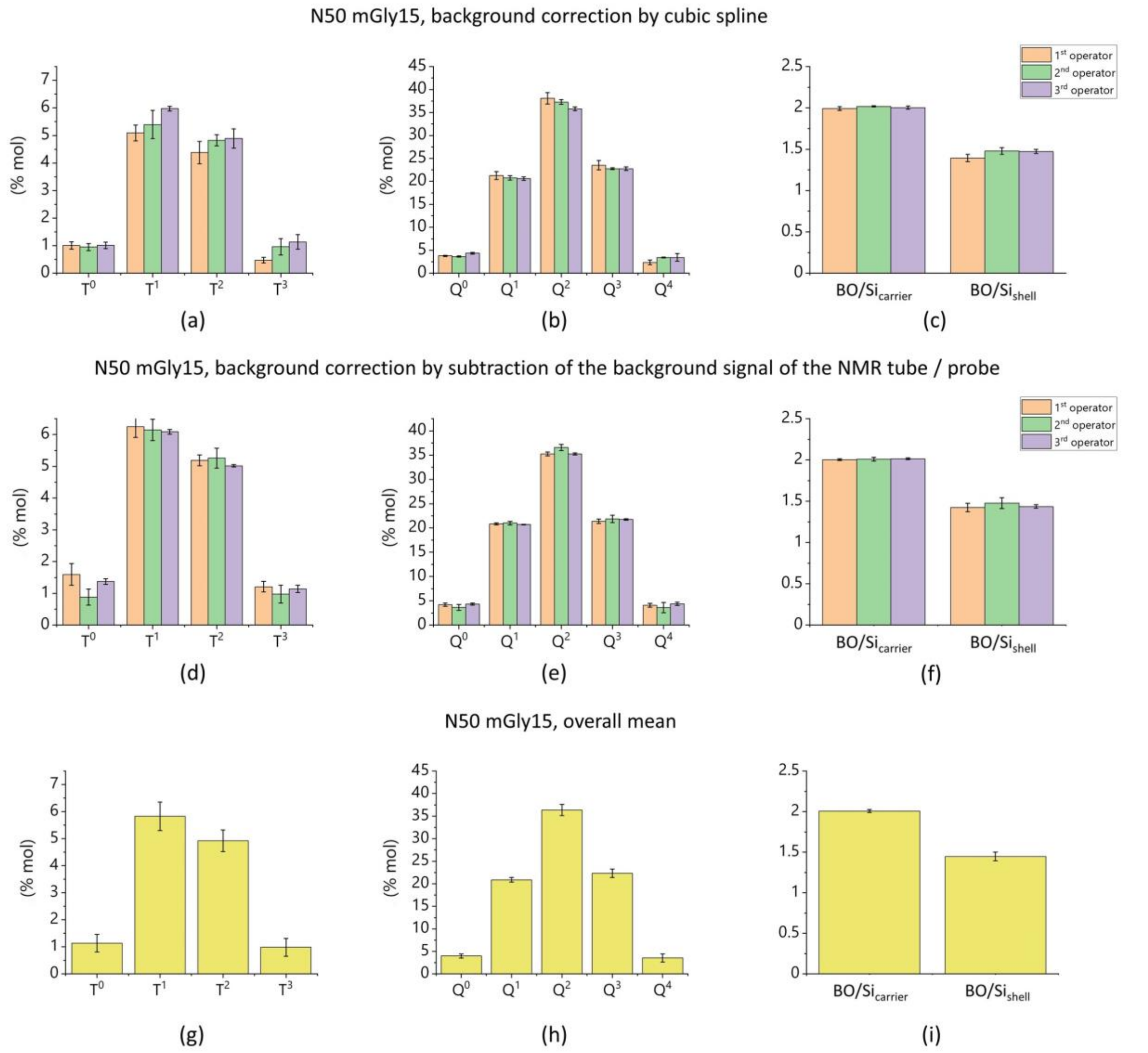

Figure S18: Average relative amounts with standard deviations of (a, d) T, (b, e) Q species, and (c, f) average number of bridging oxygens per Si atom of carrier and shell in sample N50 mGly15. The data processing was performed independently by three different operators, each performing four trials of spline background correction (a-c) or subtraction of the background signal of the NMR tube / probe (d-f). Overall average relative amounts with standard deviations of data are shown in (g) T and (h) Q chemical species, and in (i) $BO/Si_{carrier}$ and BO/Si_{shell} values.

Table S9: Overall mean with standard deviation of the individual chemical species and BO/Si values obtained from the repeated data processing and quantitative analysis of the ^{29}Si NMR data of sample N50 mGly15 (data used for Figure S18 g-i).

Species	Mean (%)	Stdev ± (%)	Relative Stdev ± (%)	BO/Si type	Mean	Stdev ±	Relative Stdev ± (%)
T^0	1.1	0.33	28.7	$BO/Si_{carrier}$	2.01	$BO/Si_{carrier}$	0.94
T^1	5.8	0.53	9.1	BO/Si_{shell}	1.45	BO/Si_{shell}	3.75
T^2	4.9	0.40	8.1				
T^3	1.0	0.33	33.2				

Q^0	4.0	0.44	10.9
Q^1	20.9	0.51	2.5
Q^2	36.4	1.24	3.4
Q^3	22.3	0.93	4.2
Q^4	3.6	0.89	25.0

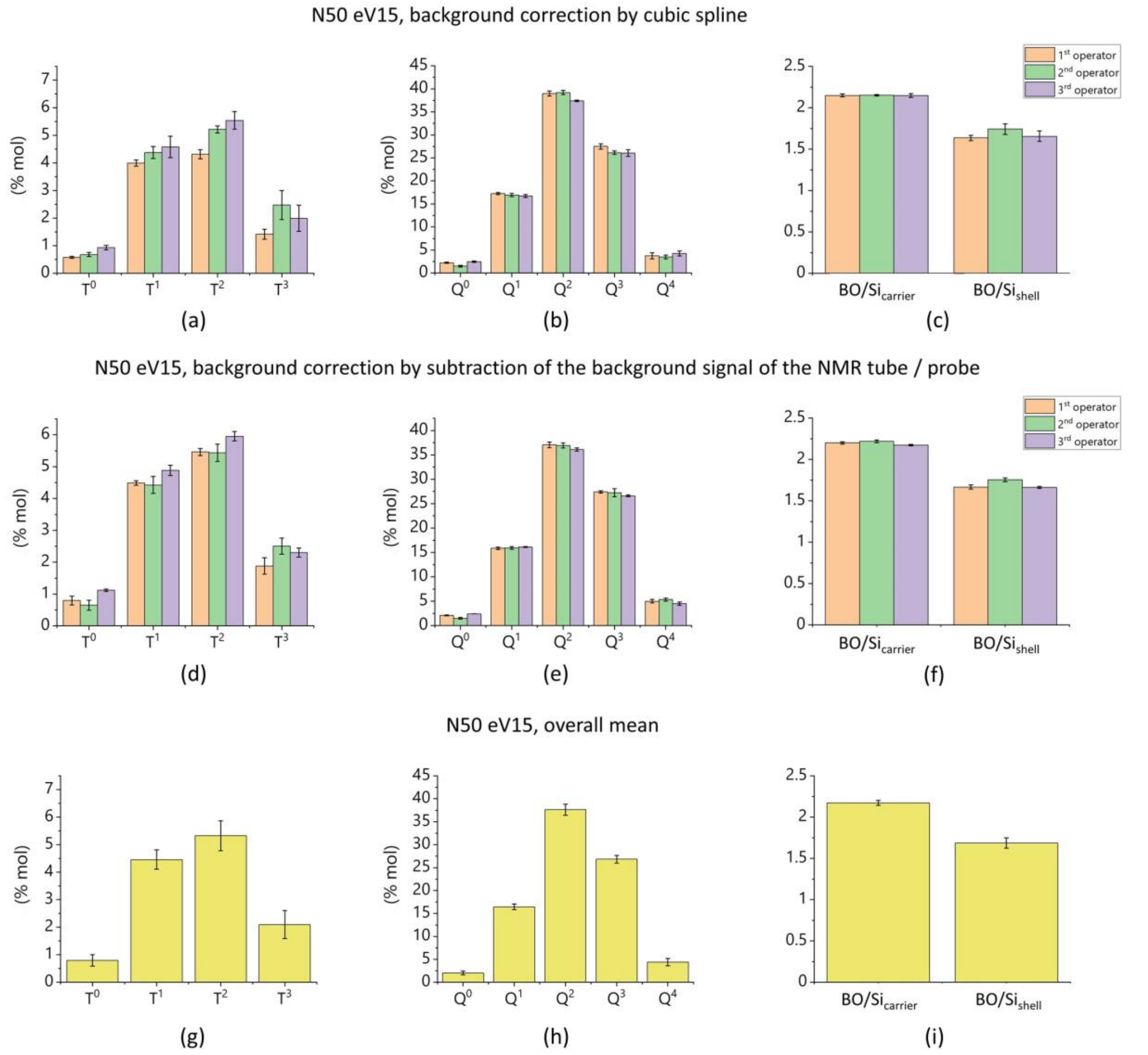

Figure S19: Average relative amounts with standard deviations of (a, d) T, (b, e) Q species, and (c, f) average number of bridging oxygens per Si atom of carrier and shell in sample N50 eV15. The data processing was performed independently by three different operators, each performing four trials of spline background correction (a-c) or subtraction of the background signal of the NMR tube / probe (d-f). Overall average relative amounts with standard deviations of data are shown in (g) T and (h) Q chemical species, and in (i) $BO/Si_{carrier}$ and BO/Si_{shell} values.

Table S10: Overall mean with standard deviation of the individual chemical species and BO/Si values obtained from the repeated data processing and quantitative analysis of the ^{29}Si NMR data of sample N50 eV15 (data used for Figure S19 g-i).

Species	Mean (%)	Stdev ± (%)	Relative Stdev ± (%)	BO/Si type	Mean	Stdev ±	Relative Stdev ± (%)
T^0	0.8	0.21	26.3	$BO/Si_{carrier}$	2.17	0.031	1.44

T^1	4.5	0.35	7.8	BO/Si_{shell}	1.69	0.061	3.63
T^2	5.3	0.54	10.1				
T^3	2.1	0.51	24.2				
Q^0	2.0	0.42	20.6				
Q^1	16.5	0.60	3.6				
Q^2	37.6	1.21	3.2				
Q^3	26.8	0.81	3.0				
Q^4	4.4	0.79	18.1				

Relative standard deviations are generally higher for T species compared to Q species in most cases for each operator as well as for both background correction methods. Since both background corrections result in similar uncertainties and the cubic spline corrections was selected for our standard data interpretation protocol due to its ease of implementation Calculated BO/Si values are in general very consistent, regardless of the operator or the background correction method employed. Generally speaking, low signals of T or Q species cause the biggest difficulties in background correction and integration, since these signals are often slightly above noise and the poor S/N ratio does not allow very accurate evaluation.

5.4 Reproducibility of the synthesis

To study the reproducibility of the funPEOS synthesis, we performed three independent syntheses carried out by three different operators using the same reaction conditions to produce the same N50 ePr15 product. The ^{29}Si NMR spectra of the resulting batches of N50 ePr15 are shown in Figure S20. Each of the three spectra was processed using the cubic spline background correction this time only by one operator. The mean values with standard deviations of the populations of the T and Q chemical species and of the BO/Si values are shown in Figure S21.

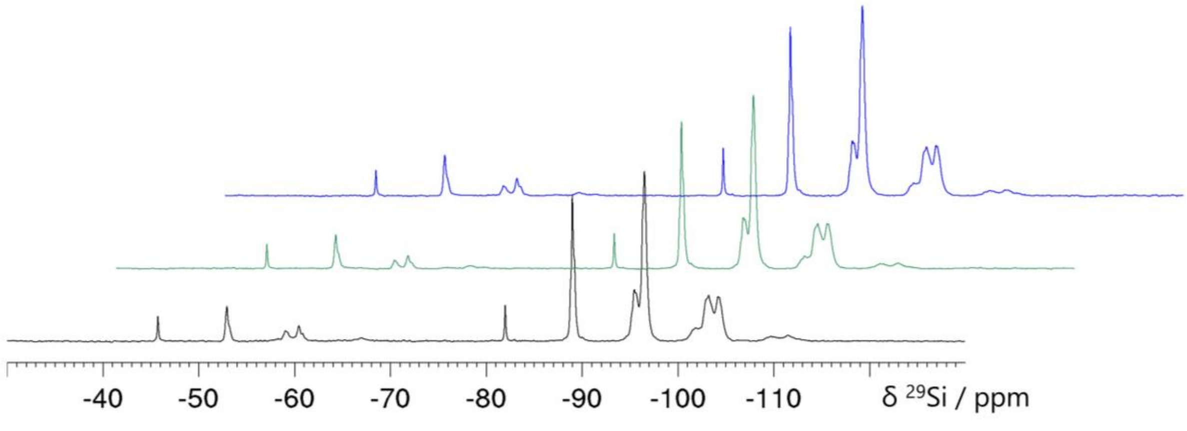

Figure S20: ^{29}Si NMR spectra of three N50 ePr15 products independently prepared under identical conditions by three different operators.

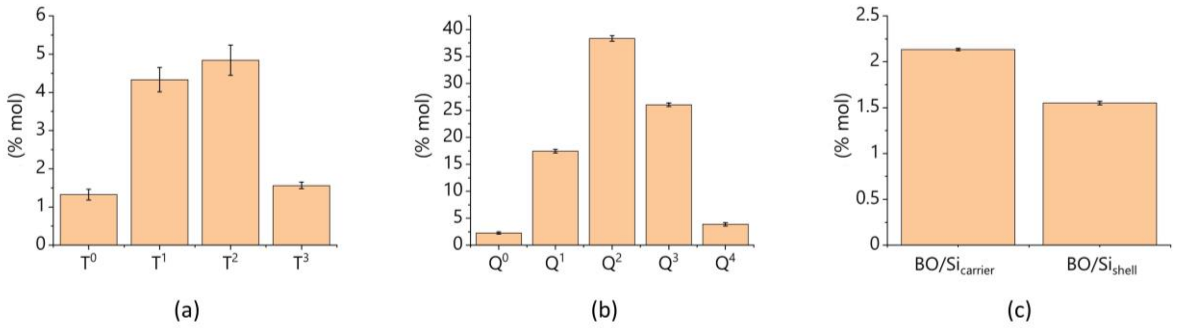

Figure S21: Relative amounts with standard deviations of (a) T and (b) Q chemical species and (c) $BO/Si_{carrier}$ and BO/Si_{shell} values determined by ^{29}Si NMR from three batches of N50 ePr15 synthesized independently under same experimental conditions.

5.5 Relative standard uncertainties of the compositions of the funPEOS samples obtained through ^{29}Si NMR

After analyzing the quantitative results obtained from the evaluation of the ^{29}Si NMR data (Table S8 -Table S10, Figure S17 -Figure S19), we conclude that the relative standard uncertainties vary depending on the type of spectrum processed and the chemical species evaluated. The main effects on the results were observed through differences in the user-dependent subjective nature of the background correction and the choice of integration limits. Both the NMR measurement itself (data acquisition from different NMR samples of the same funPEOS batch, Figure S10) and the funPEOS synthesis (production of several batches of the same material, Figure S20) have a minor influence on the final uncertainty values. For ^{29}Si NMR data repeatedly evaluated by different operators, the scatter around the mean value of the chemical species T^0, T^3, Q^1 and Q^4 is largest (Table S11), which is i) due to their low relative abundance and ii) in the case of e.g. T^3 and Q^4 additionally due to strongly broadened resonances. On the other hand, the standard uncertainty of BO/Si values is remarkably low (Table S11) due to the low impact of the high uncertainty species (T^0, T^3, Q^1 and Q^4) because of their low abundance. Reproducibility as well as repeatability of funPEOS ^{29}Si NMR data evaluation is generally high for well-resolved signals with high abundance (i.e. T^1, T^2, Q^2, and Q^3). However, these rules are not universal and depend on the chemical species present in a final funPEOS formulation (e.g. if higher amounts of T^0 or other species are present in a batch, the scatter around the mean will be lower).

Table S11: Summary of relative standard deviations evaluated by different operators from ^{29}Si NMR data of three different types of funPEOS. The values of the relative standard deviation were obtained from the evaluations in section 5.3 NMR data sets processed and evaluated by different operators (Table S8, Table S9 and Table S10).

	Relative Stdev ± (%) of		
Species or BO/Si type	N50 eM15 (standard alkyl chain type spectrum)	N50 mGly15 (methoxy-ethoxy type spectrum)	N50 eV15 (overlapping signals type spectrum)
T^0	17.0	28.7	26.3
T^1	10.2	9.1	7.8
T^2	12.6	8.1	10.1
T^3	20.4	33.2	24.2

Q^0	19.8	10.9	20.6
Q^1	4.8	2.5	3.6
Q^2	4.1	3.4	3.2
Q^3	3.8	4.2	3.0
Q^4	31.7	25.0	18.1
$BO/Si_{carrier}$	2.09	0.94	1.44
BO/Si_{shell}	3.08	3.75	3.63

6 PEOS/funPEOS specifications

6.1 PEOS synthesis: Q speciation and evolution of Q species

Table S12 shows all the different Q species with the description of their structure, including number of bridging oxygens, number of ethoxy groups and their respective ^{29}Si NMR chemical shifts.

Table S12: Silica speciation in PEOS carrier with their structure, described by their neighboring atoms and their corresponding ^{29}Si NMR shifts (ppm).

Species	Structure	Bridging oxygen	Ethoxy groups	δ ^{29}Si (ppm)
Q^0	TEOS monomer	0	4	-82.0
Q^1	terminal	1	3	-89.0
Q^{2s}	single ring with 4 Si	2	2	-94.6 to -96.1
Q^{2l}	linear/rings with >4 Si	2	2	-96.1 to -97.8
Q^{3d}	double ring with 4 Si	3	1	-100.6 to -102.5
Q^{3s}	single ring with 4 Si	3	1	-102.5 to -104.0
Q^{3l}	linear/ rings with >4 Si	3	1	-104.0 to -105.7
Q^4	dendritic	4	0	-108.5 to -113.4

Figure S22 and Table S13 show evolution of individual Q species over time during PEOS carrier growth. The peaks are labeled as $Q^n(l/m)$ following the notation of Jaumann et. al.[7], where *n* corresponds to the number of bridging oxygens, *l* the number of alkoxy groups and *m* describes the number of acetoxy groups attached to the specific Si atom.

No silanol species were detected in any of the PEOS or funPEOS products (for example Figure 1 or Figure 5 of the main text). As described in Section 11 Dynasylan 40 as a starting material for PEOS synthesis, slanol species typically appear at higher chemical shifts than their non-silanol analogues, the chemical shifts depend on the number of silanol species (for example Q^0 with two silanol groups occur at higher chemical shift compared to Q^0 with one silanol group).

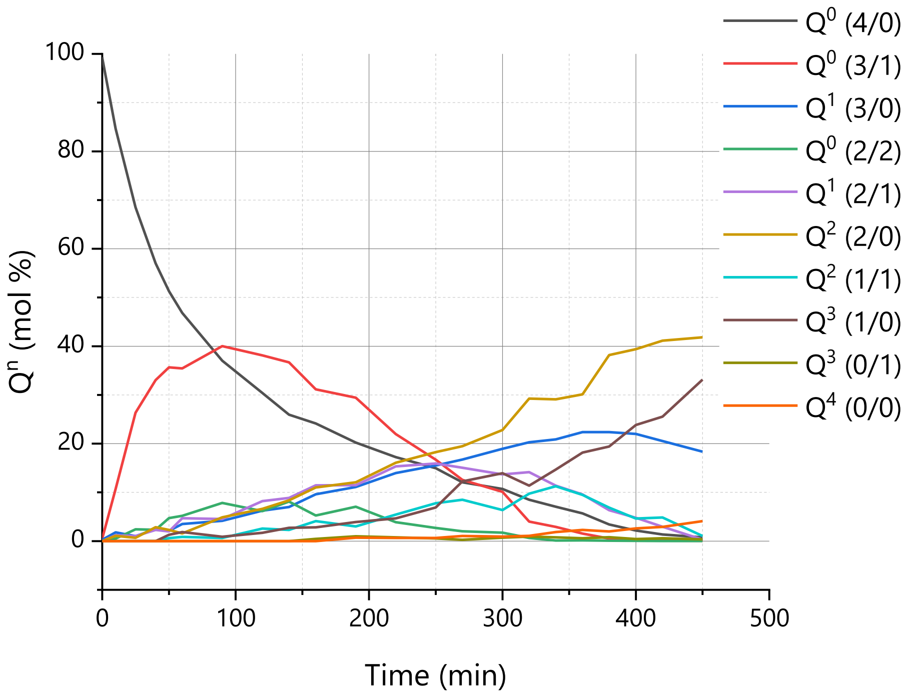

Figure S22: Relative amounts of different Q^n(*l*/*m*) chemical species during the formation of a PEOS carrier over time (data evaluated by ^{29}Si NMR).

Table S13: Relative amounts of different $Q^n(l/m)$ chemical species during the formation of a PEOS carrier over time evaluated by ^{29}Si NMR (raw data to Figure S22).

Time (min)	Q^0 (4/0) (%)	Q^0 (3/1) (%)	Q^1 (3/0) (%)	Q^0 (2/2) (%)	Q^1 (2/1) (%)	Q^2 (2/0) (%)	Q^2 (1/1) (%)	Q^3 (1/0) (%)	Q^3 (0/1) (%)	Q^4 (0/0) (%)
0 (pure TEOS)	99.3	0.0	0.7	0.0	0.0	0.0	0.0	0.0	0.0	0.0
0	99.2	0.5	0.3	0.0	0.0	0.0	0.0	0.0	0.0	0.0
10	84.7	10.7	1.8	0.5	1.2	1.1	0.0	0.0	0.0	0.0
25	68.5	26.4	1.0	2.4	1.0	0.7	0.0	0.0	0.0	0.0
40	57.0	33.1	2.4	2.3	2.3	2.9	0.0	0.0	0.0	0.0
50	51.3	35.7	2.1	4.7	2.0	2.3	0.6	1.3	0.0	0.0
60	46.9	35.4	3.5	5.2	4.7	1.6	0.9	1.9	0.0	0.0
90	37.0	40.0	4.2	7.8	4.5	4.9	0.7	0.9	0.0	0.0
120	30.4	38.1	6.2	6.2	8.2	6.6	2.6	1.7	0.0	0.0
140	26.0	36.7	7.0	8.1	8.9	8.4	2.3	2.7	0.0	0.0
160	24.2	31.2	9.6	5.3	11.4	11.0	4.1	2.8	0.5	0.0
190	20.2	29.4	11.1	7.1	11.5	12.1	3.0	3.9	1.0	0.7
220	17.2	22.0	14.0	3.9	15.3	16.1	5.4	4.7	0.8	0.7
250	15.0	16.7	15.5	2.7	15.9	18.3	7.8	6.9	0.6	0.7
270	12.1	12.6	16.7	2.0	15.1	19.5	8.5	12.3	0.3	1.0
300	10.7	10.1	18.9	1.7	13.7	22.8	6.4	14.0	0.7	0.9
320	8.5	4.0	20.3	0.7	14.2	29.3	9.7	11.4	0.9	1.1
340	7.1	2.9	20.9	0.1	11.4	29.1	11.3	14.7	0.8	1.9
360	5.7	1.6	22.4	0.2	9.6	30.1	9.5	18.2	0.6	2.3
380	3.4	0.5	22.4	0.1	6.4	38.2	6.9	19.4	0.8	2.0
400	2.2	0.2	22.0	0.0	4.8	39.4	4.6	23.8	0.4	2.6
420	1.4	0.1	20.5	0.0	3.0	41.2	4.9	25.6	0.6	2.9
450	0.8	0.0	18.4	0.0	0.3	41.8	1.1	33.1	0.4	4.1

The relative contents of acetylated and non-acetylated species in Figure 3 of the main text and Table S13 were obtained by integrating the individual distinguishable signals of the acquired NMR spectra and normalizing the sum of the peak areas of the acetylated species to the total signal intensity. Mechanistically, the reaction between TEOS and acetic anhydride starts with the formation of acetoxytriethoxysilane, $Q^0(3/1)$, which can react with further acetic anhydride to form diacetoxydiethoxysilane $Q^0(2/2)$ or with TEOS to form a Q^1-Q^1 dimer. Because of the higher reactivity of the first of four ethoxy groups in TEOS, the additional formation of $Q^0(3/1)$ is preferred over the formation of $Q^0(2/2)$ [7,8]. As indicated by the full dataset (Figure 3 a-d main text, Figure S22 and Table S13) and demonstrated earlier [7,8], the condensation of acetoyxtrietoxysilane with alkoxy groups is slower than the acetylation reaction between TEOS and acetic anhydride. As a result, primarily acetylated Q species are formed, which are then reacting by condensation at a slower rate, leading to the asymmetry in the temporal evolution of acetylated versus non-acetylated species (Figure S22 and Figure 3 e of the main text).

6.2 Effect of functional group size on grafting efficiency

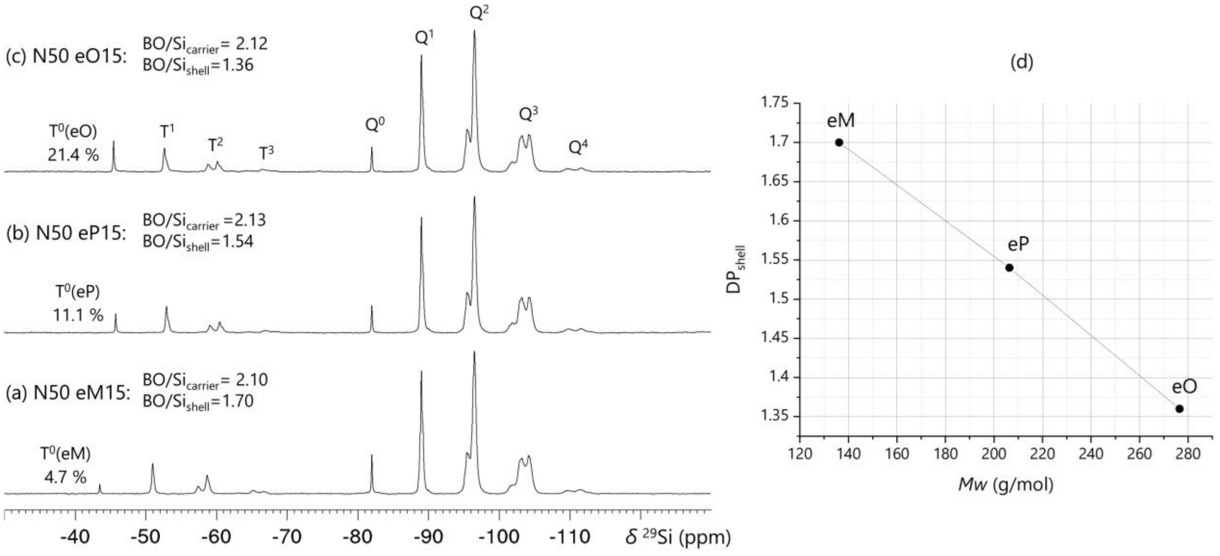

Figure S23: Functional group size effects on T grafting. (a-c) ^{29}Si NMR spectra with obtained BO/Si values and relative T_0 values with respect to the total amount of T species from funPEOS with different alkyl chain lengths (R'): (a) methyl, (b) propyl and (c) octyl group. (d) BO/Si_{shell} as a function of *Mw* of the trialkoxysilane monomers.

The effect of the functional group size on the grafting efficiency was evaluated using organofunctional T-type ethoxysilane model compounds with different alkyl chain lengths: methyl-, propyl- and octyl triethoxysilane. The same PEOS carrier N50, equal molar amounts of trialkoxysilane ($n_{shell}/n_{carrier}$ = 0.15/1) and of acetic anhydride (Ac_2O/Si = 1.5), and identical reaction conditions, (i.e. 140°C, 2 h, 0.4 ml/min addition of acetic anhydride) were used to directly compare the grafting efficiency based on the amounts of non-grafted T^0 monomer and BO/Si_{shell} values of the products. With increasing molecular weight of functional silane starting material, in the ^{29}Si NMR spectra a higher relative T^0 peak intensity of unreacted starting material is observed resulting in lower BO/Si_{shell} values (Figure S23). Note that DF (Equation 4 of the main text), evaluated on the final compound, is nearly constant for all three funPEOS (0.132 for methyl; 0.137 propyl; 0.144 octyl), hence the obtained BO/Si_{shell} values are not strongly affected by the variation in the volatility of the trialkoxy silanes but corresponds to the grafting efficiency.

6.3 Ethoxy-methoxy exchange of funPEOS

In order to demonstrate the ethoxy-methoxy exchange in funPEOS we selected the following example. First 40.6 g of N50 were mixed with 14.5 g of HdTMS, 0.1 ml of TTIP and 4 ml of acetic anhydride (corresponding to Ac_2O/Si ratio 1.00) at 140 °C. The reaction was left to react in the distillation set-up (Figure S3) until no further condensate was produced (70 min). The sample was collected and ^{29}Si NMR was recorded (Figure S24 a). 1 ml of acetic anhydride (corresponding to Ac_2O/Si ratio1.25) together with 0.1 ml of TTIP were added into the reaction mixture. The reaction mixture was left to react until no further condensate was being produced (20 min), a sample was again collected and ^{29}Si NMR spectrum was recorded (Figure S24 b). This step with addition of acetic anhydride and TTIP was repeated for the Ac_2O/Si ratios of 1.50 (addition of 1 ml of AA and 0.1 ml of TTIP, 20 min of reaction time) and 1.75 (addition of 1 ml of AA and 0.1 ml of TTIP, 20 min of reaction time). After each addition, the reaction mixture was left to react for 20 min, which was followed by the sample collection. ^{29}Si NMR spectra of sample corresponding to Ac_2O/Si ratio1.5 and 1.75 are shown in Figure S24 c and d respectively.

Figure S24 shows N50 mHd15 products at different Ac_2O/Si ratios (molar ratio between acetic anhydride and HdTMS) with enlarged view of the range -40 to -50 ppm where T^0 signals with different alkoxy groups are observed.

If the source of Q-type species (carrier) is exclusively TEOS-based and source of T-type species (shell) is based on methoxy species, then transesterification of silicon alkoxy moieties is observed. This leads to obtaining a co-condensate with clearly distinguishable ethoxy and methoxy population on various T-type and Q-type moieties. For a T^0 moiety containing 3 terminal alkoxy groups this means 4 distinguishable subpopulations:

- three –OMe groups (no –OMe has been exchanged) represented by signal at 42.1 ppm
- two –OMe and one –OEt groups (one –OMe has been exchange) represented by signal at 43.2 ppm
- one –OMe and two –OEt groups (two –OMe have been exchanged) represented by signal at 44.4 ppm
- three –OEt groups (three –OMe have been exchanged) represented by signal at 45.5 ppm

Same phenomenon can be observed with Q^0 signals in the range of -79 to -83 ppm. In this case, however, 5 subpopulations can be possibly present due to 4 terminal alkoxy groups that are present in TEOS molecule. In the case of N50 mHd15, only 3 clearly distinguishable subpopulations are visible due to low content of methoxy species.

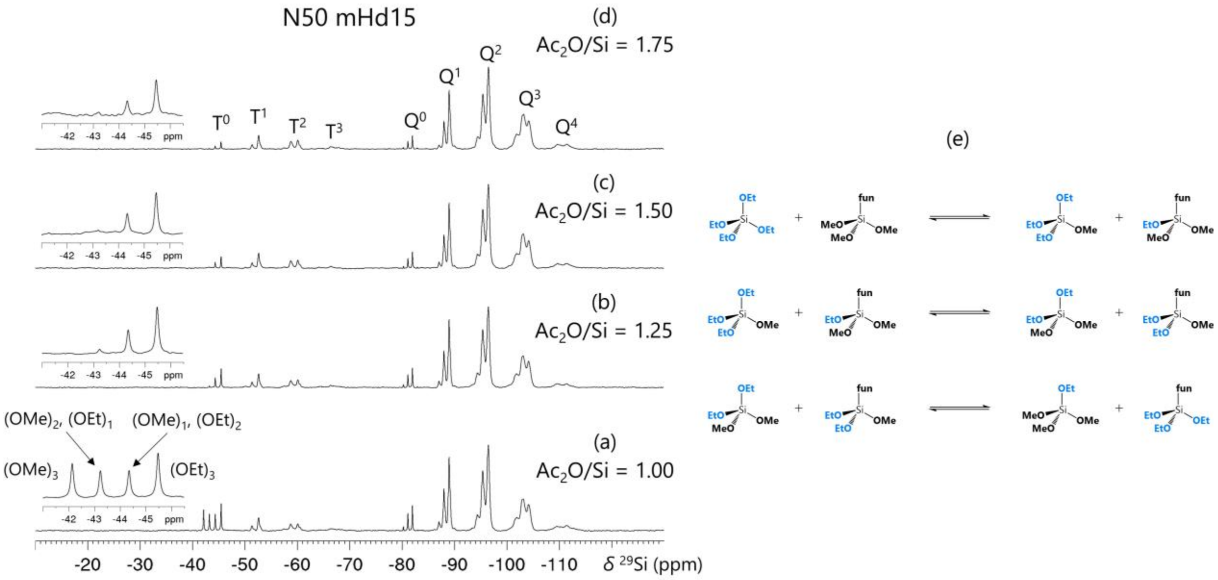

Figure S24: ^{29}Si NMR spectra demonstrating methoxy-ethoxy exchange in a PEOS carrier with BO/$Si_{carrier}$ of 2 (i.e. N50) and hexadecyltrimethoxy ($n_{shell}/n_{carrier}$ = 0.15/1) silane system. The samples were prepared with increasing Ac_2O/Si ratios (*n(acetic anhydride)* / *n(hexadecyltrimethoxysilane))* from (a) 1.0 to (d) 1.75, (e) chemical structures of possible transesterification reaction products (e.g. fun = hexadecyl). The insert in part (a) shows signals assigned to products with different methoxy/ethoxy combinations.

The effect of alkoxy type of functional T silane on the grafting efficiency was evaluated using propyltriethoxysilane (PTES) vs propyltrimethoxysilane (PTMS) systems. The same PEOS carrier N50, equal molar amounts of trialkoxysilane ($n_{shell}/n_{carrier}$ = 0.12/1) and of acetic anhydride (Ac_2O/Si = 1.5), and identical reaction conditions, (i.e. 140°C, 2 h, 0.4 ml/min addition of acetic anhydride) were used to directly compare the grafting efficiency based on the amounts of non-grafted T^0 monomer and BO/Si_{shell} values of the products. Figure S25 shows ^{29}Si NMR spectra recorded from N50 carrier functionalized with (a) PTES (eP) and (b) PTMS (mP). NMR analysis shows that N50 ePr12 exhibits BO/Si_{shell} of 1.43 and contains around 1.4% by mol of T^0 while N50 mPr12 exhibits higher BO/Si_{shell} of 1.70 and contains only around 0.5% by mol of T^0.

With bulkier alkoxy groups of functional silane starting material, poorer grafting efficiency is obtained for the same reaction conditions when compared to sterically smaller alkoxy groups. Selecting methoxy-terminated functional silanes leads to better conversion and a higher BO/Si_{shell} and is particularly recommended for functional silanes with sterically more demanding functional groups such as octyl or isobutyl.

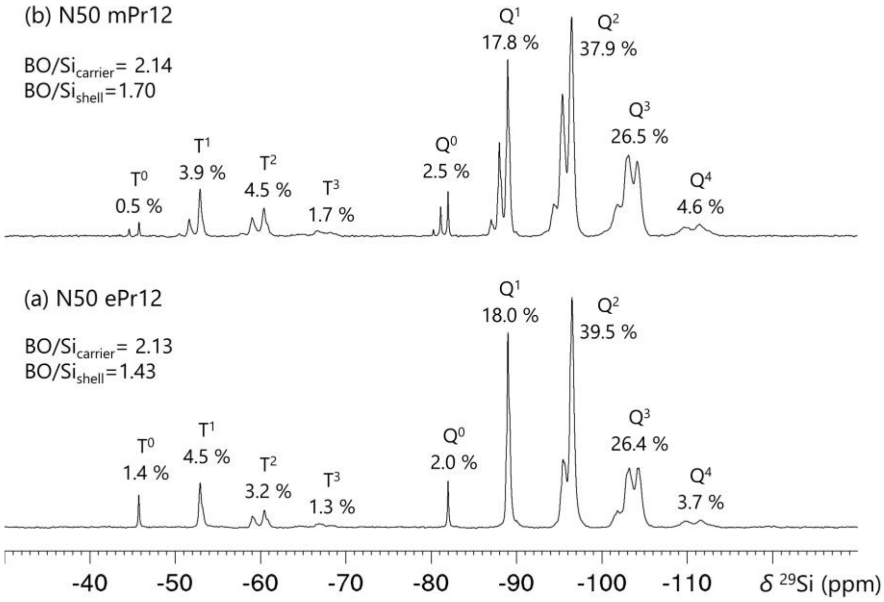

Figure S25: ^{29}Si NMR spectra recorded from N50 carrier functionalized with (a) propyltriethoxysilane (eP) and (b) propyltrimethoxysilane (mP) synthesized under the same experimental conditions (same Ac_2O/Si ratio, speed of acetic anhydride addition, TTIP concentration, time of reaction, purification procedure and parameters).

6.4 Mass balance

As a representative case for the mass balance, we have selected the preparation of the PEOS substrate and subsequent functionalisation of said substrate with methyltriethoxysilane (MTES). Starting with 4000 g of TEOS and a theoretical BO/$Si_{carrier}$ value of 2.0, 3700 g of condensate were collected, which is about 384 g more than the theoretical amount of ethyl acetate according to stoichiometry (Table S13). However, also approximately 375 g less product was collected than expected. This suggests, that in this case the condensate consists not only of ethyl acetate, but also contains reactants introduced to the reaction, i.e. MTES with its boiling point (142°C) close to the reaction temperature (140 °C). This hypothesis was further corroborated in a second small-scale experiment, where octyltriethoxysilane (OTES) was grafted in the second step: now, the mass balance accounted for over 95 % of condensate and >98 % of the theoretical yield, presumably because there were no losses of the OTES monomer with a boiling point of 224 °C (Table S13 and S14).

Above mentioned alkyl-functionalized siloxane products (large-scale production of N50 eM30 and small-scale production of N50 eO30 formulations) as well as the collected distillates during these syntheses produced by quantitative reaction (Scheme 1 of the main text) presented in Table S14 were subjected to quantitative ^{29}Si NMR data evaluation to study efficiency of functional-monomeric silane grafting onto PEOS structure to prove the stoichiometric nature of this reaction. Collected condensates were doped with 5 and 10 mass % of DMDES to obtain molar amount of collected MTES and OTES in the condensates by quantitative NMR analysis. DMDES was selected as a standard because its NMR signal is significantly shifted from potential Q and T signals typically present in N50 eM30 and N50 eO30 and 5 and 10°% were used to have 2 data points instead of 1. As was already pointed out in the main text, apart from acquiring high conversion of monomeric functional silanes by attaching them onto PEOS carrier, it is also important to consider boiling point of individual T, D or M silanes and reaction temperature. Table S15 shows two examples of methyl and octyl functionalized PEOS with boiling points of 142 °C and 224 °C respectively. Condensate from N50 eM30 synthesis contains approximately 3340 mmol of MTES which makes up for 40.4 % of initially used MTES whereas N50 eO30 condensate contains 0 % of monomeric OTES (Table S15), leading to a reduction of $n_{shell}/n_{carrier}$ from the nominal 0.30 ($n_{shell}/n_{\mathrm{carrier}}$ theoretical) to an actual value of 0.18 ($n_{shell}/n_{carrier}$ final) which corresponds to $n_{shell}/n_{carrier}$ calculated from obtained ^{29}Si NMR ($n_{shell}/n_{carrier}$ NMR calculated, DF from Equation 4 in the main text) of condensate collecting during N50 eM30 synthesis (Table S16).

Table S14: mass balance of two funPEOS syntheses: N50 carrier ($BO/Si_{carrier}$ = 2) functionalized with functional silane (fun) MTES and N50 carrier with OTES.

Carrier Synthesis			
funPEOS		N50 eM30	N50 eO30
m(D40)	(g)	4000	56
n(Si)	(mol)	24.47	0.38
Ac_2O/Si ratio		1	1
n(AA)	(mol)	4.4	0.06
m(AA)	(g)	448.8	6.3
Shell synthesis			
fun silane		MTES	OTES
n(fun)	(mol)	8.2	0.1
m(fun)	(g)	1469.5	31.9
Ac_2O/Siratio		1.8	1.5
n(AA)	(mol)	14.4	0.2
m(AA)	(g)	1472.6	17.7
m(EtOAc) theory	(g)	3316.2	41.3
m(Condensate) collected	(g)	3700	42.3
m(funPEOS) theory	(g)	4074.7	70.5
m(funPEOS) collected	(g)	3700	66.7

Table S15: Content of monomeric MTES and OTES in collected condensate during N50 eM30 and N50 eO30 syntheses respectively, obtained by quantitative ^{29}Si NMR analysis.

funPEOS		Mass ratio (%)	Amount in 1 g of condensate (mmol)	Mass of condensate (g)	Amount in collected condensate (mol)	Average amount in collected condensate (mol)
N50 eM30 synthesis	condensate	95		3700		3.340
	DMDES	5	0.32			
	MTES		0.93		3.450	
	condensate	90		3700		
	DMDES	10	0.63			
	MTES		0.87		3230	
N50 eO30 synthesis		No OTES signal in ^{29}Si NMR spectrum of condensate collected form N50 eO30 synthesis			0	0

Table S16: Overview of graftability of MTES and OTES onto PEOS carrier at 140 °C and changes in $n_{shell}/n_{\text{carrier}}$ due to loss of monomeric functional silane.

	N50 eM30	N50 eO30
% of grafted func silane	59.6	100.0
% of func silane in condensate	40.4	0.0
$n_{shell}/n_{\text{carrier}}$ theoretical	1/0.30	1/0.30
$n_{shell}/n_{carrier}$ final	1/0.18	1/0.30
$n_{shell}/n_{carrier}$ NMR calculated (DF)	1/0.18	1/0.26

7 PEOS/funPEOS characterization

All the synthesized funPEOS samples were analyzed by ^{1}H, ^{13}C (data in Figure S26 - Figure S43), ^{29}Si NMR (Figure 5, main text) and FTIR (Figure S44 - Figure S53). Abbreviations used in the spectra: EtAc and MeAc = ethyl and methyl acetate, EtOH = ethanol and $CDCl_3$ = deuterated chloroform.

7.1 ^{1}H and ^{13}C NMR of synthesized funPEOS

The NMR spectra in Figure S26 - Figure S43 are all shown in the following order of compounds:

- Top: funPEOS product
- Middle: PEOS carrier
- Bottom: organofunctional trialkoxysilane monomer R'-Si(OR)$_3$

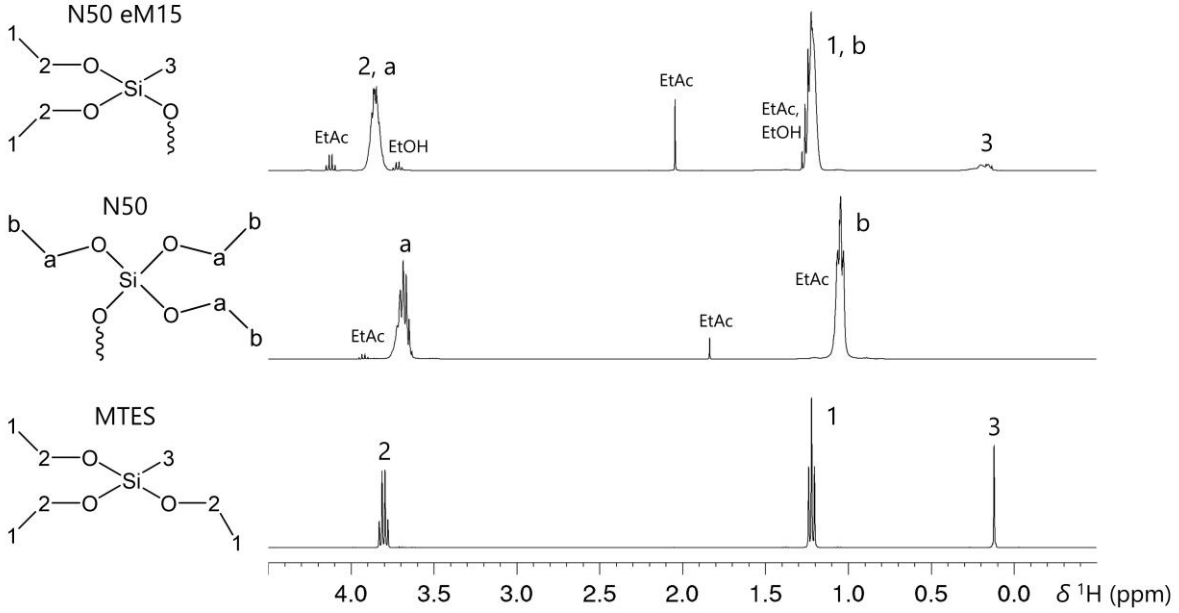

Figure S26: Region of interest of ^{1}H NMR spectra of sample N50 eM15, N50 and MTES monomer (CDCl$_3$).

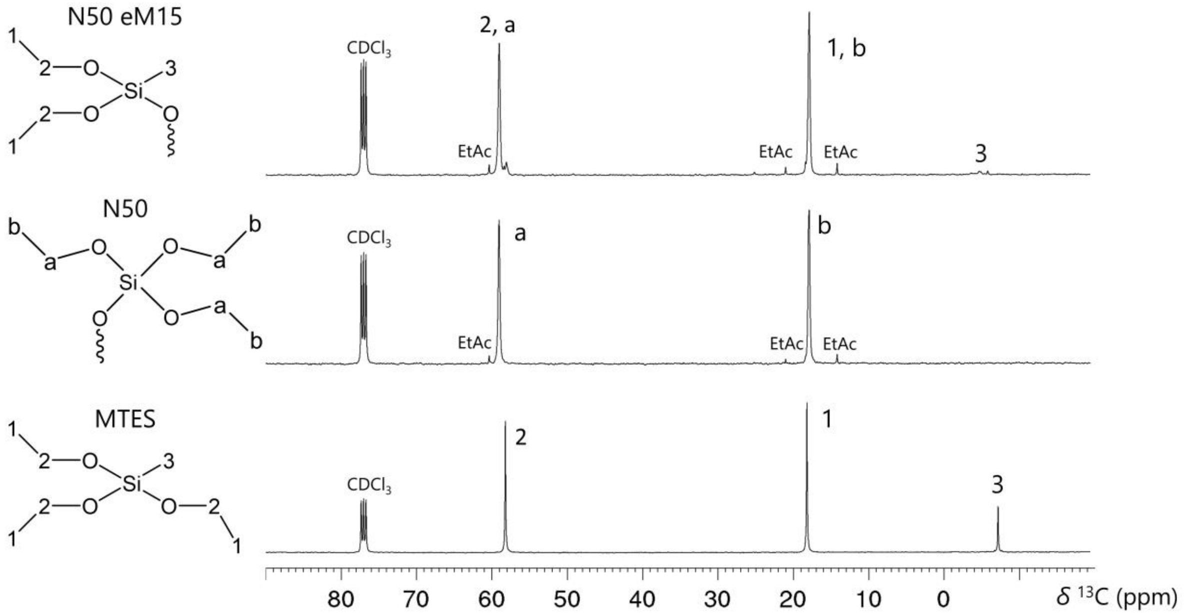

Figure S27: Region of interest of ^{13}C NMR spectra of sample N50 eM15, N50 and MTES monomer (CDCl$_3$).

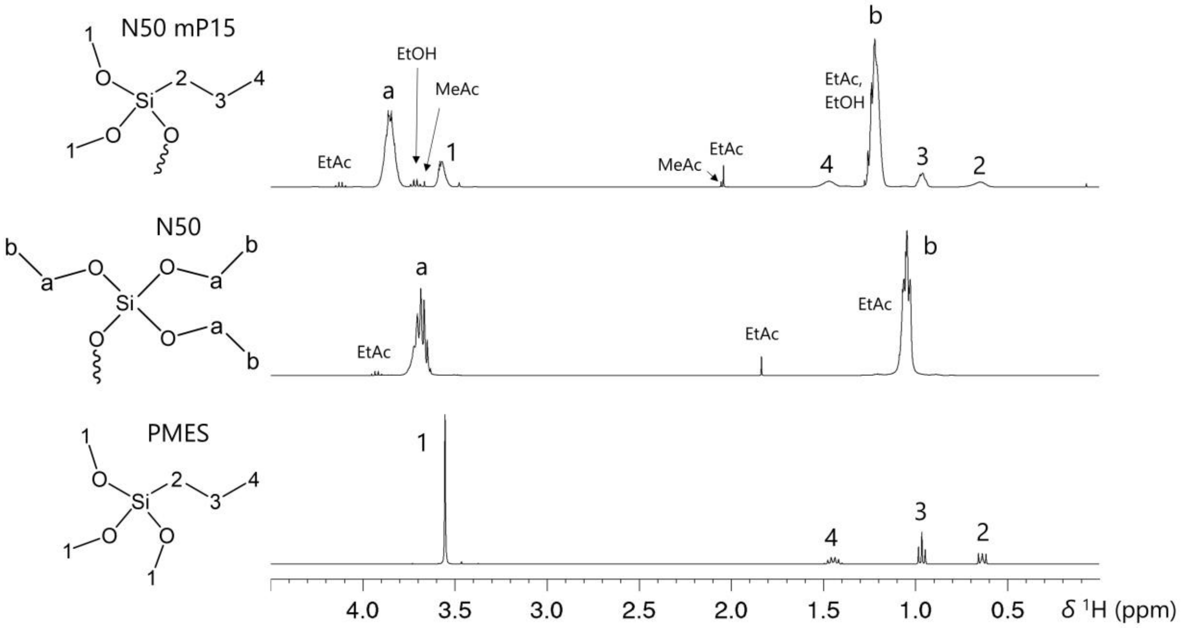

Figure S28: Region of interest of ^{1}H NMR spectra of sample N50 mP15, N50 and PMES monomer ($CDCl_3$).

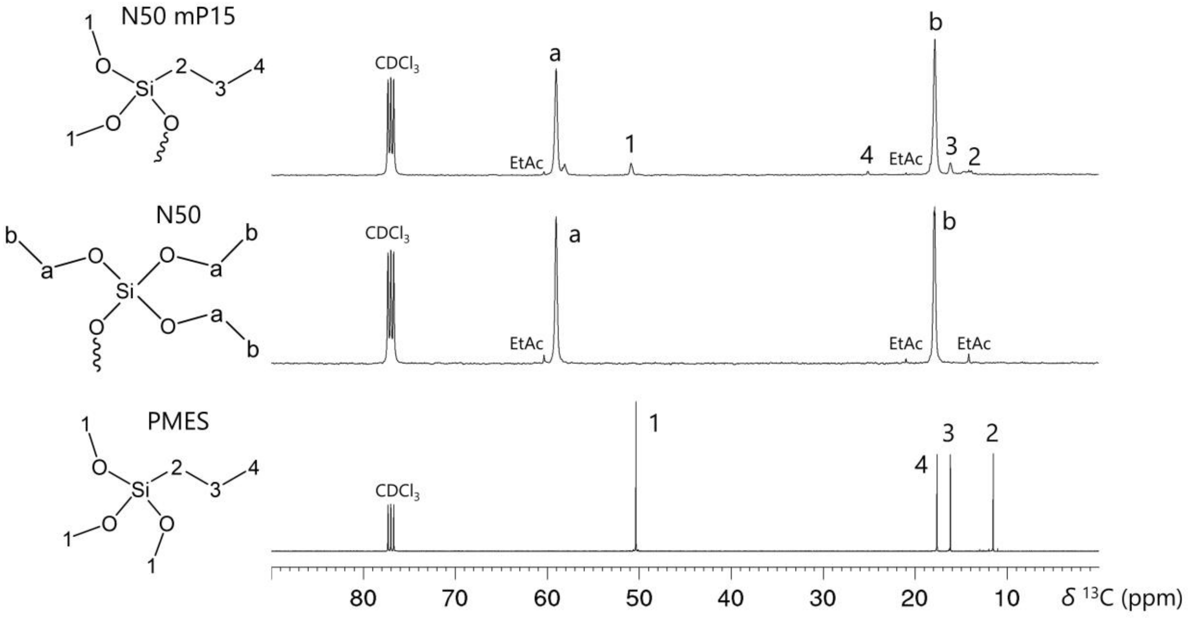

Figure S29: Region of interest of ^{13}C NMR spectra of sample N50 mP15, N50 and PMES monomer ($CDCl_3$).

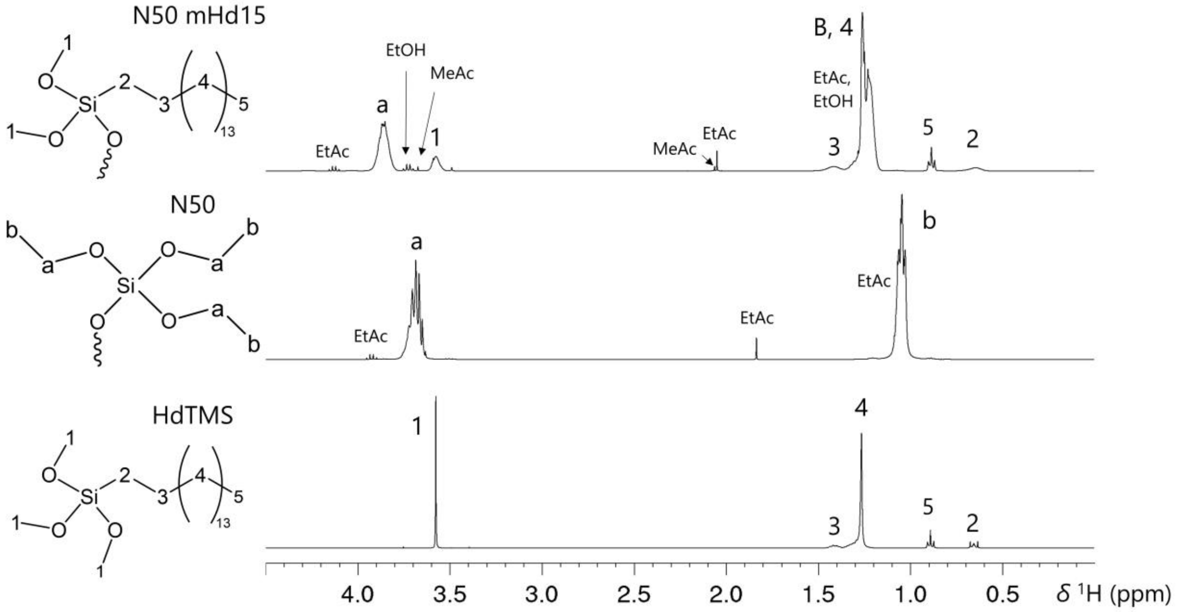

Figure S30: Region of interest of ^{1}H NMR spectra of sample N50 mHd15, N50 and HdTMS monomer ($CDCl_3$).

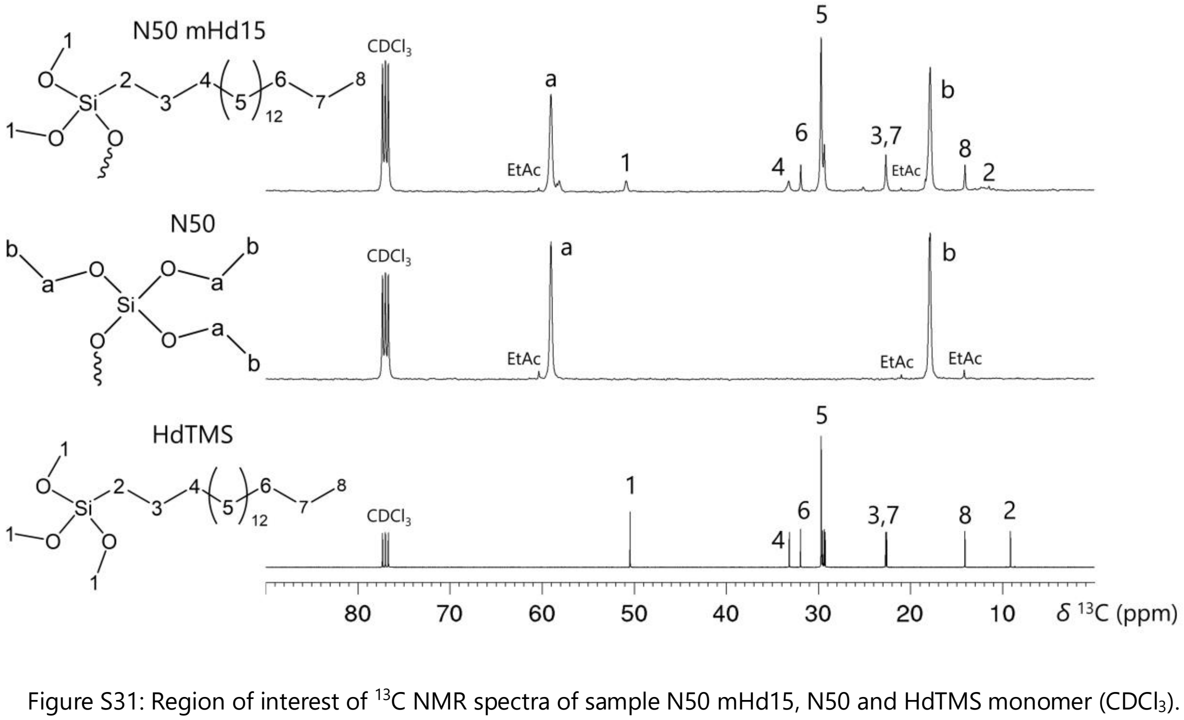

Figure S31: Region of interest of ^{13}C NMR spectra of sample N50 mHd15, N50 and HdTMS monomer ($CDCl_3$).

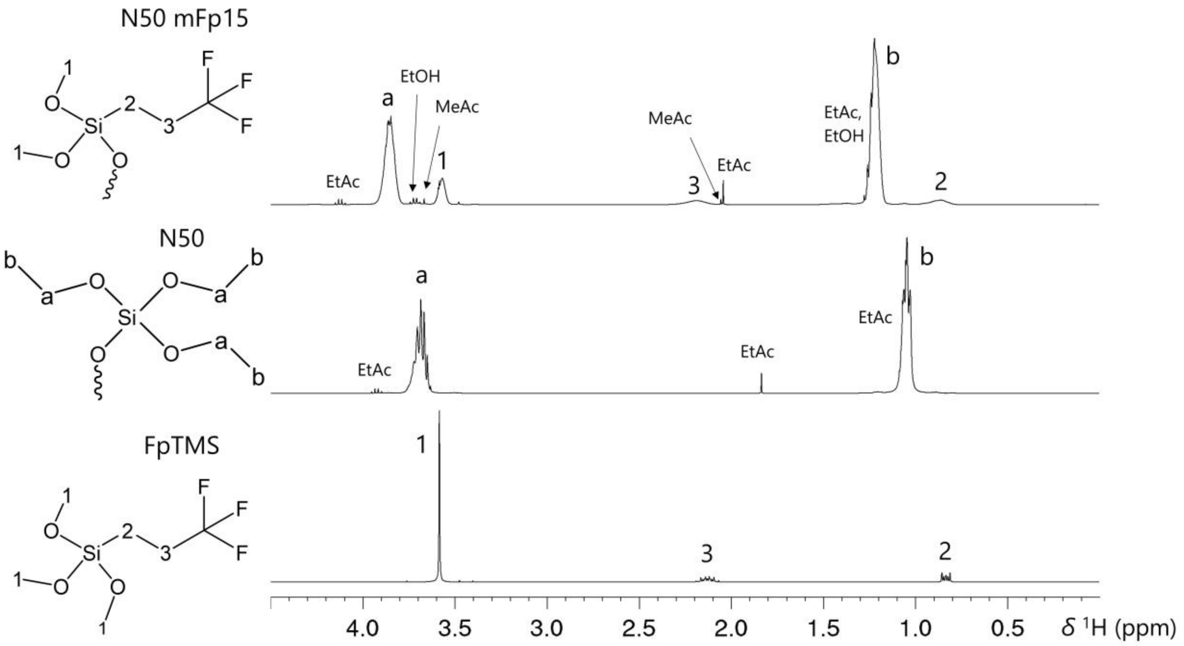

Figure S32: Region of interest of ^{1}H NMR spectra of sample N50 mFp15, N50 and FpTMS ($CDCl_3$).

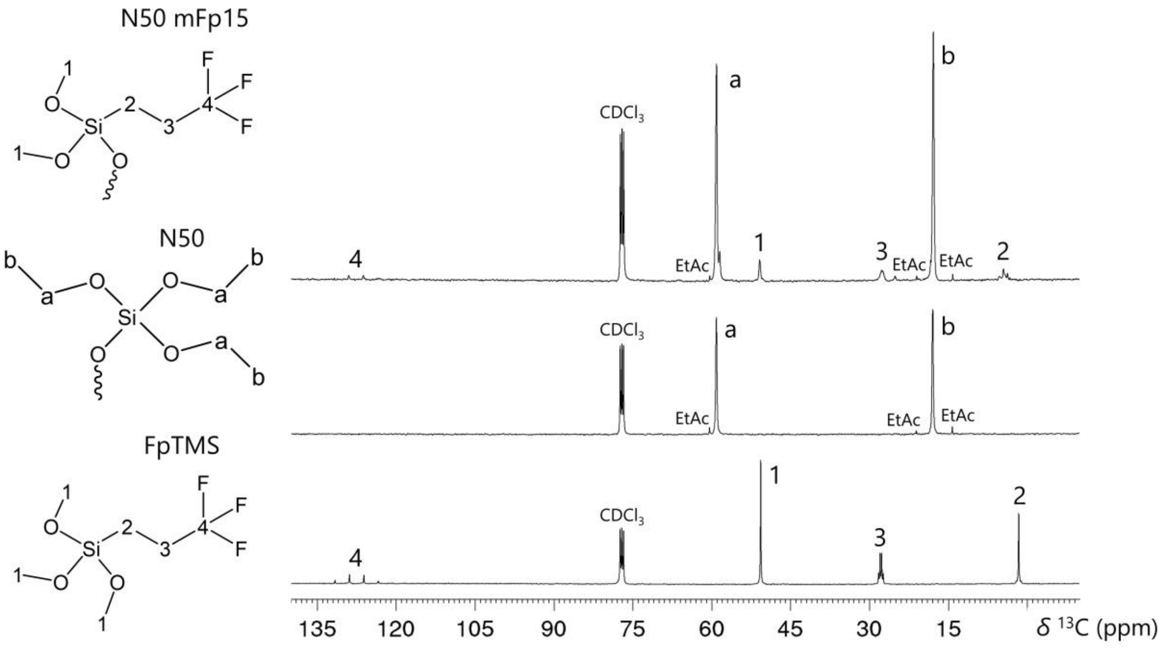

Figure S33: Region of interest of ^{13}C NMR spectra of sample N50 mFp15, N50 and FpTMS ($CDCl_3$).

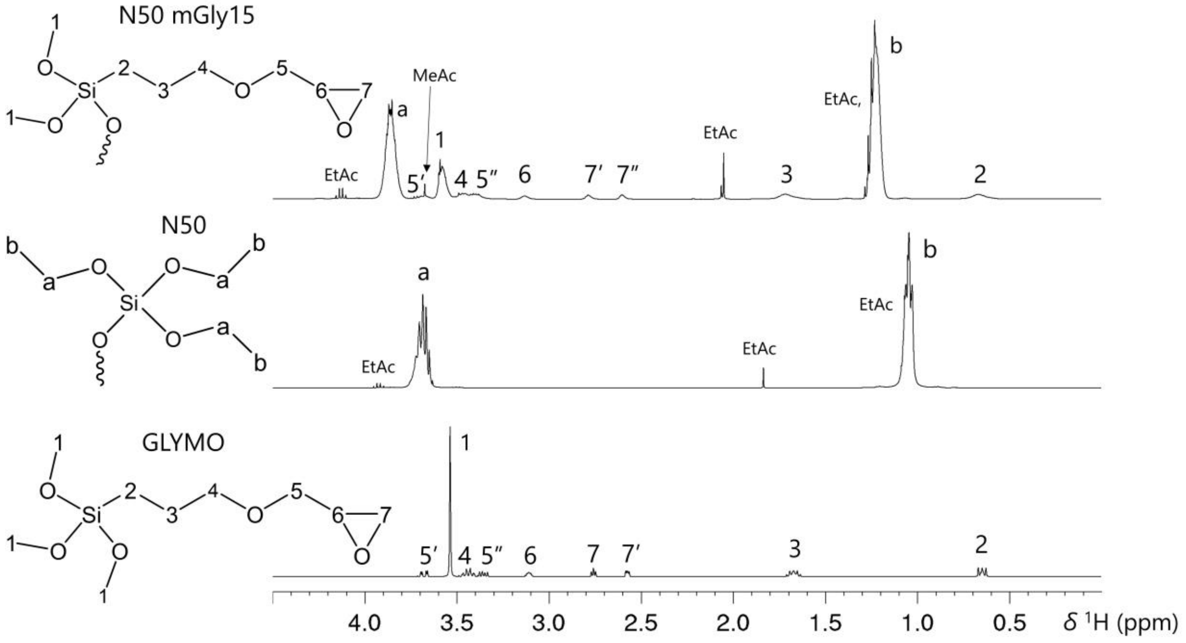

Figure S34: Region of interest of ^{1}H NMR spectra of sample N50 mGly15, N50 and GLYMO monomer ($CDCl_3$).

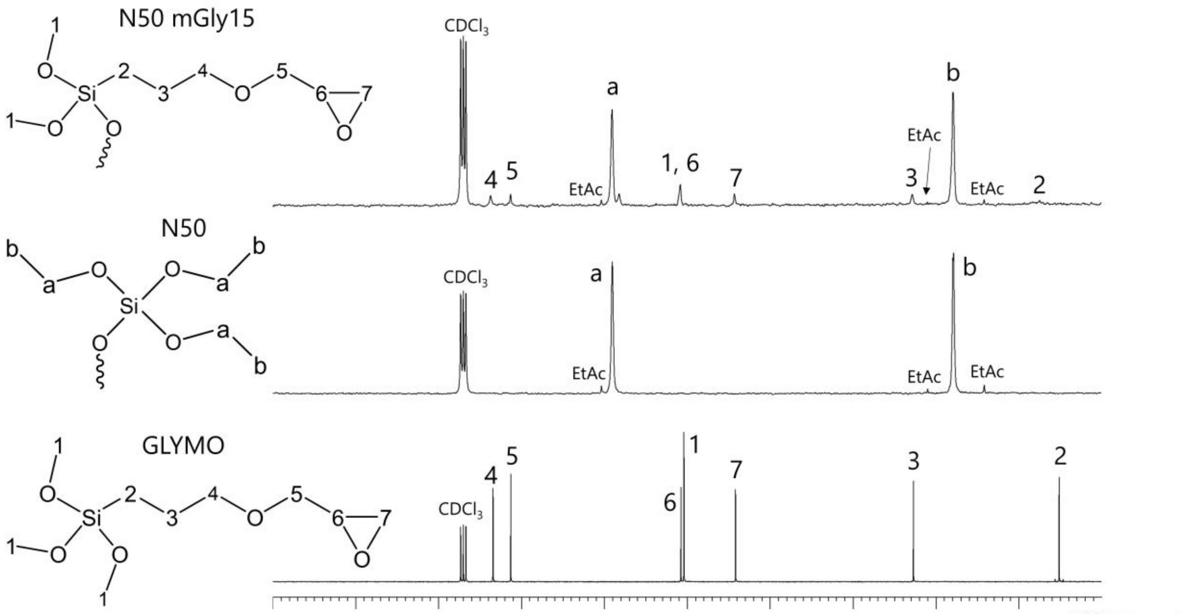

Figure S35: Region of interest of ^{13}C NMR spectra of sample N50 mGly15, N50 and GLYMO monomer[9] ($CDCl_3$).

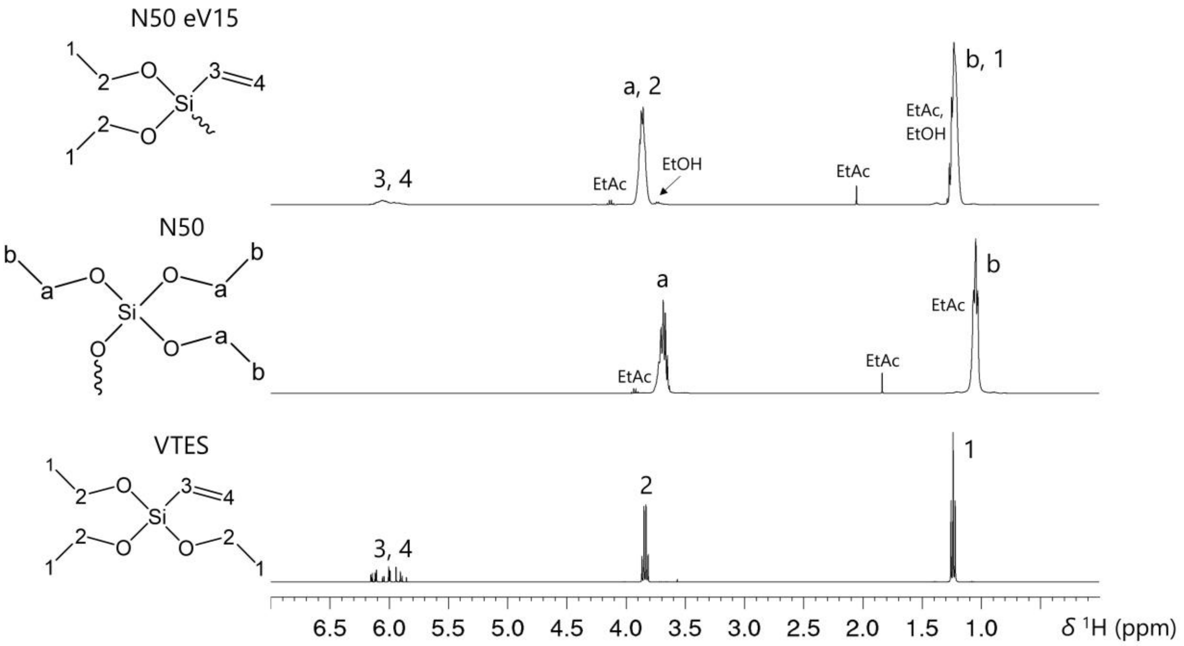

Figure S36: Region of interest of ^{1}H NMR spectra of sample N50 eV15, N50 and VTES monomer ($CDCl_3$).

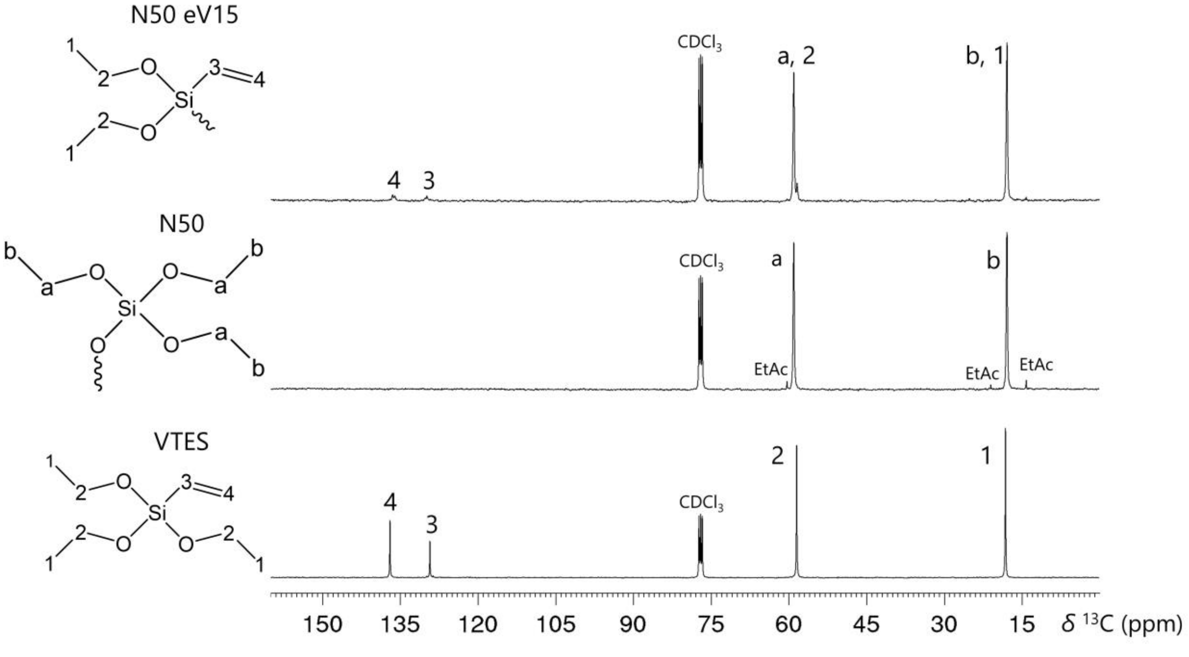

Figure S37: Region of interest of ^{13}C NMR spectra of sample N50 eV15, N50 and VTES monomer ($CDCl_3$).

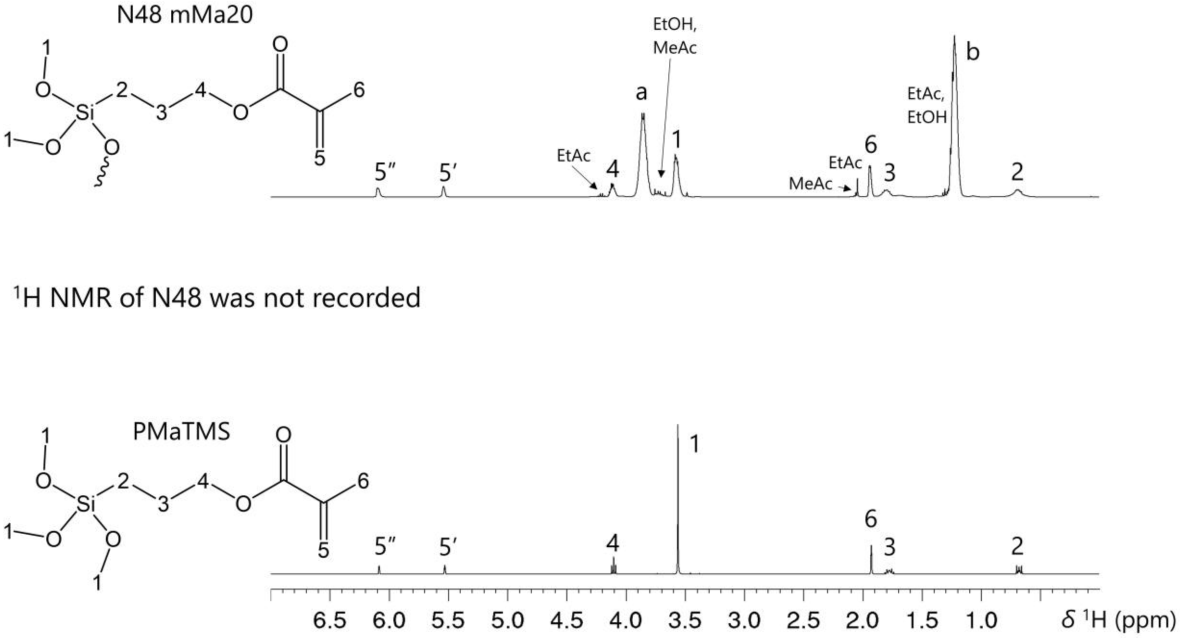

Figure S38: Region of interest of ^{1}H NMR spectra of sample N48 mMa20 and PMaTMS ($CDCl_3$).

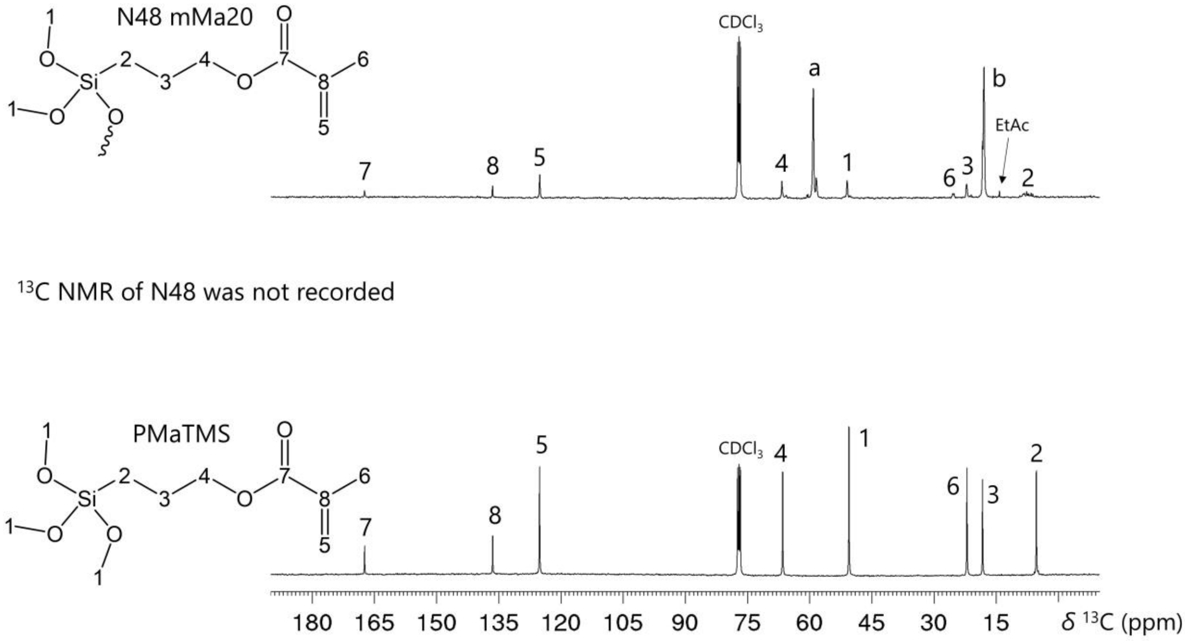

Figure S39: Region of interest of ^{13}C NMR spectra of sample N48 mMa20 and PMaTMS ($CDCl_3$).

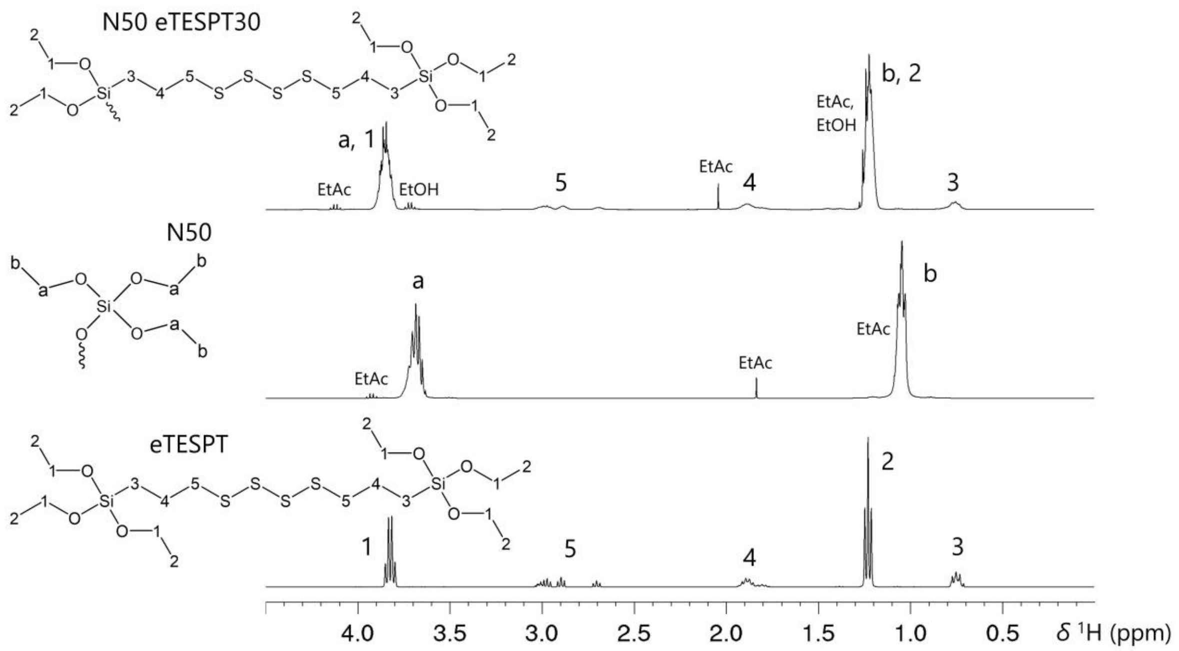

Figure S40: Region of interest of ^{1}H NMR spectra of sample N50 eTESPT15, N50 and eTESPT ($CDCl_3$).

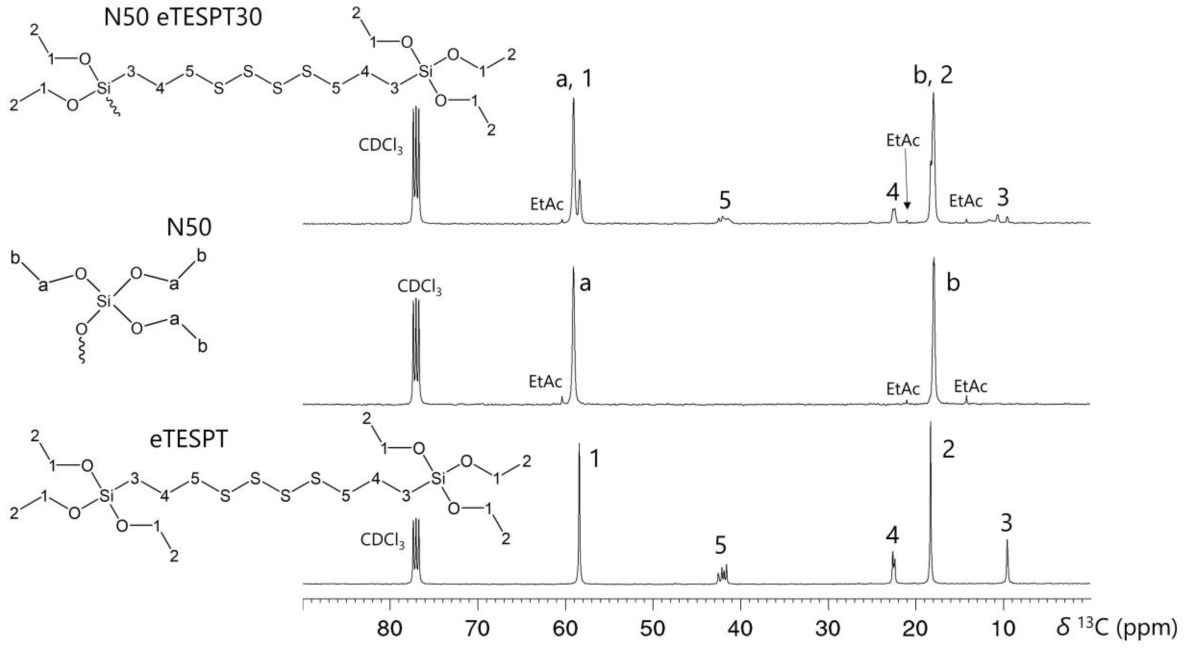

Figure S41: Region of interest of ^{13}C NMR spectra of sample N50 eTESPT15, N50 and eTESPT ($CDCl_3$).

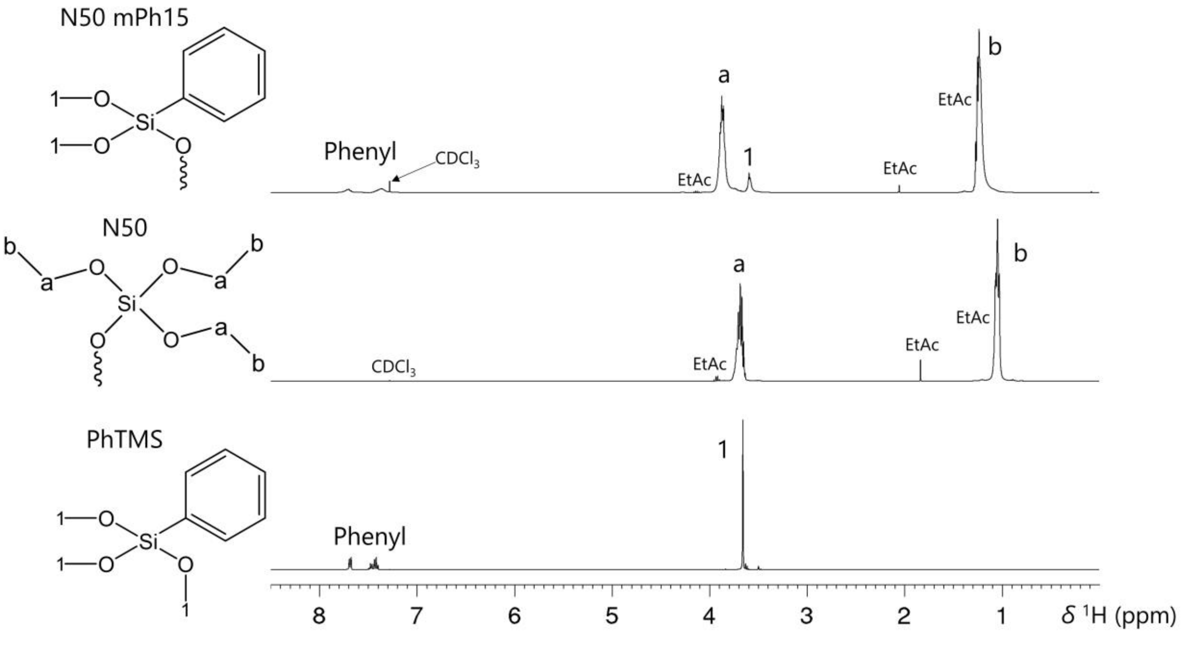

Figure S42: Region of interest of ^{1}H NMR spectra of sample N50 mPh15, N50 and PhTMS ($CDCl_3$).

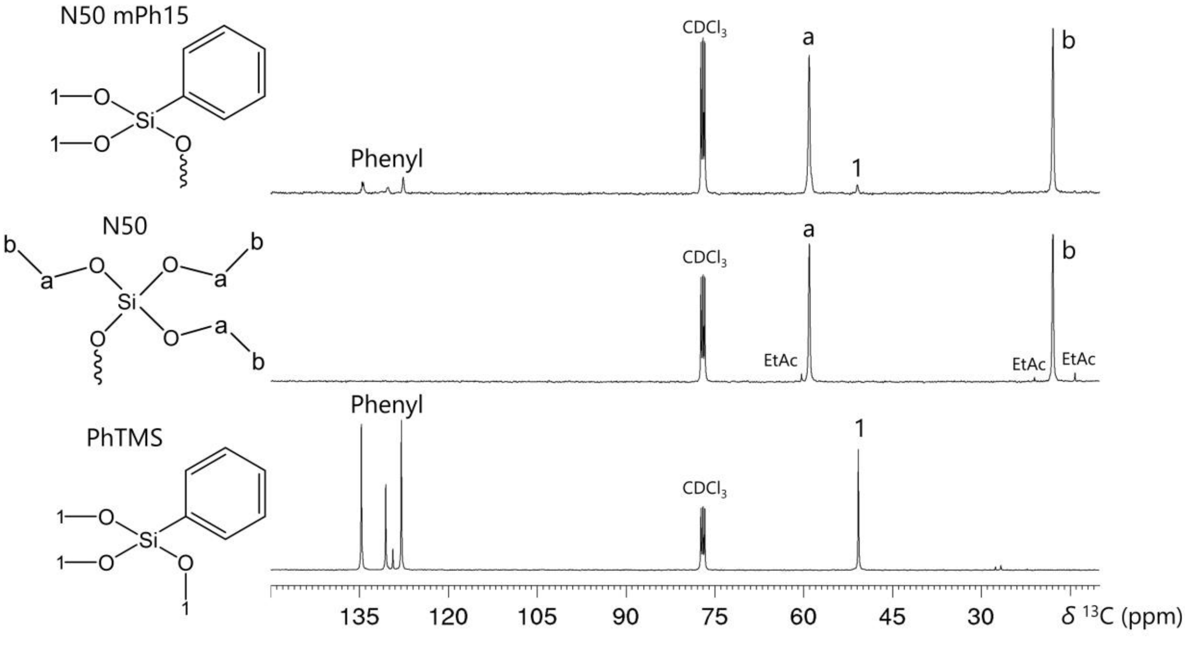

Figure S43: Region of interest of ^{13}C NMR spectra of sample N50 mPh15, N50 and PhTMS ($CDCl_3$).

7.2 FTIR characterization

The FTIR analysis of the funPEOS compounds support the presence of the chemical structures of here presented molecules. In the following section, the IR spectrum of each compound is displayed with a small description. Since the functionalization of the compounds is in the range of 15 to 20 %, some of the IR bands are quite weak because of the low concentration of the analytes. Firstly, individual signals in the pure Q-type polysiloxane (PEOS substrate) are assigned Figure S44. The spectrum of each functionalized polysiloxane is displayed in comparison with this Q type polysiloxane and the functional monomer used for functionalization (Figure S45 - Figure S53).

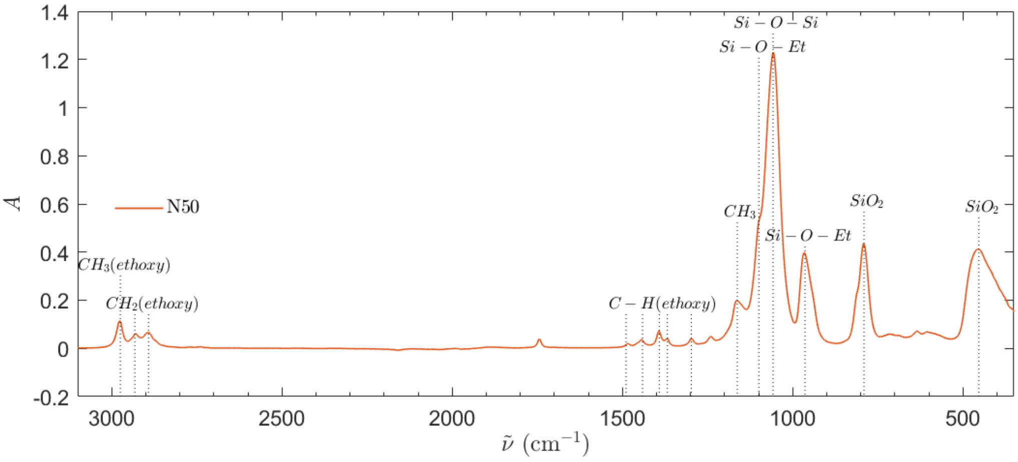

Figure S44: FTIR spectrum of N50 (pure Q-type polysiloxane). Peaks at 1250 and 1750 cm^{-1} are from the ethyl acetate by-product [10–12].

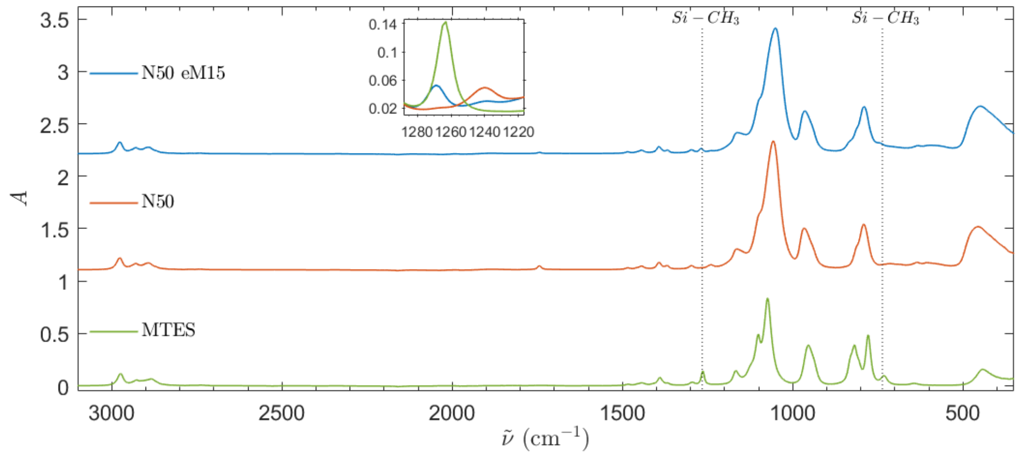

Figure S45: FTIR spectra of N50 eM15, N50 and MTES with enlarged view of the range 1220-1280 cm^{-1}.

The signals of the methyl group in Figure S45 are observed at 1265 and 740 cm^{-1}. The strong peak at 820 cm^{-1} in the MTES spectrum comes from the monomer and is barely visible in the functionalized polysiloxane[10].

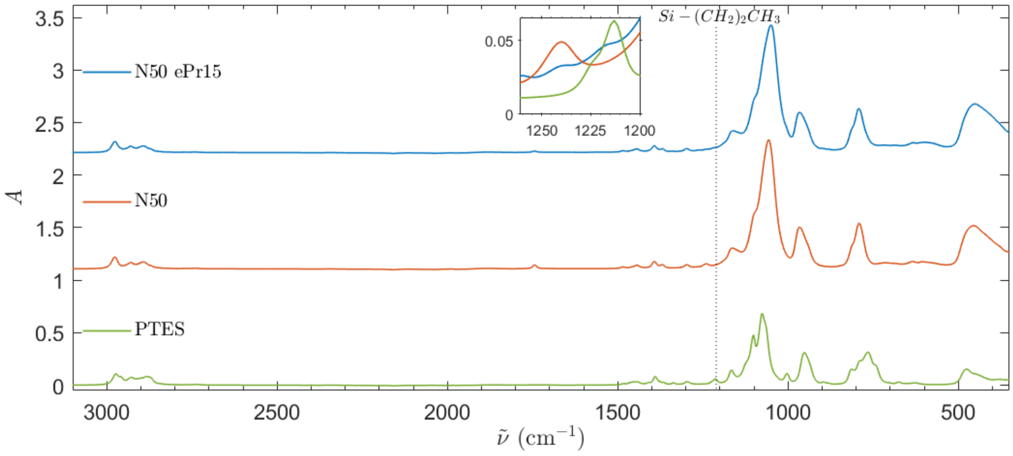

Figure S46: FTIR spectra of N50 ePr15, N50 and PTES with enlarged view of the range 1200-1250 cm^{-1}.

The rather weak band visible at 1215 cm^{-1} in Figure S46 can be assigned to the Si-(CH_2) bond. It is comparable to the signal of Si-CH_3, but since here we look at longer alkyl chain it is moved towards lower wavenumbers and is masked by the strong signal from polysiloxane. The peak at 750 cm^{-1} is slightly broadened because of the propyl chain[11].

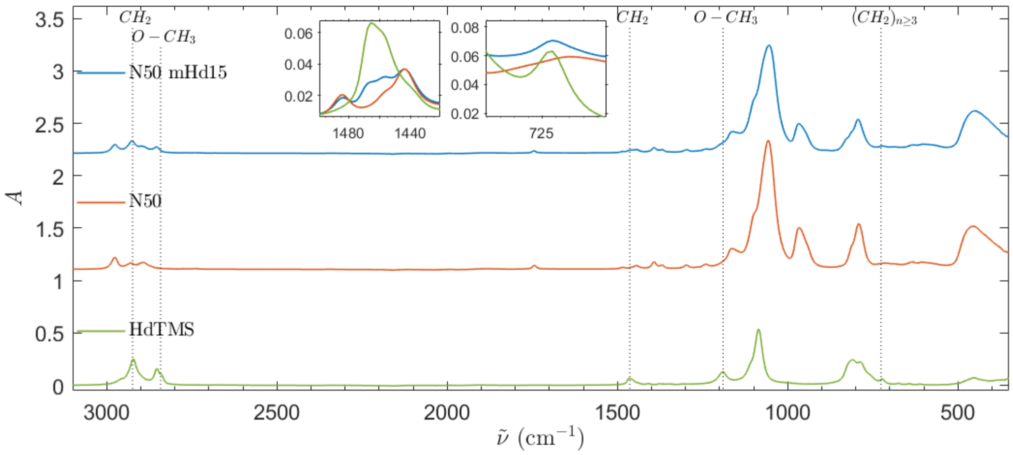

Figure S47: FTIR spectra of N50 mHd15, N50 and HdTMS with enlarged views of the ranges 1440-1480 cm^{-1} and 725 cm^{-1}.

Signals from the aliphatic chain that do not overlap with signals from polysiloxane are found at 2920, 1470 and 720 cm^{-1} in Figure S47. Signals of the methoxy group can be seen at 2830 and 1190 cm^{-1} [11].

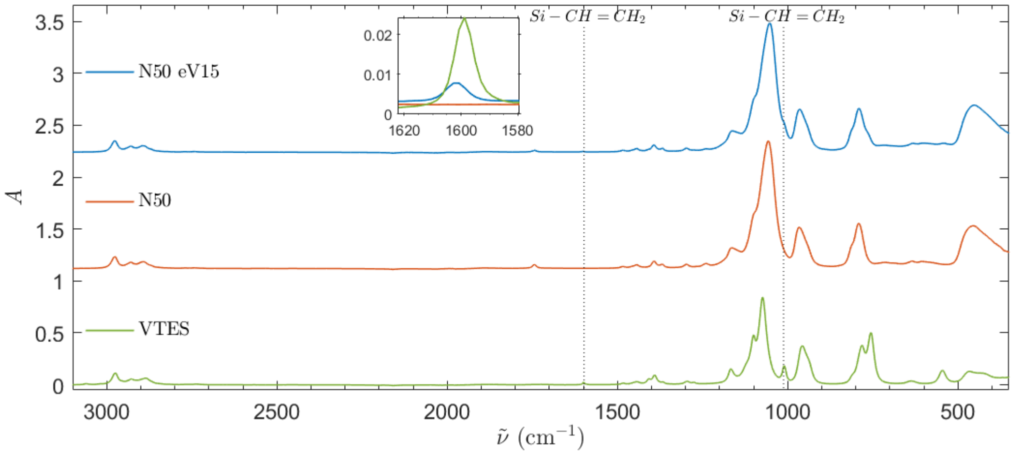

Figure S48: FTIR spectra of N50 eV15, N50 and VTES with enlarged view of the range 1580-1620 cm^{-1}.

Figure S48 shows vinyl group attached to Si that exhibits vibrations at 1600, 1410, 1010 and 960 cm^{-1}. The weak signals at 1600 cm^{-1} in the spectrum of the final funPEOS sample as well as the slight shoulder at 1010 cm^{-1} are probably attributable to the vinyl group. The other signals identified for the vinyl starting material overlap with bands of the polysiloxane[11].

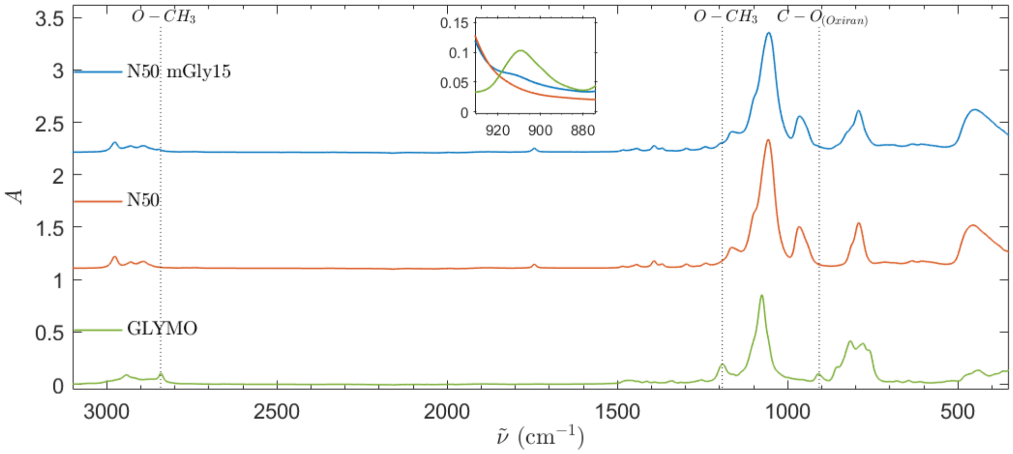

Figure S49: FTIR spectra of N50 mGly15, N50 and GLYMO with enlarged view of the range 880-920 cm^{-1}.

Figure S49 shows signal at 910 cm^{-1} which can be assigned to the vibrations of the glycidoxy ring. Other signals from this functional group overlap with the signals from polysiloxane. The signals at 1195 and 2830 cm $^{-1}$ indicate the methoxy group[11,13].

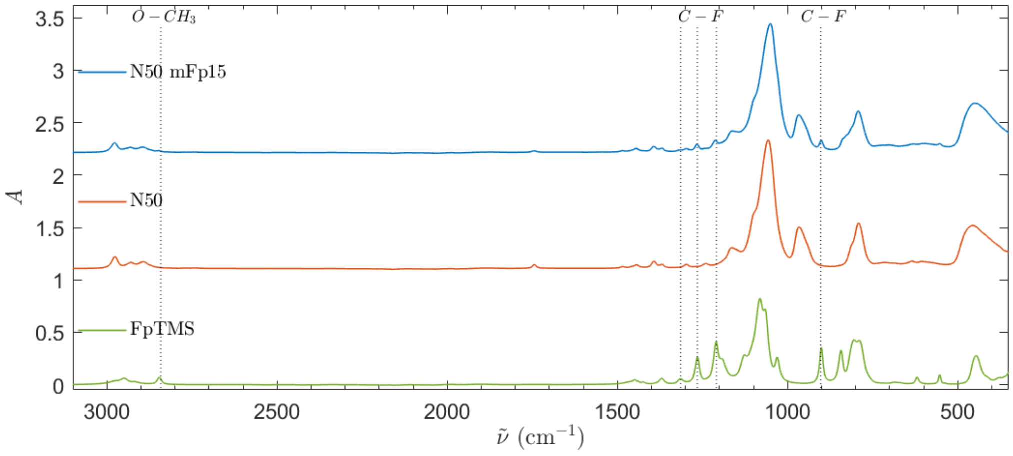

Figure S50: FTIR spectra of N50 mFp15, N50 and FpTMS.

Several C-F vibrations are assignable in the IR spectrum of the monomeric functional silane as well as in the final compound (Figure S50). The methoxy group can be detected at 2830 cm^{-1}.[11,14,15]

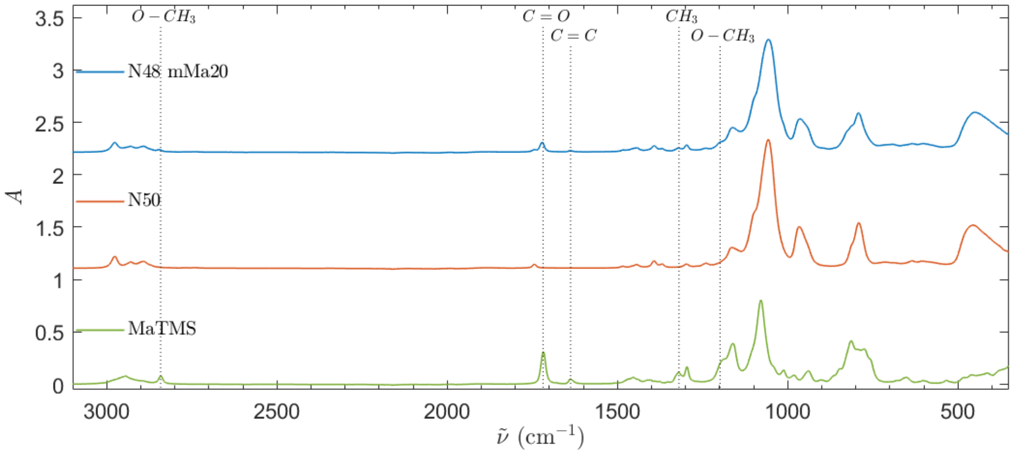

Figure S51: FTIR spectra of N48 mMa20, N50 and MaMTS.

The characteristic IR signal of the C=O group can be detected at 1710 cm^{-1} and the C=C of the methyl methacrylate group is found at 1630 cm^{-1} (Figure S51). Vibrations of the methyl group can be seen at 1310 cm^{-1} and the methoxy group at 2830 and 1195 cm^{-1} (Figure S51). [11,12]

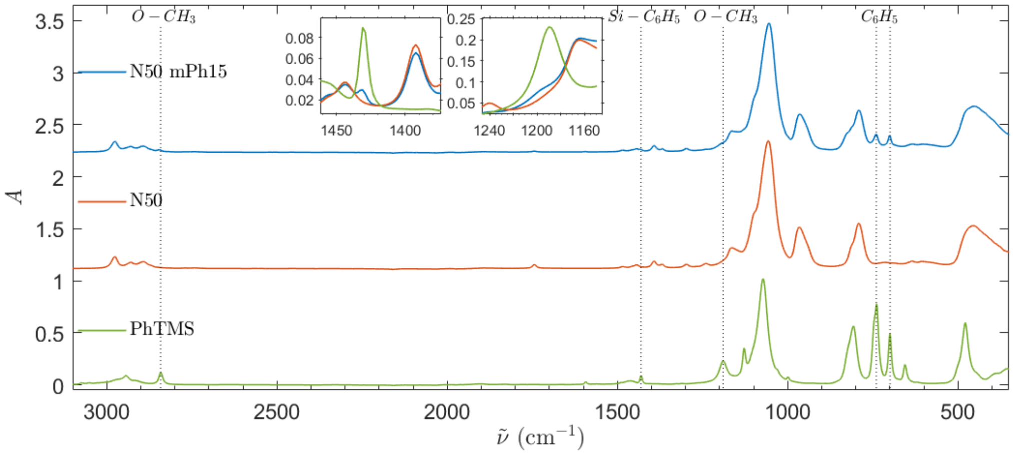

Figure S52: FTIR spectra of N50 mPh15, N50 and PhTMS with enlarged views of the ranges 1400-1450 cm^{-1} and 1160-1240 cm^{-1}.

The IR bands at 700, 730 cm^{-1} and 1430 cm^{-1} shown in Figure S52 are typical for phenyl group. The methoxy group can be seen at 2830 and 1195 cm^{-1}.[11,12]

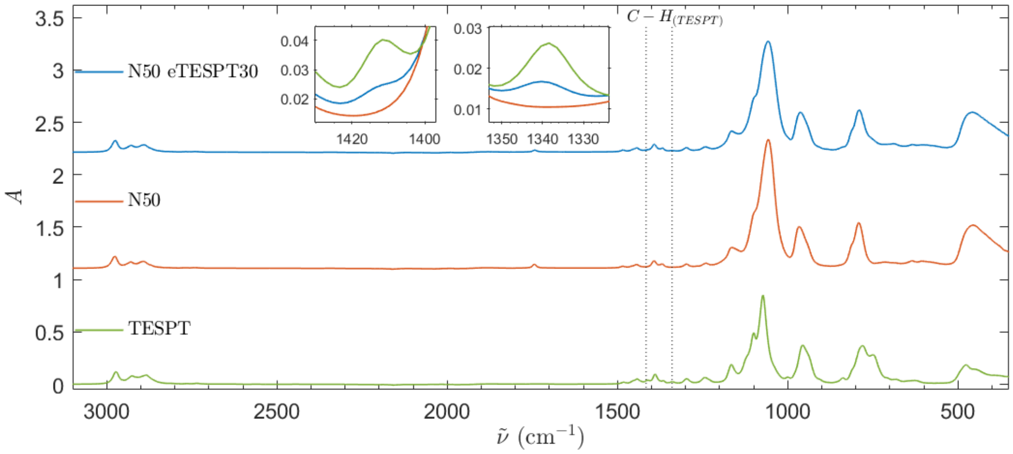

Figure S53: FTIR spectra of N50 eTESPT30, N50 and TESPT with enlarged views of the ranges 1400-1450 cm^{-1} and 1160-1240 cm^{-1}.

Typical signals for polysulfide or C-S bonds are found between 470 and 500 cm^{-1}, which in the case of the spectrum of funPEOS overlap with signals from polysiloxane N50 (Figure S53). There are 2 rather weak vibrations observable at 1410 and 1340 cm^{-1} in the region of the C-H vibrations that are observed due to the presence of TESPT [12,16].

7.3 Gel permeation chromatography

7.3.1 Double detection: refractive index detector vs. UV detector

To initially compare the results from variable wavelength detector (VWD) and refractive Index Detector (RID), N50 Ph15 was analyzed with both detector in the same run and evaluated using the same protocol.

The equations presented in Table S17 were used to calculate and compare the obtained values.

Table S17: molecular weight of the highest peak (M_p), number average molar mass (M_n), mass average molar mass (M_w) and Z-average molar mass (M_z) obtained from GPC analysis using RID and VWD.

	M_p / $\frac{\mathrm{g}}{\mathrm{mol}}$	$M_n = \frac{\sum(N_i \cdot M_i)}{\sum N_i} \frac{\mathrm{g}}{\mathrm{mol}}$	$M_w = \frac{\sum(N_i \cdot M_i^2)}{\sum(N_i \cdot M_i)} \frac{\mathrm{g}}{\mathrm{mol}}$
N50 Ph15 RID	1512	1493	2592
N50 Ph15 VWD	1339	1263	2320

Since most of the funPEOS compounds are not UV active and the direct comparison between RID and VWD showed variations of at most 10%, the refractive Index was used for detecting the signals from all the other funPEOS products.

7.3.2 GPC calibration details

<u>Narrow Column (Agilent, PLgel 5 µm; 500 – 60000 g/mol)</u>

Equations S5 to S7 used to convert retention time (t_R) to molecular weight (*Mw*) for narrow column according to fitted values from measurement of polystyrene calibration standards (measured twice) is shown below. Uncertainty values are 95 % confidence interval from the fit.

$$y(x) = a \cdot x^b \quad \text{(S 5)}$$

$$a = 1.386 \cdot 10^{15} \ (\pm 1.261 \cdot 10^{15}) \ \frac{g/mol}{min} \quad \text{(S 6)}$$

$$b = -9.64 \ (\pm 0.356) \quad \text{(S 7)}$$

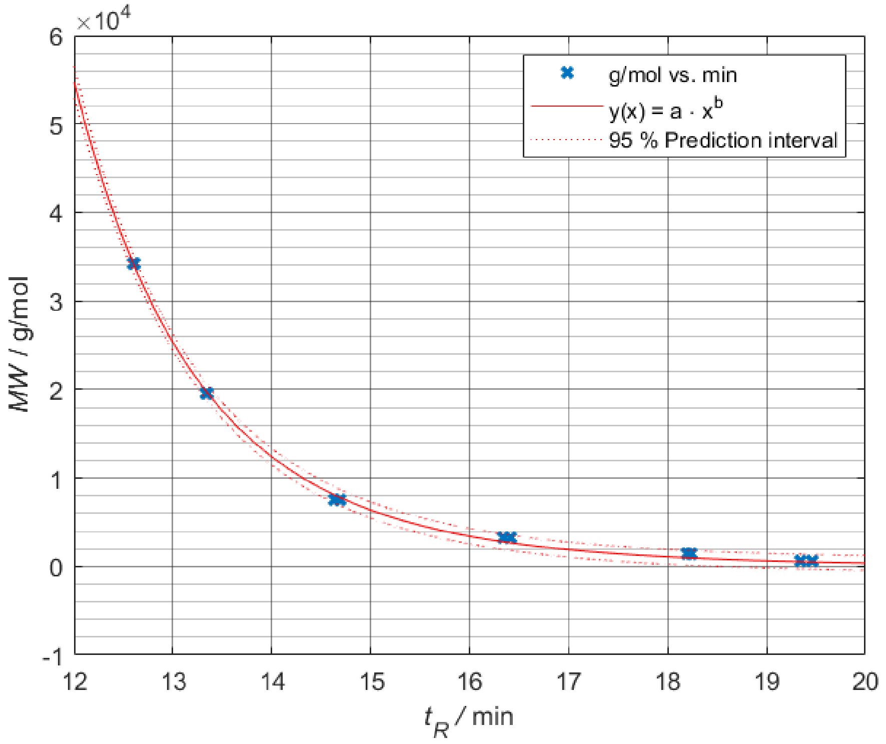

Figure S54: Molecular weight (*Mw*) vs. Retention time (t_R) using polysterene calibration standards from 685 to 19600 g/mol measured on the column 500 to 60000 g/mol. The values were fitted with a power fit and the 95 % prediction interval of the fit is shown as dashed lines above and below the main curve.

Table S18: Polystyrene calibration standard (M_p), the corresponding measured retention time (t_R) from specified column and the predicted values ($\hat{M}_p$) according to the power fit used to convert the retention time to molecular weight. Standards were measured twice, both values were used for the fit.

Mp (g/mol)	Retention time (min)		Predicted values $\hat{M}p$ (g/mol)	
685	19.35	19.46	547	519
1370	18.19	18.23	992	971
3250	16.34	16.42	2784	2669
7600	14.63	14.69	8133	7784
19600	13.33	13.35	19831	19594
34300	12.59	12.60	34432	33996

Broad column (Agilent, PLgel 5 µm mixed C; 200 – 2000000 g/mol)

Equation used to convert retention time (t_R) to molecular weight (*Mw*) for broader column according to fitted values from measurement of polystyrene calibration standard is presented here. Since the molecular weight distribution follows a linear fit, the polystyrene standards were measured once. Uncertainty values are 95 % confidence interval from the fit.

$$\log(y(x)) = a \cdot x + b \quad \text{(S 8)}$$

$$a = -0.4469\ (\pm 0.0057)\ \frac{g/mol}{min} \quad \text{(S 9)}$$

$$b = 10.87\ (\pm 0.09)\ min \quad \text{(S 10)}$$

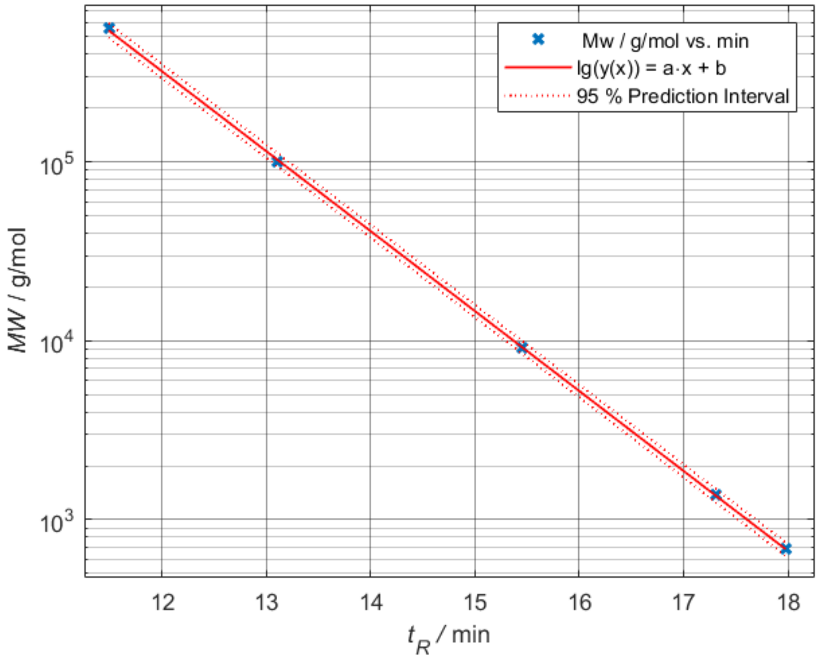

Figure S55: Molecular weight (*Mw*) vs. Retention time (t_R) using polysterene calibration standards from 685 to 556000 g/mol measured on the column 200 to 2000000 g/mol. The values were fitted with a linear fit and the 95 % prediction interval of the fit is shown is shown as dashed lines above and below the main curve.

Table S19: Polystyrene calibration standard (M_p), the corresponding measured retention time (t_R) from specified column and the predicted values ($\widehat{M}_p$) according to the power fit used to convert the retention time to molecular weight. Standards were measured once.

Mp (g/mol)	Retention time (min)	Predicted values $\widehat{M}p$ (g/mol)
685	17.98	684
1370	17.31	1362
9130	15.46	9140
99440	13.11	102598
556000	11.49	543400

8 Post-modification of funPEOS

To better understand the spectroscopic data of the post-modification reaction as described in the main part of this paper (section 3.4 Secondary modification of funPEOS), a model reaction was performed using the monomer GLYMO ((3-Glycidoxypropyl)trimethoxysilane) as starting material and, after addition of hexamethylenediamine (HMDA, dissolved in ethanol), the reaction was carried out under ambient conditions in a 1:1 molar ratio.[17].

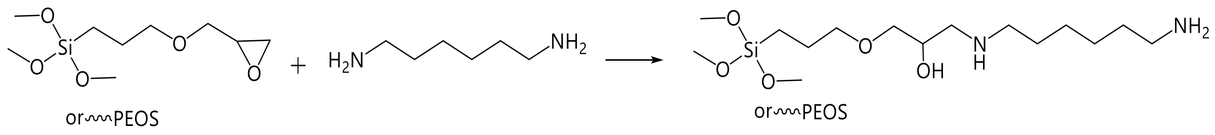

Figure S56: Amine addition to monomeric GLYMO or to glycidoxypropyl-functionalized substrate in a ring-opening reaction.

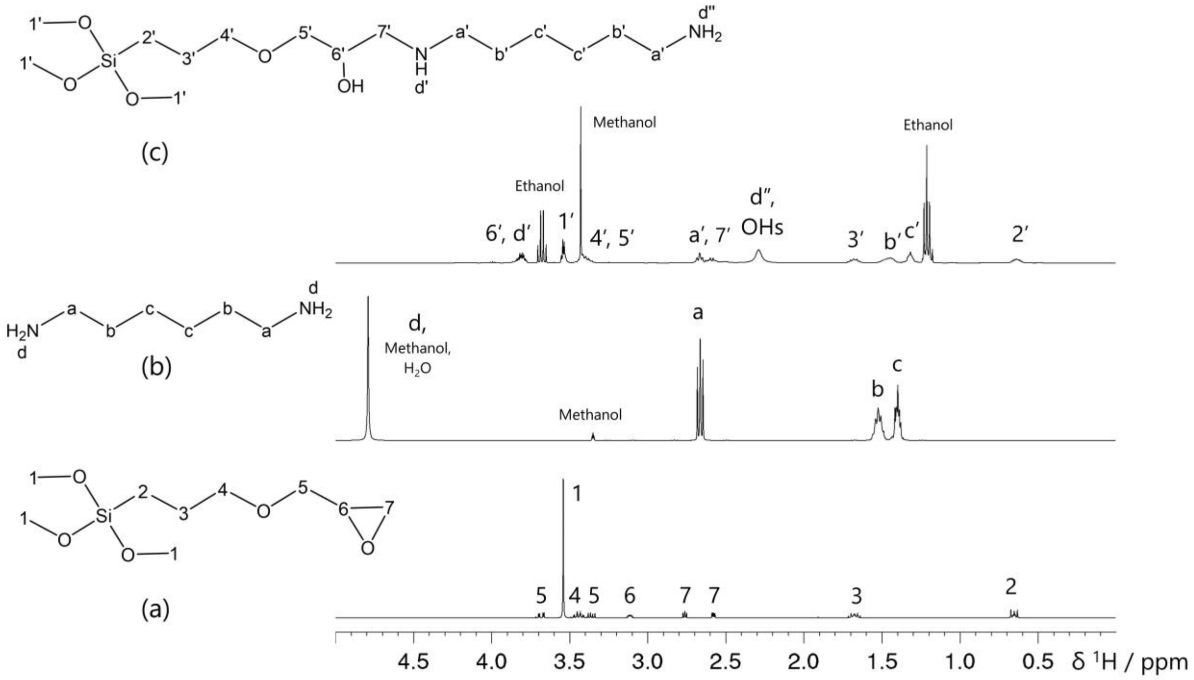

Figure S57: ^{1}H NMR spectra of (a) GLYMO in $CDCl_3$, (b) HMDA in methanol-D4 and (c) GLYMO modified with HMDA in $CDCl_3$.

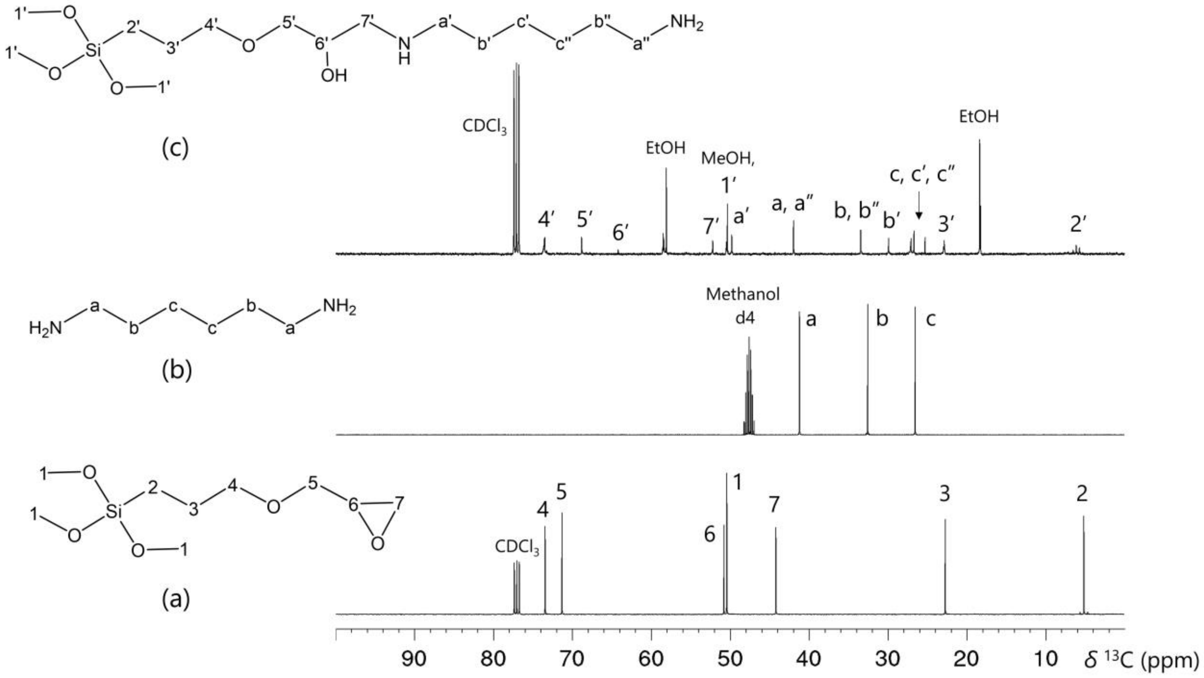

Figure S58: ^{13}C NMR spectra of (a) GLYMO, (b) HMDA and (c) GLYMO modified with HMDA.

^{1}H NMR of amino modified GLYMO (Figure S57) shows the evidence of ring opening of the GLYMO epoxide with the diamine through changes in the signal of proton 6, indicating a change in chemical environment similar to that in the post-modified funPEOS product, and the signals of the newly generated bonds in the modified product[17]. The ^{1}H NMR signal for 6' is assigned to the peak at 3.81 ppm on the basis of the adjacent alcohol group having a shift that is 0.7 ppm higher than the epoxide shift of proton 6, assigned to 3.11 ppm. This also indicates the presence of the primary amine addition product, further supported by the distinguishable protons resonating from 7 only showing one signal in the product and their respective integrals. This spectrum also suggests that the modification with the diamine leads to minor changes in terms of chemical shift for protons adjacent to either the epoxide or newly formed amine. GLYMO has been used in this example for the purpose of modelling the reaction in a monomeric fashion prior to carrying it out with the polysiloxanes with the goal of simpler peak identification and more clear assignments. Due to the low functionality percentage of the shell in polysiloxane compounds, ^{1}H NMR signals are very weak and at times less informative than in a monomeric system.

^{13}C NMR spectrum of the amino modified GLYMO depicted on Figure S58 is in agreement with the ^{1}H NMR spectrum assignment and confirms that the ring opening reaction took place.

To investigate the presence of methanol signal in the ^{1}H and ^{13}C NMR of amino modified GLYMO as well as the amino modified N50 mGly15, we carried out ^{29}Si NMR of amino modified GLYMO product. This spectrum (Figure S59) shows that originally monomeric GLYMO reacted further via hydrolysis-condensation reaction to produce higher T silanes (T^1 and T^2) and side-product methanol due to basic conditions introduces by the diamine.

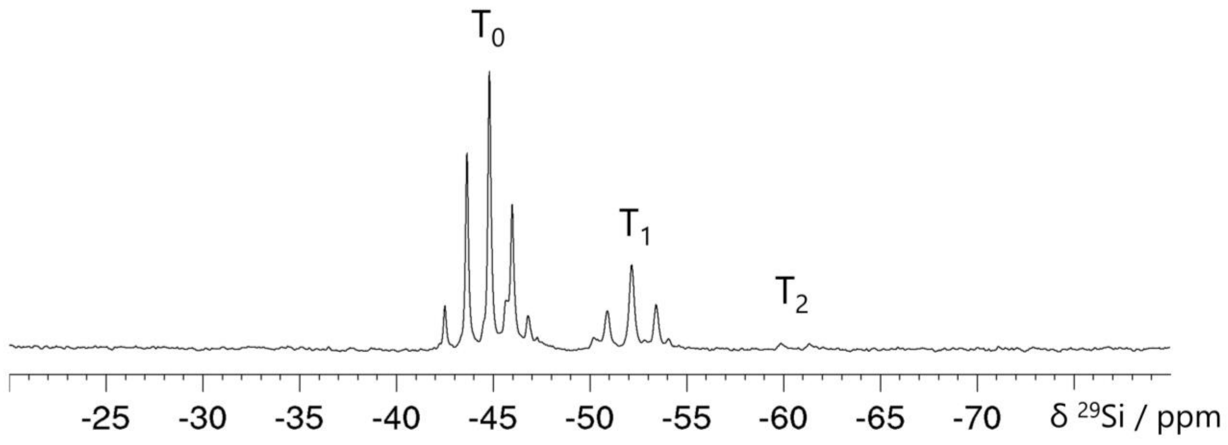

Figure S59: ^{29}Si NMR spectrum obtained from GLYMO modified with HMDA.

9 Preparation of silica foam

Figure 9 b in the main text shows a silica foam that was prepare using the following recipe. 23.0 ml of water, 53.3 ml of ethanol/IPA (95 % / 5 %) mixture, 5.0 ml of MTES, 2.5 ml of VTES, 1.1 ml of N60 eM15 and 3.8 ml N45 eV30 were mixed for 15 minutes at 60°C. 8.3 ml of DMDES was added to the solution and was left to stir for another 75 minutes. 80 mg of ammonium persulfate and 0.15 ml of concentrated ammonia solution were added to the sol before it was poured into the steel containers and aged for 16 hours at 65 °C.

References

(1) Kelts, L. W.; Armstrong, N. J. A Silicon-29 NMR Study of the Structural Intermediates in Low PH Sol-Gel Reactions. *Journal of Materials Research* **1989**, *4*, 423–433. https://doi.org/10.1557/JMR.1989.0423.

(2) Pouxviel, J. C.; Boilot, J. P.; Beloeil, J. C.; Lallemand, J. Y. NMR Study of the Sol/Gel Polymerization. *Journal of Non-Crystalline Solids* **1987**, *89* (3), 345–360. https://doi.org/10.1016/S0022-3093(87)80277-6.

(3) Brochier Salon, M.-C.; Belgacem, M. N. Hydrolysis-Condensation Kinetics of Different Silane Coupling Agents. *Phosphorus, Sulfur, and Silicon and the Related Elements* **2011**, *186* (2), 240–254. https://doi.org/10.1080/10426507.2010.494644.

(4) Wang, S.-J.; Fan, X.-D.; Si, Q.-F.; Kong, J.; Liu, Y.-Y.; Qiao, W.-Q.; Zhang, G.-B. Preparation and Characterization of a Hyperbranched Polyethoxysiloxane Based Anti-Fouling Coating. *Journal of Applied Polymer Science* **2006**, *102* (6), 5818–5824. https://doi.org/10.1002/APP.24842.

(5) Brochier Salon, M.-C.; Bayle, P.-A.; Abdelmouleh, M.; Boufi, S.; Belgacem, M. N. Kinetics of Hydrolysis and Self Condensation Reactions of Silanes by NMR Spectroscopy. *Colloids and Surfaces A: Physicochemical and Engineering Aspects* **2008**, *312* (2–3), 83–91. https://doi.org/10.1016/j.colsurfa.2007.06.028.

(6) Sugahara, Y.; Inoue, T.; Kuroda, K. 29Si NMR Study on Co-Hydrolysis Processes in Si(OEt)4–RSi(OEt)3 –EtOH–Water–HCl Systems (R=Me, Ph): Effect of R Groups. *Journal of Materials Chemistry* **1997**, *7* (1), 53–59. https://doi.org/10.1039/a603741k.

(7) Jaumann, M.; Rebrov, E. A.; Kazakova, V. V.; Muzafarov, A. M.; Goedel, W. A.; Möller, M. Hyperbranched Polyalkoxysiloxanes via AB3-Type Monomers. *Macromolecular Chemistry and Physics* **2003**, *204* (7), 1014–1026. https://doi.org/10.1002/macp.200390067.

(8) Zhu, X.; Jaumann, M.; Peter, K.; Möller, M.; Melian, C.; Adams-Buda, A.; Demco, D. E.; Blumich, B. One-Pot Synthesis of Hyperbranched Polyethoxysiloxanes. *Macromolecules* **2006**, *39* (5), 1701–1708. https://doi.org/10.1021/ma052179.

(9) de Buyl, F.; Kretschmer, A. Understanding Hydrolysis and Condensation Kinetics of γ-Glycidoxypropyltri-methoxysilane. *Journal of Adhesion* **2008**, *84* (2), 125–142. https://doi.org/10.1080/00218460801952809.

(10) Rubio, F.; Rubio, J.; Oteo, J. L. A FT-IR Study of the Hydrolysis of Tetraethylorthosilicate (TEOS). *Spectroscopy letters* **1998**, *31* (1), 199–219. https://doi.org/10.1080/00387019808006772.

(11) Launer, P. Infrared Analysis of Organosilicon Compounds. *Silicon Compounds: Silanes & Silicones*; **1987**; pp 100–103.

(12) Coates, J. Interpretation of Infrared Spectra, A Practical Approach. In *Encyclopedia of Analytical Chemistry: Applications, Theory and Instrumentation*; American Cancer Society, **2006**; pp 1–23. https://doi.org/10.1002/9780470027318.A5606.

(13) Šapić, I. M.; Bistričić, L.; Volovšek, V.; Dananić, V. Vibrational Analysis of 3-Glycidoxypropyltrimethoxysilane Polymer. *Macromolecular Symposia* **2014**, *339* (1), 122–129. https://doi.org/10.1002/MASY.201300145.

(14) Giasuddin, A. B. M.; Cartwright, A.; Britt, D. W. Silica Nanoparticles Synthesized from 3,3,3-Propyl(Tri-fluoro)Trimethoxysilane or n-Propyltrimethoxysilane for Creating Superhydrophobic Surfaces. *ACS applied nano materials* **2021**, *4* (4), 4092–4102. https://doi.org/10.1021/ACSANM.1C00398.

(15) Brassard, J.-D.; Sarkar, D. K.; Perron, J. Synthesis of Monodisperse Fluorinated Silica Nanoparticles and Their Superhydrophobic Thin Films. *ACS Applied Materials & Interfaces* **2011**, *3* (9), 3583–3588. https://doi.org/10.1021/AM2007917.

(16) Marrone, M.; Montanari, T.; Busca, G.; Conzatti, L.; Costa, G.; Maila Castellano; Turturro, A. A Fourier Transform Infrared (FTIR) Study of the Reaction of Triethoxysilane (TES) and Bis[3-Triethoxysilylpropyl]Tetrasulfane (TESPT) with the Surface of Amorphous Silica. *Journal of Physical Chemistry B* **2004**, *108* (11), 3563–3572. https://doi.org/10.1021/JP036148X.

(17) Stropoli, S. J.; Elrod, M. J. Assessing the Potential for the Reactions of Epoxides with Amines on Secondary Organic Aerosol Particles. *Journal of Physical Chemistry A* **2015**, *119* (40), 10181–10189. https://doi.org/10.1021/acs.jpca.5b07852.
